# Efficacy and Safety in Proton Therapy and Photon Therapy for Patients With Esophageal Cancer: A Meta-Analysis

**DOI:** 10.1001/jamanetworkopen.2023.28136

**Published:** 2023-08-15

**Authors:** Pixiao Zhou, Yangfeng Du, Ying Zhang, Mei Zhu, Ting Li, Wei Tian, Tao Wu, Zemin Xiao

**Affiliations:** 1 Department of Oncology, Changde Hospital, Xiangya School of Medicine, Central South University, Changde, China; 2 The Second People’s Hospital of Yibin, Yibin, China

## Abstract

**Importance:**

Radiotherapy plays an important role in the treatment of esophageal cancer. Proton therapy has unique physical properties and higher relative biological effectiveness. However, whether proton therapy has greater benefit than photon therapy is still unclear.

**Objective:**

To evaluate whether proton was associated with better efficacy and safety outcomes, including dosimetric, prognosis, and toxic effects outcomes, compared with photon therapy and to evaluate the efficacy and safety of proton therapy singly.

**Data Sources:**

A systematic search of PubMed, Embase, the Cochrane Library, Web of Science, SinoMed, and China National Knowledge Infrastructure databases was conducted for articles published through November 25, 2021, and updated to March 25, 2023.

**Study Selection:**

For the comparison of proton and photon therapy, studies including dosimetric, prognosis, and associated toxic effects outcomes were included. The separate evaluation of proton therapy evaluated the same metrics.

**Data Extraction and Synthesis:**

Data on study design, individual characteristics, and outcomes were extracted. If *I*^2^ was greater than 50%, the random-effects model was selected. This meta-analysis is reported following the Preferred Reporting Items for Systematic Reviews and Meta-analyses (PRISMA) reporting guideline.

**Main Outcomes and Measures:**

The main outcomes were organs at risk (OARs) dosimetric outcomes, prognosis (overall survival [OS], progression-free survival [PFS], and objective response rate [ORR]), and radiation-related toxic effects.

**Results:**

A total of 45 studies were included in the meta-analysis. For dosimetric analysis, proton therapy was associated with significantly reduced OARs dose. Meta-analysis showed that photon therapy was associated with poor OS (hazard ratio [HR], 1.31; 95% CI, 1.07-1.61; *I*^2^ = 11%), but no difference in PFS was observed. Subgroup analysis showed worse OS (HR, 1.42; 95% CI, 1.14-1.78; *I*^2^ = 34%) and PFS (HR, 1.48; 95% CI, 1.06-2.08; *I*^2^ = 7%) in the radical therapy group with photon therapy. The pathological complete response rate was similar between groups. Proton therapy was associated with significantly decreased grade 2 or higher radiation pneumonitis and pericardial effusion, and grade 4 or higher lymphocytopenia. Single-rate analysis of proton therapy found 89% OS and 65% PFS at 1 year, 71% OS and 56% PFS at 2 years, 63% OS and 48% PFS at 3 years, and 56% OS and 42% PFS at 5 years. The incidence of grade 2 or higher radiation esophagitis was 50%, grade 2 or higher radiation pneumonitis was 2%, grade 2 or higher pleural effusion was 4%, grade 2 or higher pericardial effusion was 3%, grade 3 or higher radiation esophagitis was 8%, and grade 4 or higher lymphocytopenia was 17%.

**Conclusions and Relevance:**

In this meta-analysis, proton therapy was associated with reduced OARs doses and toxic effects and improved prognosis compared with photon therapy for esophageal cancer, but caution is warranted. In the future, these findings should be further validated in randomized clinical trials.

## Key Points

**Question:**

What are efficacy and safety profiles associated with proton therapy vs photon therapy for esophageal cancer?

**Findings:**

In this meta-analysis including 45 studies, proton therapy was associated with significantly reduced irradiation doses to organs at risk, and the incidence of grade 2 or higher radiation pneumonitis and pericardial effusion and grade 4 or higher lymphocytopenia. Photon therapy was associated with poor OS and PFS compared with proton therapy.

**Meaning:**

These findings suggest that proton therapy may be more effective and safer than photon therapy for patients with esophageal cancer.

## Teaser

This meta-analysis evaluates whether proton therapy is associated with better efficacy and safety outcomes, including dosimetry, prognosis, and toxic effects, compared with photon therapy in patients with esophageal cancer.

## Introduction

Esophageal cancer is one of the most common malignant tumors of the digestive tract, is the sixth leading cause of cancer-related death worldwide, and represents a tremendous challenge to global health.^1^ As there are no obvious positive symptoms in the early stage among patients with typical progressive dysphagia or other symptoms, the tumor mostly progresses to the middle-advanced stage, with low survival rates and poor 5-year overall survival (OS) (<20%).^2,3^ For patients with surgically resectable disease with a local progress stage, neoadjuvant chemoradiotherapy followed by surgery is the standard treatment regimen. For patients with inoperable disease, radical concurrent chemoradiotherapy is the priority recommended treatment.^1,3^ Theoretically, the local control rate is related to the irradiation dose of tumor target area, and the presence of radiation-related toxic effects limits the further improvement of the prescription dose and affects the patient’s quality of life and prognosis.

The unique physical properties of proton therapy (ie, Bragg peak) allow the radiation dose with the maximum concentration on the tumor target area, thus effectively reducing the dose to organs at risk (OARs) and the incidence of radiation damage.^4^ In addition, the higher relative biological effectiveness of proton therapy might have the potential to improve survival outcomes further. Unfortunately, a randomized clinical trial (RCT) in patients with non–small-cell lung cancer (NSCLC) did not observe a reduction in the incidence of radiation pneumonitis (RP) with proton therapy compared with intensity-modulated radiotherapy (IMRT).^5^ Previous studies have also reported that proton therapy can improve local control and disease-free survival in patients with NSCLC.^6,7^ However, to our knowledge, there is no definitive evidence to support the efficacy and safety of proton therapy in esophageal cancer to support clinical decision-making.

## Methods

This meta-analysis followed the Preferred Reporting Items for Systematic Reviews and Meta-analyses (PRISMA) reporting guideline. This study has been prospectively registered at PROSPERO (CRD42021293449).

### Search Strategy

Electronic searches were conducted on PubMed, Embase, the Cochrane Library, Web of Science, SinoMed, and China National Knowledge Infrastructure for eligible studies published before November 25, 2021. We also conducted a forward search of included studies until March 25, 2023. The search strategy conducted in PubMed, Embase, and Cochrane combined Medical Subject Headings and free-text words. A similar search strategy was performed in Embase but transformed according to the database’s subject headings. Keywords searches were used for the remaining other databases. Moreover, the reference lists from relevant studies were manually searched for potentially eligible articles. The complete search strategies for each database are available in the eAppendix in Supplement 1).

### Inclusion and Excluded Criteria

This study had 2 objectives. The first objective was to evaluate whether proton therapy was beneficial compared with photon therapy (including the dosimetric, prognosis, and toxic effects outcomes). The second objective was to assess the efficacy and safety of proton therapy using single-rate pooled analysis.

For the comparison of proton and photon therapy, inclusion criteria included for efficacy evaluation metrics were studies reporting objective response rate (ORR), OS, or progress-free survival (PFS). Safety metrics were cardiopulmonary toxic effects, myelotoxic effects, and esophageal toxic effects. For the dosimetric comparison analysis, the OARs were the lungs, heart (including substructures), spinal cord (SC), and bone marrow. If multiple proton techniques were available simultaneously in 1 study, the technique with the greatest comprehensive dose benefit to OARs was preferred. For the separate evaluation of proton therapy, the efficacy and safety evaluation metrics were same.

**Figure 1.**
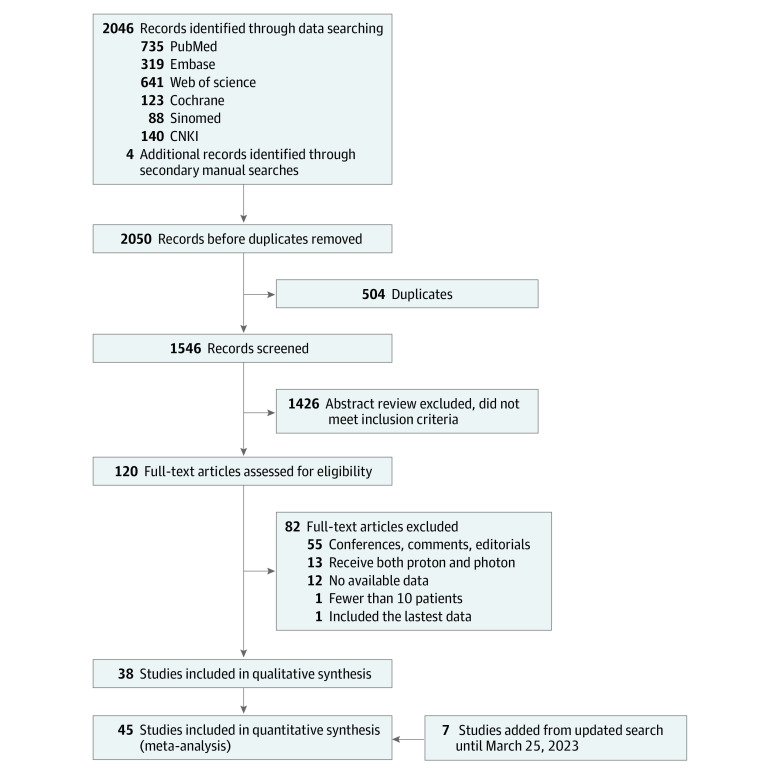
Flowchart of the Study Screening and Evaluation Process

Finally, all studies had to include at least 10 patients. Studies with mixed-disease assessment were not included unless data related to esophageal cancer could be extracted separately. Additionally, studies among patients who had received radiotherapy previously were excluded, nor could patients receive proton and photon therapy simultaneously, except for salvage-therapy after recurrence. We only included the most recently published publications from the same cohort for the same metric. Editorials, letters, comments, case reports, reviews, meta-analyses, and conference abstracts were excluded (Figure 1).

### Data Collection

From all included studies, we collected basic information, including author, year of publication, the number of patients, and patient characteristics, including target location, radiotherapy techniques, and prescription dose. For dosimetric comparison studies, also including OARs dose-limitation regimens, we collected the mean percentage benefit of OARs dosimetric parameters and planning quality. For efficacy and safety evaluation studies, we collected patient characteristics and also included age, study type, stage, pathology type, radiotherapy modalities (neoadjuvant or radical), chemotherapy regimens, median follow-up time, prognosis, and toxic effects. Toxic effects reported within 3 months after the end of radiotherapy were defined as acute toxic effects; all others were considered late toxic effects. If the data were only presented by graphs, Graph Digitizer software version 2.25 (GetData) was used to extract numerical values. Not all dosimetric comparison studies provided means with SDs, but if studies described median, range, or IQR, we used the methods proposed by Wan et al^8^ to estimate means. In terms of prognosis analysis, the OS and PFS were measured by hazard ratios (HRs) and 95% CIs that were directly reported in the included studies. When studies did not report HR but presented Kaplan-Meier survival curves instead, we obtained estimated HR from the curves by using a calculation spreadsheet developed by Tierney and colleagues.^9^

### Quality Assessment

For quality assessments, we used the Newcastle-Ottawa Scale for non-RCT studies, Methodological Index for Nonrandomized Studies for single-group studies, and the Cochrane Collaboration tool for RCTs. Two authors then collaborated to identify eligible studies (Y.D. and T.L.), collect data (P.Z. and Y.Z.), and assess quality (M.Z. and W.T.). Any disagreement was resolved by discussion or the third reviewer (T.W. or Z.X.).

### Statistical Analysis

For proton therapy single-rate analysis, we calculated the single ratios and integrated ratios with 95% CIs. Furthermore, if the distribution type of a single rate did not conform to a normal distribution, it was transformed using double-arcsine transformations to improve the reliability of the combined results. Heterogeneity was assessed by *I*^2^ statistic, with *I*^2^ greater than 50% considered significant. Random-effects models were more applicable to mitigate heterogeneity than fixed-effects models, so if *I*^2^ was greater than 50%, the random-effects model was selected. Publication biases were assessed using visual inspection of funnel plots and quantitatively assessed using Egger test (when there were ≥10 included studies). A nonparametric trim-and-fill method was also applied to minimize the influence of publication bias on the results. Sensitivity analysis was performed by deleting each study individually. All analysis were conducted in RevMan version 5.4 (Cochrane) or Stata version 17.0 (StataCorp). *P* values were 2-sided, and *P* < .05 was considered significant.

## Results

A total of 45 studies met the inclusion criteria and were included in the meta-analysis.^4,10,11,12,13,14,15,16,17,18,19,20,21,22,23,24,25,26,27,28,29,30,31,32,33,34,35,36,37,38,39,40,41,42,43,44,45,46,47,48,49,50,51,52,53^ Figure 1 describes the selection process and the reasons for excluding studies. Among the included studies, 31 studies^10,11,12,13,14,15,16,17,18,19,20,21,22,23,24,25,27,28,29,31,34,35,37,38,46,47,49,50,51,52^ included dosimetric comparison (eTable 1 in Supplement 1), 17 studies^25,26,27,28,29,30,31,32,33,34,35,36,37,38,46,48,49^ assessed proton vs photon therapy (Table 1), and 10 studies^4,16,39,40,41,42,43,44,45,53^ were single-group studies of proton therapy (Table 2). Three studies^32,33,36^ used propensity score–matched analysis to control for confounding factors, and results from those analyses were used for data synthesis. Literature quality evaluations are presented in eTables 2-5 in Supplement 1.

**Table 1.** Description of Prognosis and Toxic Effects After Treatment With Proton or Photon Radiotherapy in Patients With Esophageal Cancer

Source	Patients, No.		Study Type	Characteristics (proton vs photon)				RT technology/prescription dose (proton vs photon)	Follow-up, median, mo	Outcome (proton vs photon)	Toxic effects
	Proton	Photon		Age, median, y	Stage	Thoracic site	Pathological type				
Suh et al,^25^ 2021	48	29	RO	69 vs 73	cT1-3N0M0	Lower: 58% vs 28%^a^	SCC: 100%	PBT vs 3D-CRT/IMRT (24/5); Median PTV: 66 Gy/33 F; Radical RT (CCRT: 17% vs 31%) and salvage-therapy (33.8%)	46	OS: 1 y, 100% vs 97%; 2 y, 88% vs 79%; 3 y, 69% vs 69%; 5 y, 69% vs 62%^b^; PFS: 1 y, 88% vs 93%; 2 y, 70% vs 74%; 3 y, 63% vs 66%; 5 y, 57% vs 58%^b^	Acute: G≥2: RE, 29.9%; RP, 1.3% G≥3: PE, 0% vs 0%; PCE, 0% vs 0%
Ebrahimi et al,^26^ 2021	30	15	RO	NA	NA	NA	NA	PBT (IMPT/PSPT) vs IMRT; PTV: 50.4 Gy/28 F; Radical CCRT	NA	NA	Mean minimum ALC: 390/330/μL vs 170/μL; change in ALC: 970/1080/μL vs 1250/μL
Sumiya et al,^27^ 2021	54	15	RO	70	I-IV (III-IV: 41% vs 80%)^a^	Middle and lower: 80% vs 67%	SCC: 96% vs 93%	PBT vs PRT; PTV: 56-70 Gy/28-35 F; Radical CCRT (43% vs 52%: 5-FU + cisplatin)	NA	OS: 1 y, 86% vs 60%; 2 y, 77% vs 48%^a^^,^^b^ PBT OS: 3 y, 75%; 5 y, 75%	Late: G≥2: RP, 0% vs 0%; PE, 5.6% vs 0%; PCE, 7.4% vs 13.3%; G≥3: LT and HET, 0%
Lin et al,^28^ 2020	46	61	RCT (II)	67 vs 67	I-III (III: 59% vs 54%)	Lower: 83% vs 84%	AC: 91% vs 87%	PBT (80% PSPT) vs IMRT; PTV: 50.4 Gy/28 F: 87% vs 95%; Neoadjuvant and radical CCRT (61% vs 51%: 5-FU/capecitabine + taxane) + surgery (46% vs 49%)	44.1	OS: 1 y, 79% vs 89%; 2-y, 60% vs 66%; 3 y, 51% vs 53%; 5 y, 47% vs 51%^b^; PFS: 1 y, 56% vs 60%; 2 y, 50% vs 45%; 3 y, 45% vs 45%; 5 y, 41% vs 41%^b^; pCR: 14 vs 18	Acute: G≥2: RP, 2.2% vs 4.9%; G≥3: RE, 13% vs 13%; RP, 0% vs 2%; PE, 0% vs 5%; PCE, 0% vs 2%; lymphocytopenia, 0% vs 2%; G≥4: lymphocytopenia, 27% vs 52%^a^
Wang et al,^29^ 2020	159	320	PO	62	I-III	Lower: 89%	AC: 87.1%	PBT (91% PSPT) vs IMRT; Median PTV: 50.4 Gy/28 F; Neoadjuvant and radical CCRT (FU+ platinum- and taxane-based) + surgery (59.3%)	76	NA	Late: G≥3: 2-y HET, 11% vs 18%; 3-y HET, 13% vs 21%
DeCesaris et al,^30^ 2020	18	36	RO	62	II-IV (II: 33% vs 61%)^a^	Middle and lower: 100%	AC: 100%	PBT (IMPT) vs PRT (4-D CT); Median PTV: 50.4 Gy/28 F; Neoadjuvant CCRT (98%: carboplatin + paclitaxel) + surgery	25	OS: 1 y, 93% vs 72%; 2 y, 71% vs 55%^b^ pCR: 3 vs 8	Acute: G≥2: RE, 33% vs 28%; HT, 6% vs 17%; G≥4, 0%
Bhangoo et al,^31^ 2020	32	32	RO	71.5 vs 71.4	T1-3N0-3M0 (T3: 63% vs 56%)	Lower: 78% vs 94%	AC: 63% vs 91%^a^	PBT (IMPT) vs IMRT (4-D CT); Median PTV: 50 Gy/25 F; Neoadjuvant and radical CCRT (91% vs 100%: carboplatin + paclitaxel) + surgery (47% vs 56%)	10 vs 14	OS: 1 y, 74% vs 71%; 2 y, 40% vs 67%^b^ PFS: 1 y, 71% vs 46%; 2 y, 60% vs 37%^b^ pCR: 5 vs 7	Acute: G≥2: RE, 41% vs 25%; lymphocytopenia, 100% vs 94%; G≥3: RE, 3% vs 0%; lymphocytopenia, 84% vs 81%; G≥4: lymphocytopenia, 19% vs 28%
Routman et al,^32^ 2019	50	50^c^	RO	66 vs 64.5	I-IV (III-IV: 61% vs 37%)^a^	Lower: 88% vs 90%	AC: 88% vs 86%	PBT (IMPT) vs 3D-CRT/VMAT Median PTV: 50 Gy/25 F; Radical CCRT (carboplatin + paclitaxel) + surgery (88%)	NA	NA	Acute: G≥4 lymphocytopenia, 24.0% vs 60.0%^a^
Shiraishi et al,^33^ 2018	136	136^c^	RO	63 vs 60	I-IV (III-IV: 64% vs 60%)	Lower: 96% vs 97%	AC: 96% vs 98%	PBT vs IMRT (4-D CT); PTV: 50.4 Gy/28 F; Neoadjuvant CCRT (80.2%: FU + taxane/platinum) + surgery	NA	NA	Acute: G≥4 lymphocytopenia, 17.6% vs 40.4%^a^
Macomber et al,^34^ 2018	16	39	RO	62	II-III (III: 56% vs 70%)	Lower: 78% vs 84%	AC: 94% vs 76%^a^	PBT vs 3-D CRT/IMRT (16/21); PTV: 50.4 Gy/28 F; Neoadjuvant CCRT (100% vs 78%: carboplatin + paclitaxel) + surgery	20	Overall OS: 1 y, 92%; 2 y, 77% pCR: 3 vs 8	NA
Xi et al,^35^ 2017	132	211	RO	≥67: 71% vs 38%^a^	I-III (III: 64% vs 67%)	Lower: 71% vs 73%	AC: 68% vs 74%	PBT (95% PSPT) vs IMRT; Median PTV: 50.4 Gy/28 F; Radical CCRT (FU+ platinum/taxane) + salvage surgery (7.6% vs 12.8%)	44.8 vs 65.1	OS: 1 y, 87% vs 84%; 2 y, 67% vs 52%; 3 y, 55% vs 39%; 5 y, 42% vs 32%^a^^,^^b^ PFS: 1 y, 62% vs 48%; 2 y, 49% vs 32%; 3 y, 43% vs 28%; 5 y, 35% vs 20%^a^^,^^b^	G≥2: RE, 45.5% vs 46.0%; RP, 3.9% vs 6.7%; PE, 5.3% vs 6.6%; PCE, 0.8% vs 2.4%; G≥3: RE, 11.4% vs 14.7%; RP, 1.6% vs 2.9%; PE, 0.8% vs 1.9%; PCE, 0.8% vs 2.4%; G≥4: RE, 0% vs 0.5%; RP, 0.8% vs 1.0%; PE, 0% vs 0%; PCE 0% vs 0%
Fang et al,^36^ 2018	110	110^c^	RO	70 vs 69	I-IV (III-IV: 61% vs 60%)	Lower: 76.4% vs 76.4%	AC: 72% vs 76%	PBT vs IMRT (4-D CT); Median PTV: 50.4 Gy/28 F; Radical CCRT	NA	Stage III-IV OS: 1 y, 73% vs 75%; 2 y, 66% vs 49%; 3 y, 47% vs 38%; 5 y, 44% vs 16%^b^	Acute: G≥4 lymphocytopenia, 30.9% vs 47.3%^a^
Lin et al,^37^ 2017	111	469	RO	>65: 32% vs 36%/ 26%	III-IV: 64% vs 63%/64%	Lower: 98% vs 88%/95%^a^;	AC: 96% vs 90%/94%;	PBT vs 3D-CRT/IMRT (214/ 255); PTV: 50.4 Gy/28 F; Neoadjuvant CCRT + surgery	NA	NA	Acute: LT, 16.2% vs 39.5%/24.2%; HET, 11.7% vs 11.7%/27.4%
Makishima et al,^38^ 2015	25	19	PO	NA	0-III (III: 36% vs 74%)^a^	88% vs 63%	SCC: 100%	PBT vs 3D-CRT; Median PTV: 60 Gy/30 F; Radical CCRT (5-FU + cisplatin)	24 vs 20	NA	Late: G≥2: RP, 0% vs 21.1%; PE, 0% vs 10.6%; PCE, 4% vs 52.6%; G≥3: RP, 0% vs 5.3%; PE, 0% vs 5.3%; PCE, 0% vs 0%; G≥4: RP, 0% vs 0%; PE, 0% vs 0%; PCE, 0% vs 0%
Zhu et al,^46^ 2021	246	500	RO	65 vs 62^a^	I-III (III: 65% vs 64%)	Lower: 88% vs 86%	NA	PBT vs PRT; PTV: 50.4 Gy/NA; Neoadjuvant and radical CCRT (platinum; or taxane-based)	NA	NA	Acute: G≥4 lymphocytopenia, 22.0% vs 46.2%^a^
Lin et al,^48^ 2022	81	156	RO	61	I-V (III-IV: 34% vs 66%)	NA	NA	PBT vs IMRT; PTV: 50.4 Gy/28 F; Neoadjuvant CCRT + surgery	NA	NA	LT, 14.8% vs 19.9%; HET, 7.4% vs 9.6%
Choi et al,^49^ 2022	15	16	RO	Age: 62.3 vs 59.4	T1-4N1-3M0 (T3: 73% vs 44%)	Middle and lower: 73% vs 69%;	SCC	PBT vs PRT; Median PTV: 40.4 Gy/23 F; Neoadjuvant CCRT (platinum + paclitaxel or capecitabine) + surgery (93% vs 88%)	17	OS: 1 y, 86% vs 94%; 2 y, 69% vs 68%; 3 y, 69% vs 58%; pCR: 4 vs 5	Acute: G≥4 lymphocytopenia, 12.5% vs 20%

Abbreviations: AC, adenocarcinoma; ALC, absolute lymphocyte count; CCRT, concurrent chemoradiotherapy; CRT, chemoradiotherapy; CT, computed tomography; D, dimensional; F, fraction; FU, fluoropyrimidine; G, grade; HET, heart toxic effects; HT, hematological toxic effects; IMPT, intensity modulate proton therapy; LT, lung toxic effects; NA, not available or not applicable; OS, overall survival; PBT, proton beam therapy; PCE, pericardial effusion; pCR, pathologic complete response; PE, pleural effusion; PFS, progression-free survival; PO, prospective; PRT, photon radiotherapy; PSPT, passive scattering proton therapy; PTV, planning target volume; RCT, randomized clinical trial; RE, radiation esophagitis; RO, retrospective; RP, radiation pneumonitis; RT, radiotherapy; SCC, squamous cell carcinoma.

^a^ Statistically significant at *P* < .05.

^b^ Data obtained from graphic.

^c^ Propensity-matched analysis.

**Table 2.** Evaluation of Efficacy and Safety of Proton Therapy for Esophageal Cancer

Reference	Patients	Study type	Characteristics				Treatment Strategies	Follow-up, median, mo	Prognosis	Toxic effects
			Age, median, y	Stage, patients, No.	Thoracic site, patients, No.	Pathological type				
Echeverria et al,^4^ 2013	100	RO	66	I, 3; II, 30; III, 51; IV, 7	Upper, 2; middle, 16; lower, 82	AC, 83%	Median PTV: 50.4 Gy/28 F; Radical CCPT	NA	NA	Acute: RP: G≥2, 27; G≥3, 7; G≥4, 0
Hirano et al,^16^ 2018	27 (37)	RO	70	III (100%)	Upper, 5; middle, 9; lower, 13	SCC	PTV: 60 Gy/30 F; Radical CCPT: 89%; 5-FU or cisplatin/nedaplatin	14.8	1-y OS: 91%; 1-y PFS: 41%	Acute: RP: 0; PCE: G≥2, 1; G≥3, 0; RE: G≥2, NA; grade ≥3, 1; Late: RP: 0; PCE: G≥2, 4; G≥3, 0
Parzen et al,^39^ 2021	155	PO	68.2	I-IV (III-IV, 54%)	NA	AC, 77%	IMPT/PSPT; Median PTV: 50.5 Gy; Radical CCPT, 88%; 71%: carboplatin/paclitaxel	NA	NA	Acute: LT: G≥3, NA; G≥3, NA, G≥4, 0; HET: G≥2, NA; G≥3, 0; RE: G≥3, NA, G≥3, 6; G≥4, NA; HT: G≥2, NA; G≥3, 1; G≥4, NA; Lymphocytopenia: G≥4, 0
Ogawa et al,^40^ 2021	60 (103)	RO	69	I, 10; II, 19; III, 31	Upper, 15; middle, 31; lower, 14	SCC	PTV: 60-70 Gy/30-35 F; Radical CCPT: 5-FU + cisplatin + salvage therapy (50%)	51.5	OS: 1 y, 85%; 2 y, 65%; 3 y, 58%; 5 y, 52%^a^ PFS: 1 y, 65%; 2 y, 54%; 3 y, 45%; 5 y, 42%^a^; CR, 45	Acute: HT: G≥2, NA; G≥3, 17; G≥4, NA; PCE: G≥3, 0 ; RP: G≥2, 0; Late: PE: G≥2, NA; G≥3, 1 G≥4, NA; PCE: G≥2, 0; RP: G≥2, 0
Sato et al,^41^ 2020	44 (46)	RO	70	I, 24; II, 15; III, 2; IV, 3	Upper, 8; middle, 26; lower, 10	SCC	PTV: 60 Gy/30 F; Radical CCPT: 5-FU or 5-FU + cisplatin/ nedaplatin + salvage surgery (5)	31	OS: 1 y, 97%; 2 y, 95%; 3 y, 95%^a^; CR, 43	Acute: RE: G≥2, NA; G≥3, 1; G≥4, 0; Late: LT/HET, G≥2, NA; G≥3, 0
Prayongrat et al,^42^ 2017	19 (32)	RO	73	I-IV (III-IV 52.6%)	Upper, 3; middle, 4; lower, 12	AC, 63.2%	IMPT; Median PTV: 50.4 Gy/28 F; Radical CCPT, 74%: docataxel + 5-FU or capecitabine; + surgery (4)	17	OS: 1 y, 100%; 2 y, 87.5% (estimate); 2-y PFS: 50.6% (estimate); CR (16)	Acute: RE: G≥3, 12; G≥3, 3; G≥4, 0; HT: G≥2, 7; G≥3, 2; G≥4, 1; Late: LT: G≥2, 1; G≥3, 1; G≥4, 0 HET: G≥2, 2; G≥3, 0; RP: G≥2, 0; PCE: G≥2, 1; G≥3, 0; PE: G≥2, 1; G≥3, 1; G≥4, 0
Zeng et al,^43^ 2016	13	PO	70	T_3-4_N_0-2_M_0_	Lower, 11	AC, 85%	SFUD; PTV: 50.4 Gy/28F; neoadjuvant CCPT (carboplatin + paclitaxel) + surgery (12)	11	OS/PFS not reached; pCR, 3	Acute: RE: G≥2, 4; G≥3, 1; G≥4, 0; RP: G≥2, 0; HT: G≥2, 6; G≥3, 1; G≥4, 0
Ishikawa et al,^44^ 2015	40	RO	69	I, 16; II, 9; III, 15	Upper, 12; middle, 21; lower, 7	NA	NA; PTV: 60 Gy/30 F; Radical CCPT (62.5%: cisplatin +5-FU)	24	OS: 1 y, 92%; 2 y, 75%; 3 y, 70%; 5 y, 70%^a^ CR, 30; PR, 8	Acute: HT: G≥2, 33; G≥3, 10; G≥4, 2; RE: G≥2, 30; G≥3, 9; G≥4, NA; Late: LT: G≥2, 1; G≥3, 0; PCE: G≥2, 3; G≥3, 0; RE: G≥2, NA, G≥3, 1; G≥4, NA
Lin et al,^45^ 2012	62	PO	68	I-IV (III-IV: 65%)	Upper, 3; middle, 11; lower, 48	AC, 75.8%	PSPT; Median PTV: 50.4 Gy/28 F; Neoadjuvant and radical CCPT (NA) + surgery (29)	20.1	OS (surgery vs no surgery): 1 y, 93% vs 84%; 2 y, 62% vs 60%; 3 y, 41% vs 54% Surgery: pCR, 28%; pPR, 22%	RE: G≥2, 29; G≥3, 6; G≥4, NA; RP: G≥2, 3; G≥3, 2; G≥4, 1
Rutenburg et al,^53^ 2023	17	RO	67.4	T_2-4_N_0-3_M_0_	Upper, 1; middle, 6; lower, 10	AC, 88%	NA; Median PTV: 50.4 Gy/28 F; Neoadjuvant and radical CCPT (76%: carboplatin + paclitaxil) + surgery (9)	2.1	OS: 1 y, 88%; 2 y, 69%; 3 y, 55%^a^	Acute: RE: G≥2, 9; G≥3, 4; G≥4, 0; HT: G≥2, 16; G≥3, 5; G≥4, 2

Abbreviations: 5-FU, fluorouracil; AC, adenocarcinoma; CCPT, concurrent chemoproton therapy; CR, complete response; F, fraction; G, grade; HET, heart toxic effects; HT, hematological toxic effects; IMPT, intensity-modulated proton therapy; LT, lung toxic effects; OS, overall survival; PCE, pericardial effusion; pCR, pathologic complete response; PFS, progression-free survival; PO, prospectively; pPR, pathologic partial response rate; PR, partial response; PSPT, passive scattering proton therapy; PTV, planning target volume; RE, radiation esophagitis; RO, retrospective; RP, radiation pneumonitis; SCC, squamous cell carcinoma; SFUD, single-field uniform dose.

^a^ Obtained from picture.

### Dosimetric Comparison Analysis

We found 25 articles^10,11,12,13,14,15,16,19,20,21,22,23,24,25,28,31,35,37,38,46,47,49,50,51,52^ assessing the lungs for OARS dosimetric parameters and 26 studies^10,11,12,13,14,15,16,17,19,20,21,22,24,25,28,29,31,34,35,37,38,46,47,49,51,52^ assessing the heart for dosimetric parameters, but some of them had no sufficient data for analysis. Pooled analysis found that proton therapy was associated with significantly reduced the mean dose to the lungs: compared with photon therapy, 24.8% less lung volume received 5 Gy or more, 12.9% less lung volume received 10 Gy or more, 6.5% less lung volume received 20 Gy or more, and 3.1% less lung volume received 30 Gy or more, with an overall mean dose reduction of 4.4 Gy. Pooled analysis showed that the proton therapy was associated with significantly reduced the mean dose to the heart: compared with photon therapy, 45.1% less heart volume received 5 Gy or more, 39.5% less heart volume received 10 Gy or more, 26.6% less heart volume received 20 Gy or more, 13.9% less heart volume received 30 Gy or more, and 7.4% less heart volume received 40 Gy or more, with an overall mean dose reduction of 9.5 Gy. In pooled analysis of cardiac substructures, results suggested that the proton therapy was associated with significantly reduced the mean doses to the left ventricle (by 10.2 Gy),^13,17,20^ and left anterior descending artery (by 11.2 Gy).^13,17,20^. Three studies^11,18,21^ described the bone marrow irradiation dose, although there was inconsistency in the definition of bone marrow. Pooled analysis found that proton therapy was associated with significantly reduced mean dose to the bone marrow: 10.2% less bone marrow volume received 10 Gy or more and 10.3% less bone marrow volume received 20 Gy or more, with a mean dose reduction of 2.9 Gy. Proton therapy was associated with significantly reduced the maximum dose to the SC, with a mean reduction of 6.9 Gy.^11,12,14,16,20,21,22,23,24,28,51,52^ All pooled analysis results are presented in eFigure 1 and eTable 6 in Supplement 1.

Most of the included studies did not show significant compromise in planning quality. Sensitivity analysis tests did not detect any changed results, indicating that the results were reliable. Publication bias tests were performed and found a publication bias analyses of volume of heart tissue receiving 30 or more Gy^13,14,16,17,20,21,29,31,35,51,52^ and 40 or more Gy,^14,15,16,17,20,21,22,35,51,52^ but there was no significant difference observed after the trim-and-fill method (eFigure 2 and eTable 6 in Supplement 1).

### Efficacy and Safety of Proton vs Photon Therapy

**Figure 2.**
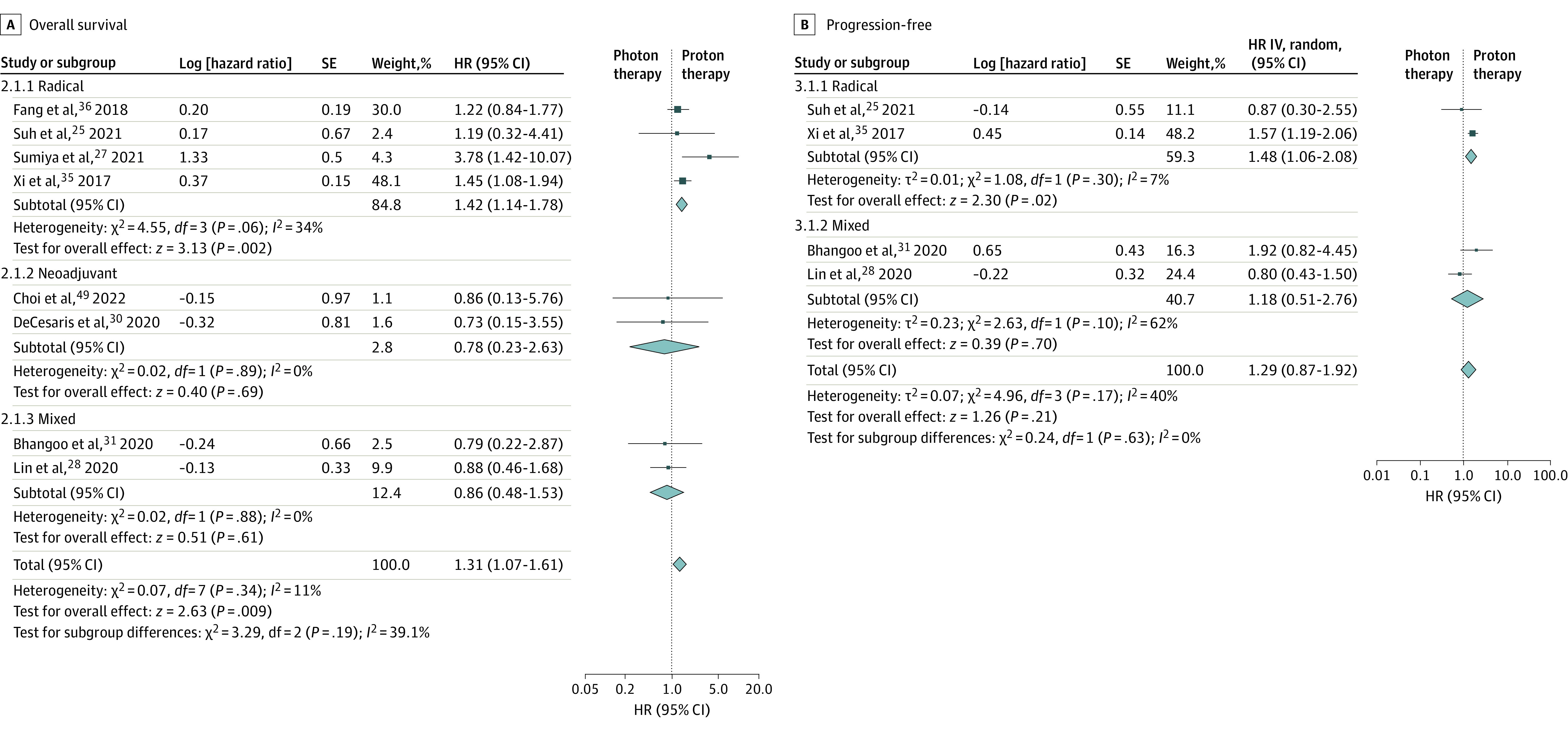
Pooled Analysis of the Overall Survival and Progression-Free Survival in Photon Therapy vs Proton Therapy

We found 8 studies^25,27,28,30,31,35,36,49^ that reported OS (including 510 patients receiving photon therapy and 455 patients receiving proton therapy) and 4 studies that reported PFS^25,28,31,35^ (including 333 patients receiving photon therapy and 258 patients receiving proton therapy). Four studies^25,27,35,36^ used radical therapy, 2 studies^30,49^ used neoadjuvant therapy, and 2 studies^28,31^ used mixed therapies. Pooled analysis of OS found worse OS for photon vs proton therapy for the radical group (HR, 1.42; 95% CI, 1.14-1.78, *I*^2^ = 34%), no significant difference for the neoadjuvant group (HR, 0.78; 95% CI, 0.23-2.63; *I*^2^ = 0%), and no significant difference for the mixed therapy group (HR, 0.86; 95% CI, 0.48-1.53; *I*^2^ = 0%). Overall OS was improved in the proton group compared with the photon group (HR, 1.31; 95% CI, 1.07-1.61; *I*^2^ = 11%) (Figure 2A).^25,27,28,30,31,35,36,49^ Pooled analysis of PFS in the photon vs proton group found worse PFS for the radical therapy group (HR, 1.48; 95% CI, 1.06-2.08; *I*^2^ = 7%) but no significant difference in the mixed therapy group (HR, 1.18; 95% CI, 0.51-2.76; *I*^2^ = 62%) or overall (HR, 1.29; 95% CI, 0.87-1.92; *I*^2^ = 40%) (Figure 2B).^25,28,31,35^ For ORR, only 5 studies^28,30,31,34,49^ reported pathological complete response (pCR), and pooled analysis found no significant difference (odds ratio [OR], 0.90; 95% CI, 0.49-1.69; *I*^2^ = 0) between proton vs photon therapy (eFigure 3 in Supplement 1). The sensitivity analysis found that the overall results changed for OS when the study by Xi et al^35^ was removed (HR, 1.20; 95% CI, 0.91-1.60) and for PFS when study by Lin et al^28^ was removed (HR, 1.55; 95% CI, 1.20-1.99).

Some included studies did not distinguish toxic effects by grade or by acute vs late toxic effects, and the overall cardiopulmonary toxic effects descriptions were inconsistent and incomplete. Thus, only the commonly described toxic effects, such as RP, pleural effusion (PE), pericardial effusion (PCE), radiation esophagitis (RE), and lymphocytopenia were included in the meta-analysis. Pooled analysis of proton vs photon therapy found significant differences in odds of grade 2 or higher RP (OR, 0.40; 95% CI, 0.17-0.97; *I*^2^ = 0%), PE (OR, 0.73; 95% CI, 0.32-1.65; *I*^2^ = 35%), or PCE (OR, 0.20; 95% CI, 0.04-0.96; *I*^2^ = 44%); results were similar for grade 3 or higher RP (OR, 0.45; 95% CI, 0.12-1.67; *I*^2^ = 0%), PE (OR, 0.28; 95% CI, 0.06-1.32; *I*^2^ = 35%), and PCE (OR, 0.34; 95% CI, 0.06-2.06; *I*^2^ = 0%) .^25,27,28,35,38^ Pooled analysis of RE showed no significant difference between proton and photon therapy in odds of grade 2 or higher RE (OR, 1.11; 95% CI, 0.76-1.63; *I*^2^ = 0%) or for grade 3 or higher RE (OR, 0.84; 95% CI, 0.48-1.46; *I*^2^ = 0%).^28,30,31,35^ Pooled analysis found that proton therapy was associated with significantly lower odds of grade 4 or higher lymphocytopenia compared with photon therapy (OR, 0.35; 95% CI, 0.28-0.44; *I*^2^ = 0%).^28,31,32,33,36,46,49^ In contrast, the incidence of other grade 4 or higher toxic effects was too small for pooled analyses to be meaningful. Pooled analyses results are presented in eFigure 4 and eTable 7 in Supplement 1. Sensitivity analysis found that the results of grade 2 or higher RP and grade 2 or higher PCE changed after the elimination 1 of the studies, while no significant changes were observed in other toxic effects.

### Efficacy and Safety of Proton Therapy

In analyses restricted to proton therapy, we found 15 studies^16,25,27,28,30,31,35,36,40,41,42,44,45,49,53^ that reported 1-year OS, 13 studies^25,27,28,30,31,35,36,40,41,44,45,49,53^ that reported 2-year OS, 11 studies^25,27,28,35,36,40,41,44,45,49,53^ that reported 3-year OS, and 7 studies^25,27,28,35,36,40,44^ that reported 5-year OS. Pooled analysis found that that the 1-year OS was 89% (95% CI, 84%-93%; *I*^2^ = 69.3%), 2-year OS was 71% (95% CI, 63%-78%; *I*^2^ = 75.5%), 3-year OS was 63% (95% CI, 53%-73%; *I*^2^ = 81.9%), and 5-year OS was 56% (95% CI, 46%-67%; *I*^2^ = 82%). In analyses of PFS for proton therapy, we found 6 studies^16,25,28,31,35,40^ that reported 1-year PFS, 5 studies,^25,28,31,35,40^ that reported 2-year PFS, 4 studies^25,28,35,40^ that reported 3-year PFS, and 4 studies^25,28,35,40^ that reported 5-year PFS. Pooled analysis found that the 1-year PFS was 65% (95% CI, 53%-77%; *I*^2^ = 83.3%), 2-year PFS was 56% (95% CI, 48%-64%; *I*^2^ = 52.1%), 3-year PFS was 48% (95% CI, 40%-56%; *I*^2^ = 45%), and 5-year PFS was 42% (95% CI, 34%-51%; *I*^2^ = 54.2%). Subgroups analysis by different treatment modalities (ie, neoadjuvant, radical, or mixed) and stage (ie, III-IV<50% or ≥50%) exhibited considerable differences in OS (eFigure 5 in Supplement 1). There may have been significant differences in PFS as well, but there were not enough data for analyses. For ORR, only 4 studies^40,41,42,44^ reported CR and 7 studies^28,30,31,34,43,45,49^ reported pCR. Pooled analysis showed that the CR was 84% (95% CI, 69%-95%; *I*^2^ = 80%) (eFigure 6 in Supplement 1) and the pCR was 31% (95% CI, 18%-44%; *I*^2^ = 56.8%) (eFigure 7 in Supplement 1). All pooled analysis results are presented in Table 3. No publication bias was detected by funnel plot analysis.

**Table 3.** Single-Rate Pooled Analysis of OS and PFS for Proton Therapy

Follow-up, y	Treatment modality					Stage					Overall		Publication bias
	Neoadjuvant, HR (95% CI)	Radical		Mixed^a^		III-IV (<50%)		III-IV (≥50%)					
		HR (95% CI)	*I*^2^, %	HR (95% CI)	*I*^2^, %	HR (95% CI)	*I*^2^, %	HR (95% CI)	*I*^2^, %	HR (95% CI)		*I*^2^, %	
**OS**													
1	0.82 (0.66-0.94)^b^	0.92 (0.85-0.97)	78.1	0.84 (0.77-0.90)	8.4	0.96 (0.88-1.00)	72.1	0.85 (0.80-0.90)	45.4	0.89 (0.84-0.93)		69.3	NA^c^
2	0.70 (0.52-0.85)^b^	0.77 (0.67-0.85)	79.7	0.58 (0.47-0.68)	42.6	0.85 (0.74-0.93)	68.8	0.64 (0.59-0.68)	6.6	0.71 (0.63-0.78)		75.5	NA^c^
3	NA^d^	0.68 (0.55-0.80)	87.1	0.50 (0.41-0.59)^b^		0.78 (0.63-0.91)	80.8	0.53 (0.48-0.58)	0	0.63 (0.53-0.73)		81.9	NA^c^
5	NA^e^	0.58 (0.46-0.70)	84.6	NA^d^		0.71 (0.64-0.79)	0	0.45 (0.39-0.50)	0	0.56 (0.46-0.67)		82	None^f^
**PFS**													
1	NA^e^	0.65 (0.48-0.82)	88.9	0.64 (0.49-0.79)	50.5	NA^d^		0.61 (0.53-0.68)	45.4	0.65 (0.53-0.77)		83.3	None^f^
2	NA^e^	0.57 (0.45-0.70)	73.8	0.54 (0.43-0.65)	0	NA^d^		0.52 (0.46-0.57)	0	0.56 (0.48-0.64)		52.1	None^f^
3	NA^e^	0.49 (0.38-0.60)^b^		NA^d^	NA	NA^d^		0.44 (0.38-0.51)	85.4	0.48 (0.40-0.56)		45.0	NA^c^
5	NA^e^	0.43 (0.31-0.55)^b^		NA^d^	NA	NA^d^		0.38 (0.32-0.44)^b^		0.42 (0.34-0.51)		54.2	NA^c^

Abbreviations: NA, not applicable or not evaluable; OS, overall survival; PFS, progression-free survival.

^a^ Mixed indicates both neoadjuvant and radical treatments were used.

^b^ Included 3 studies or fewer (after double-arcsine transformations), so the *I*^2^ is not shown.

^c^ After double-arcsine transformations (did not perform publication bias test).

^d^ Represents only 1 study.

^e^ Represents no study.

^f^ Represents publication bias detected by funnel plot (because included <10 studies).

Eight studies reported grade 2 or higher RE,^30,31,35,42,43,44,45,53^ 10 studies reported grade 2 or higher RP,^4,16,27,28,35,38,40,42,43,45^ 4 studies reported grade 2 or higher PE,^27,35,38,42^ and 7 studies^16,27,35,38,42,44^ reported grade 2 or higher PCE for proton therapy. Pooled analysis showed that the grade 2 or higher incidence of RE was 50% (95% CI, 40%-59%; *I*^2^ = 60.8%), RP was 2% (95% CI, 0%-6%; *I*^2^ = 83.4%), PE was 4% (95% CI, 2%-7%; *I*^2^ = 0%), and PCE was 3% (95% CI, 0%-7%; *I*^2^ = 62.7%) in the proton therapy group. The included studies reporting grade 3 or higher and grade 4 or higher toxic effects occurred rarely; thus, we did not perform a pooled analysis for these, except for grade 3 or higher RE^16,28,31,35,39,41,42,43,44,45,53^ (incidence, 8%; 95% CI, 5%-12%; *I*^2^ = 57.1%) and grade 4 or higher lymphopenia^28,31,32,33,36,39,46,49^ (incidence, 17%; 95% CI, 7%-30%; *I*^2^ = 93.8%) in proton therapy (eTable 8 in Supplement 1).

## Discussion

This meta-analysis is the first study to our knowledge to evaluate the efficacy and safety of proton therapy vs photon therapy for esophageal cancer; we also assessed proton therapy separately. Currently, radiotherapy for esophageal cancer remains based on photon therapy, while advanced proton therapy offers higher target conformability and low doses to OARs. Furthermore, the difference in dosimetric benefit between proton therapy and different photon techniques (eg, IMRT, volumetric modulated arc therapy) and whether the benefit translates into clinical benefit are unknown. Previous studies enrolled the patients who underwent both proton and photon therapy or did not perform a separate analysis of proton therapy.^54,55,56^

For the dosimetric parameters, the meta-analysis showed that proton therapy was associated with significantly reduced irradiation doses in the lungs, heart, SC, and bone marrow. Prior studies have shown that coronary and ventricular irradiation doses were associated with coronary artery disease and chronic heart failure.^13,57^ We performed a pooled analysis of the cardiac substructure, and we found that the mean doses to the left ventricle and left anterior descending artery were also significantly decreased. Although the bone marrow was defined inconsistently between studies, such as the thoracic vertebral body (from T1-T12 or C2-L1) or containing the ribs and sternum.^11,18,27^ However, no clear criteria are available. In addition, we did not distinguish proton techniques, and studies have shown that intensity-modulated proton therapy has higher target-area conformability and lower cardiopulmonary radiation dose than passive-scattering proton therapy.^17,58^ Gjyshi et al^59^ demonstrated that intensity-modulated proton therapy significantly reduced grade 3 or higher cardiopulmonary toxic effects in patients with NSCLC compared with passive-scattering proton therapy.

Meta-analysis showed that photon therapy was associated with poor OS compared with proton therapy, and the subgroup analysis showed similar results in the radical group, but these results were not observed in the mixed group. Analogously, the subgroup meta-analysis showed that photon therapy, compared with proton therapy, also was associated with poor PFS in the radical group, but no significant differences were observed in the mixed group or overall. Sensitivity analysis suggest that we need to be cautious about these results, which may be related to sample size, follow-up time, patient heterogeneity, chemotherapy regimens, and treatment modalities (radical or neoadjuvant). A 2022 meta-analysis by Zhu et al^55^ did not find any survival benefit from proton therapy, which may be related to the incomplete included studies and lack of subgroup analysis. Subgroup analysis revealed considerable differences in OS between different stage (III-IV<50% or ≥50%) and treatment modalities (neoadjuvant, radical, or mixed), while differences in PFS were not observed. Subgroup analysis found lower OS in the mixed group (neoadjuvant and radical therapy), which may be related to patient heterogeneity (eg, number of patients, stage, pathological type, tumor location, prescription dose), surgical regimens, timing, and whether salvage surgery was performed. Overall, both acute and late toxic effects were relatively low, except for grade 2 or higher RE, grade 2 or higher PCE, and grade 4 or higher lymphocytopenia. Carbon ion therapy has a similar dosimetric distribution with proton therapy and a higher relative biological effectiveness, but there is a paucity of data for esophageal cancer.^56^

This meta-analysis also found that proton therapy was associated with significantly decreased the overall incidence of grade 2 or higher RP and grade 2 or higher PCE compared with photon therapy (regardless of acute or late onset). Proton therapy was also associated with significantly reduced the incidence of acute grade 4 or higher lymphocytopenia. A 2019 study by Xu et al^60^ found that cardiopulmonary doses are risk factors for radiation-induced toxic effects and are independent risk factors for poor OS. RP is a common and severe toxic effect after radiotherapy in esophageal cancer. According to research results, there is heterogeneity in the distribution of lung function and inconsistent sensitivity to radiation.^61^ A study by Vinogradskiy et al^62^ showed that 4-dimensional computed tomography–based ventilation functional lung imaging-guided radiotherapy significantly reduced the incidence of grade 2 or higher RP. A study by Zhou et al^63^ found that using functional lung protection in esophageal cancer radiotherapy planning was associated with decreasing functional lung mean dose by 1.5 Gy and volume of lung tissue receiving 20 Gy of more by 4.7%, without compromising planning quality. A study by Ieko et al^64^ integrated functional lung protection into planning and showed that the proton-based functional lung–sparing planning reduced the functional lung dose more than IMRT-based functional lung–sparing planning. Previously, the low survival rate of patients with esophageal cancer and the fact that cardiotoxic effects were considered late toxic effects meant that toxic effects were not emphasized, but relatively high rates of death related to cardiotoxic effects have been reported in the long-term follow-up in patients with breast cancer and Hodgkin lymphoma.^65^ In recent years, various combinations have improved the prognosis of patients with esophageal cancer. Furthermore, the heart is particularly exposed to high doses, due to its anatomical location adjacent to the esophagus and the high prescription dose. Previous studies have demonstrated that older patients, lower esophageal subsite, and pre-existing cardiac disease are associated with death related to cardiotoxic effects after radiotherapy.^29,66^ Pericardial effusion is the most frequently observed cardiotoxic effect after radiotherapy, and it is significantly associated with the irradiated dose to the pericardium.^65^ However, there were insufficient data in the included studies for pooled analysis.

Previous studies have shown that bone marrow radiation dose is associated with hematologic toxic effects, especially in red bone marrow, which is highly sensitive to radiation. A meta-analysis by Zhou et al^67^ reported that pelvic bone marrow protection planning significantly reduced the incidence of grade 2 or higher hematologic toxic effects in cervical cancer. A 2016 study by Deek et al^68^ found that thoracic vertebral body dose was associated with hematological toxic effects during radiotherapy in patients with NSCLC. Other studies have shown that cardiac, aortic, spleen, and body doses are also associated with decreased grade 4 or higher lymphocytopenia, presumably associated with the blood (regarded as a moving OAR) circulating lymphocytes in it.^69,70^ A study by Davuluri et al^71^ reported that grade 4 or higher lymphocytopenia was significantly associated with OS in patients with esophageal cancer. Lymphocytopenia may be associated with an increased risk of infection and blunted antitumor immune cell response.^36^ In the future, we need to pay more attention to reducing the incidence of grade 4 or higher lymphocytopenia, especially when radiotherapy is combined with immunotherapy.

### Limitations

There were several limitations in this study. First, there was significant heterogeneity in the OARs dosimetric analysis. The sources of heterogeneity might be patient characteristics (eg, location, planning target volume), radiotherapy modalities, and OAR dose limitations. Second, the small number of patients included in the study may be another limitation. Third, the data descriptions of the included studies were not detailed. For example, mean and SD were unavailable for dosimetric analysis, HR and 95% CI were unavailable for prognosis analysis, and toxic effects analysis did not distinguish grade and categories. The values extracted from the graphs using software might differ slightly from the actual data. Fourth, we did not pay attention to other prognosis metrics (eg, local control rate, distant metastasis–free survival), other toxic effects (eg, vomiting, fatigue), and postoperative complications. Moreover, there were not enough clinical RCTs to evaluate the efficacy and safety of proton and photon therapy.

## Conclusions

This meta-analysis indicated that proton therapy was associated with significantly reduced the OARs irradiation dose, and there was a considerable difference between different photon techniques. In addition, proton therapy was associated with significantly improved prognosis and toxic effects in patients with esophageal cancer, but caution is warranted. In the future, it is still necessary to verify the benefits of proton therapy vs photon therapy for esophageal cancer in RCTs.

## Notes

Supplement 1. **eAppendix.** Literature Search Strategy **eFigure 1.** Forest Plots of Dose-Volume Parameters for OARs With Proton vs Photon Therapy **eFigure 2.** Funnel Plot of Dose-Volume Parameters for OARs With Proton vs Photon Therapy (≥10 Articles) **eFigure 3.** Pooled Analysis of pCR (Proton vs Photon Therapy) **eFigure 4.** Forest Plots of the Incidence of Toxic Effects in the Proton and Photon Therapy Groups (Described as OR) **eFigure 5.** Example of Single-Rate Pooled Analysis of OS and PFS for Proton Therapy **eFigure 6.** Single-Rate Pooled Analysis of CR (Proton Therapy) **eFigure 7.** Single-Rate Pooled Analysis of pCR (Proton Therapy) **eTable 1.** Detailed Comparison Information on OARs Dosimetric and Planning Quality From Proton and Photon Radiotherapy Planning **eTable 2.** Results of Quality Assessment Using the Newcastle-Ottawa Scale for Non-RCT Studies (Dosimetric Parameters) **eTable 3.** Results of Quality Assessment Using the Newcastle-Ottawa Scale for Non-RCT Studies (Efficacy and Safety of Proton vs Photon Therapy) **eTable 4.** Results of Quality Assessment Using the Cochrane Collaboration’s Tool (RCT) **eTable 5.** Results of Quality Assessment Using the MINORS (Single-Group Tests) **eTable 6.** Pooled Analysis of Dose-Volume Parameters for OARs with Proton vs Photon Therapy **eTable 7.** Pooled Analysis of the Incidence of Toxic Effects in the Proton and Photon Therapy Group (Described as OR) **eTable 8.** Single-Rate Pooled Analysis for the Incidence of Toxic Effects in Proton Therapy

Supplement 2.
Data Sharing Statement
