# Supplementary material for: Efficacy and Safety in Proton Therapy and Photon Therapy for Patients With Esophageal Cancer: A Meta-Analysis
Source: JAMA Netw Open. 2023 Aug 15;6(8):e2328136. doi: 10.1001/jamanetworkopen.2023.28136 (PMC10427943; doi:10.1001/jamanetworkopen.2023.28136)
Supplement: Supplement 1. — eAppendix. Literature Search Strategy eFigure 1. Forest Plots of Dose-Volume Parameters for OARs With Proton vs Photon Therapy eFigure 2. Funnel Plot of Dose-Volume Parameters for OARs With Proton vs Photon Therapy (≥10 Articles) eFigure 3. Pooled Analysis of pCR (Proton vs Photon Therapy) eFigure 4. Forest Plots of the Incidence of Toxic Effects in the Proton and Photon Therapy Groups (Described as OR) eFigure 5. Example of Single-Rate Pooled Analysis of OS and PFS for Proton Therapy eFigure 6. Single-Rate Pooled Analysis of CR (Proton Therapy) eFigure 7. Single-Rate Pooled Analysis of pCR (Proton Therapy) eTable 1. Detailed Comparison Information on OARs Dosimetric and Planning Quality From Proton and Photon Radiotherapy Planning eTable 2. Results of Quality Assessment Using the Newcastle-Ottawa Scale for Non-RCT Studies (Dosimetric Parameters) eTable 3. Results of Quality Assessment Using the Newcastle-Ottawa Scale for Non-RCT Studies (Efficacy and Safety of Proton vs Photon Therapy) eTable 4. Results of Quality Assessment Using the Cochrane Collaboration’s Tool (RCT) eTable 5. Results of Quality Assessment Using the MINORS (Single-Group Tests) eTable 6. Pooled Analysis of Dose-Volume Parameters for OARs with Proton vs Photon Therapy eTable 7. Pooled Analysis of the Incidence of Toxic Effects in the Proton and Photon Therapy Group (Described as OR) eTable 8. Single-Rate Pooled Analysis for the Incidence of Toxic Effects in Proton Therapy [file jamanetwopen-e2328136-s001.pdf]

Supplemental Online Content

Zhou P, Du Y, Zhang Y, et al. Efficacy and safety in proton therapy and photon therapy for patients with esophageal cancer: a meta-analysis. *JAMA Netw Open*. 2023;6(8):e2328136. doi:10.1001/jamanetworkopen.2023.28136

eAppendix. Literature Search Strategy
eFigure 1. Forest Plots of Dose-Volume Parameters for OARs With Proton vs Photon Therapy
eFigure 2. Funnel Plot of Dose-Volume Parameters for OARs With Proton vs Photon Therapy (≥10 Articles)
eFigure 3. Pooled Analysis of pCR (Proton vs Photon Therapy)
eFigure 4. Forest Plots of the Incidence of Toxic Effects in the Proton and Photon Therapy Groups (Described as OR)
eFigure 5. Example of Single-Rate Pooled Analysis of OS and PFS for Proton Therapy
eFigure 6. Single-Rate Pooled Analysis of CR (Proton Therapy)
eFigure 7. Single-Rate Pooled Analysis of pCR (Proton Therapy)
eTable 1. Detailed Comparison Information on OARs Dosimetric and Planning Quality From Proton and Photon Radiotherapy Planning
eTable 2. Results of Quality Assessment Using the Newcastle-Ottawa Scale for Non-RCT Studies (Dosimetric Parameters)
eTable 3. Results of Quality Assessment Using the Newcastle-Ottawa Scale for Non-RCT Studies (Efficacy and Safety of Proton vs Photon Therapy)
eTable 4. Results of Quality Assessment Using the Cochrane Collaboration's Tool (RCT)
eTable 5. Results of Quality Assessment Using the MINORS (Single-Group Tests)
eTable 6. Pooled Analysis of Dose-Volume Parameters for OARs with Proton vs Photon Therapy
eTable 7. Pooled Analysis of the Incidence of Toxic Effects in the Proton and Photon Therapy Group (Described as OR)
eTable 8. Single-Rate Pooled Analysis for the Incidence of Toxic Effects in Proton Therapy

This supplemental material has been provided by the authors to give readers additional information about their work.

© 2023 Zhou P et al. *JAMA Network Open.*

eAppendix. Literature Search Strategy

PubMed

#1. "Esophageal Neoplasms"[Mesh]

#2. ((((((((((((((((Esophageal Neoplasm) OR (Neoplasm, Esophageal)) OR (Esophagus Neoplasm)) OR (Esophagus Neoplasms)) OR (Neoplasm, Esophagus)) OR (Neoplasms, Esophagus)) OR (Neoplasms, Esophageal)) OR (Cancer of Esophagus)) OR (Cancer of the Esophagus)) OR (Esophagus Cancer)) OR (Cancer, Esophagus)) OR (Cancers, Esophagus)) OR (Esophagus Cancers)) OR (Esophageal Cancer)) OR (Cancer, Esophageal)) OR (Cancers, Esophageal)) OR (Esophageal Cancers)

#3. #1 OR #2

#4. "Proton Therapy"[Mesh]

#5. (((((((Proton Therapies) OR (Therapies, Proton)) OR (Therapy, Proton)) OR (Proton Beam Therapy)) OR (Proton Beam Therapies)) OR (Therapies, Proton Beam)) OR (Therapy, Proton Beam)) OR (Proton Beam Radiation Therapy)

#6. #4 OR #5

#7. #3 AND #6

Embase

#1. 'esophagus cancer'/exp

#2. 'esophag* neoplasm*' OR 'neoplasm*, esophag*' OR 'cancer of esophag*' OR 'esophag* cancer*' OR 'cancer*, esophag*' OR 'cancer* of the esophag*'

#3. #1 OR #2

#4. 'proton therapy'/exp

#5. 'proton therap*' OR 'therap*, proton' OR 'proton beam therap*' OR 'therap*, proton beam' OR 'proton beam radiation therap*'

#6. #4 OR #5

#7. #3 AND #6

Cochrane

#1. MeSH descriptor: [Esophageal Neoplasms] explode all trees

#2. ('esophag* neoplasm*' OR 'neoplasm*, esophag*' OR 'cancer of esophag*' OR 'esophag* cancer*' OR 'cancer*, esophag*' OR 'cancer* of the esophag*'):ti,ab,kw

#3. #1 OR #2

#4. MeSH descriptor: [Proton Therapy] explode all trees

#5. ('proton therap*' OR 'therap*, proton' OR 'proton beam therap*' OR 'therap*, proton beam' OR 'proton beam radiation therap*'):ti,ab,kw

#6. #4 OR #5

#8. #3 AND #6

© 2023 Zhou P et al. *JAMA Network Open.*

Web of science

#1. TS= esophag*

#2. TS= (cancer* OR tumor* OR neoplasm*)

#3. #1 AND #2

#4. TS= (proton therap*) OR (therap*, proton) OR (proton beam therap*) OR (therap*, proton beam) OR (proton beam radiation therap*)

#5. #3 AND #4

TS=Topic   Search in: Web of Science Core Collection.

Link:

https://www.webofscience.com/wos/woscc/summary/ae4b2cf6-c13f-48e9-949a-0ea1fb714582-6e1f3b8e/relevance/1

Sinomed

#1. "食管肿瘤"[全部字段:智能] OR "食管癌"[全部字段:智能] OR "食道肿瘤"[全部字段:智能] OR "食道癌"[全部字段:智能]

#2. "质子治疗"[全部字段:智能] OR "质子放疗"[全部字段:智能] OR "质子"[全部字段:智能]

#3. #1 AND #2

CNKI

(TKA % '食管肿瘤' OR TKA % '食管癌' OR TKA % '食道肿瘤' OR TKA % '食道癌') AND (TKA % '质子治疗' OR TKA % '质子' OR TKA % '质子放疗')

TKA: 篇关摘; %: 模糊检索

© 2023 Zhou P et al. *JAMA Network Open.*

eFigure 1. Forest Plots of Dose-Volume Parameters for OARs With Proton vs Photon Therapy

a) Lung-D_{mean}

Study or Subgroup	Proton Mean	Proton SD	Proton Total	Photon Mean	Photon SD	Photon Total	Weight	Mean Difference IV, Random, 95% CI
**1.2.1 vs. 3D-CRT**								
Hirano et al. 16 (2018)	5.8	2.2	27	8.2	2.1	27	4.7%	-2.40 [-3.55, -1.25]
Lin et al. 37 (2017)	6.1	2.6	111	10.5	3.9	214	4.9%	-4.40 [-5.11, -3.69]
Ling et al. 20 (2014)	6	2.6	10	9.4	4	10	3.4%	-3.40 [-6.36, -0.44]
**Subtotal (95% CI)**			**148**			**251**	**13.1%**	**-3.46 [-5.03, -1.88]**
Heterogeneity: Tau² = 1.33; Chi² = 8.51, df = 2 (P = 0.01); I² = 77%								
Test for overall effect: Z = 4.31 (P < 0.0001)								
**1.2.2 vs. IMRT**								
Bhangoo et al. 31 (2020)	4.1	1.5	32	8.4	6.2	32	4.0%	-4.30 [-6.51, -2.09]
Cui et al. 52 (2022)	4.2	1.9	30	12.6	3.8	30	4.5%	-8.40 [-9.92, -6.88]
Hirano et al. 16 (2018)	5.8	2.2	27	9.5	2.2	27	4.7%	-3.70 [-4.87, -2.53]
Lin et al. 28 (2020)	4.9	1.9	46	8.9	3.6	61	4.8%	-4.00 [-5.06, -2.94]
Lin et al. 37 (2017)	6.1	2.6	111	9.5	3.2	255	5.0%	-3.40 [-4.02, -2.78]
Ling et al. 20 (2014)	6	2.6	10	9.5	3.2	10	3.7%	-3.50 [-6.06, -0.94]
Mu et al. 23 (2011)	7.3	1.8	10	12.1	1.8	10	4.5%	-4.80 [-6.38, -3.22]
Møller et al. 15 (2019)	3.3	1.9	26	8.8	3.8	26	4.4%	-5.50 [-7.13, -3.87]
Welsh et al. 21 (2011)	4.9	2.2	10	8.3	3.2	10	3.8%	-3.40 [-5.81, -0.99]
Xi et al. 35 (2017)	6.5	3.2	132	10	3.3	211	4.9%	-3.50 [-4.20, -2.80]
Zhang et al. 11 (2021)	10.3	6	25	12.3	6.2	25	3.1%	-2.00 [-5.38, 1.38]
Zhang et al. 22 (2008)	6.8	1.4	15	9.8	1.8	15	4.7%	-3.00 [-4.15, -1.85]
Zhang et al. 51 (2022)	3.8	0.7	11	14.1	2.8	11	4.4%	-10.30 [-12.01, -8.59]
**Subtotal (95% CI)**			**485**			**723**	**56.5%**	**-4.66 [-5.70, -3.61]**
Heterogeneity: Tau² = 2.94; Chi² = 98.71, df = 12 (P < 0.00001); I² = 88%								
Test for overall effect: Z = 8.74 (P < 0.00001)								
**1.2.3 vs. VMAT**								
Anakotta et al. 12 (2020)	4.1	1.6	19	9	3.5	19	4.3%	-4.90 [-6.63, -3.17]
Celik et al. 13 (2020)	2.9	1.2	20	8.6	2.9	20	4.6%	-5.70 [-7.08, -4.32]
Cui et al. 52 (2022)	4.2	1.9	30	11.5	3.5	30	4.6%	-7.30 [-8.73, -5.87]
Kato et al. 50 (2022)	2.1	0.7	10	2.2	0.7	10	5.0%	-0.10 [-0.71, 0.51]
Liu et al. 14 (2019)	4.3	2.3	19	8.17	2.9	16	4.3%	-3.87 [-5.63, -2.11]
Warren et al. 19 (2016)	6.7	2.2	21	13.5	2.6	21	4.5%	-6.80 [-8.26, -5.34]
Zhang et al. 11 (2021)	10.3	6	25	12.3	5.9	25	3.2%	-2.00 [-5.30, 1.30]
**Subtotal (95% CI)**			**144**			**141**	**30.5%**	**-4.41 [-7.14, -1.68]**
Heterogeneity: Tau² = 12.76; Chi² = 165.79, df = 6 (P < 0.00001); I² = 96%								
Test for overall effect: Z = 3.17 (P = 0.002)								
**Total (95% CI)**			**777**			**1115**	**100.0%**	**-4.42 [-5.38, -3.47]**
Heterogeneity: Tau² = 4.69; Chi² = 298.78, df = 22 (P < 0.00001); I² = 93%								
Test for overall effect: Z = 9.07 (P < 0.00001)								
Test for subgroup differences: Chi² = 1.57, df = 2 (P = 0.46), I² = 0%								

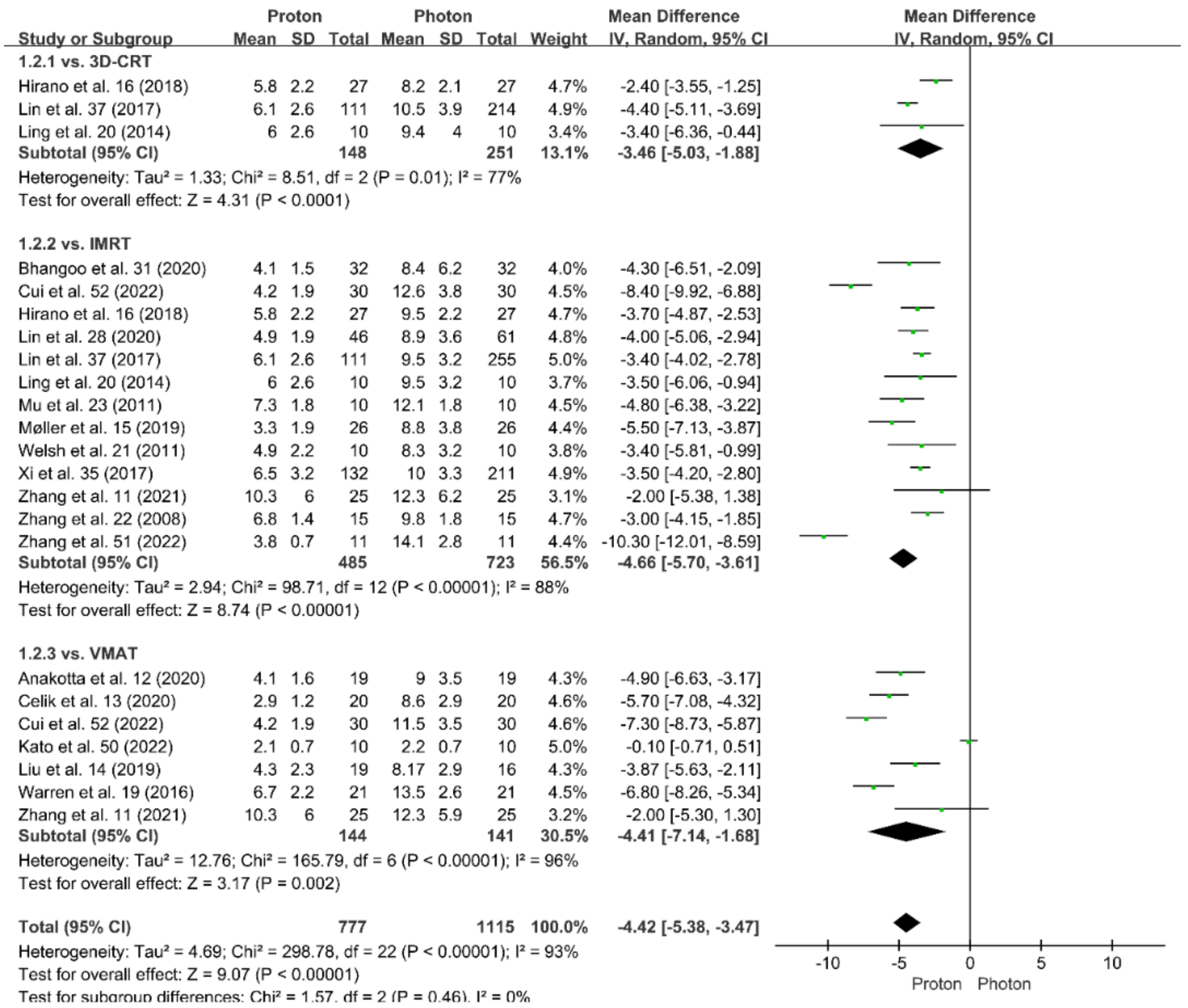

b) Lung-V_5

Study or Subgroup	Proton Mean	Proton SD	Proton Total	Photon Mean	Photon SD	Photon Total	Weight	Mean Difference IV, Random, 95% CI
**1.1.1 vs. 3D-CRT**								
Hirano et al. 16 (2018)	25.2	10.8	27	33.1	8.8	27	5.9%	-7.90 [-13.15, -2.65]
Ling et al. 20 (2014)	21.4	10.3	10	34.1	13.9	10	5.2%	-12.70 [-23.42, -1.98]
**Subtotal (95% CI)**			**37**			**37**	**11.1%**	**-8.83 [-13.55, -4.11]**
Heterogeneity: Tau² = 0.00; Chi² = 0.62, df = 1 (P = 0.43); I² = 0%								
Test for overall effect: Z = 3.67 (P = 0.0002)								
**1.1.2 vs. IMRT**								
Cui et al. 52 (2022)	14.2	5.4	30	55	18.4	30	5.7%	-40.80 [-47.66, -33.94]
Hirano et al. 16 (2018)	25.2	10.8	27	45.2	10.5	27	5.8%	-20.00 [-25.68, -14.32]
Lin et al. 28 (2020)	21.2	8.4	46	44.8	16.9	61	5.9%	-23.60 [-28.49, -18.71]
Ling et al. 20 (2014)	21.4	10.3	10	46.9	17.6	10	5.0%	-25.50 [-38.14, -12.86]
Mu et al. 23 (2011)	19.4	4.3	10	50.8	11	10	5.7%	-31.40 [-38.72, -24.08]
Møller et al. 15 (2019)	10.7	6	26	42.8	15.1	26	5.8%	-32.10 [-38.35, -25.85]
Welsh et al. 21 (2011)	19	6.3	10	38	15.8	10	5.3%	-19.00 [-29.54, -8.46]
Xi et al. 35 (2017)	28.4	14.1	132	48.1	14.7	211	6.0%	-19.70 [-22.82, -16.58]
Zhang et al. 11 (2021)	32.7	18.2	25	50.8	23.7	25	5.1%	-18.10 [-29.81, -6.39]
Zhang et al. 22 (2008)	31.8	5.9	15	49.6	8.9	15	5.9%	-17.80 [-23.20, -12.40]
Zhang et al. 51 (2022)	20.5	3.9	11	71.2	17.9	11	5.2%	-50.70 [-61.53, -39.87]
**Subtotal (95% CI)**			**342**			**436**	**61.4%**	**-26.89 [-32.19, -21.58]**
Heterogeneity: Tau² = 64.53; Chi² = 72.93, df = 10 (P < 0.00001); I² = 86%								
Test for overall effect: Z = 9.93 (P < 0.00001)								
**1.1.3 vs. VMAT**								
Anakotta et al. 12 (2020)	21.1	8.6	19	53.7	20.1	19	5.4%	-32.60 [-42.43, -22.77]
Cui et al. 52 (2022)	14.2	5.4	30	58	17.9	30	5.7%	-43.80 [-50.49, -37.11]
Kato et al. 50 (2022)	9.7	3.5	10	9.8	3.4	10	6.0%	-0.10 [-3.12, 2.92]
Liu et al. 14 (2019)	16.7	8.1	19	47.6	18.9	16	5.4%	-30.90 [-40.85, -20.95]
Zhang et al. 11 (2021)	32.7	18.2	25	55.5	25.3	25	5.0%	-22.80 [-35.02, -10.58]
**Subtotal (95% CI)**			**103**			**100**	**27.5%**	**-25.93 [-47.42, -4.45]**
Heterogeneity: Tau² = 580.25; Chi² = 179.67, df = 4 (P < 0.00001); I² = 98%								
Test for overall effect: Z = 2.37 (P = 0.02)								
**Total (95% CI)**			**482**			**573**	**100.0%**	**-24.81 [-31.40, -18.21]**
Heterogeneity: Tau² = 185.85; Chi² = 339.00, df = 17 (P < 0.00001); I² = 95%								
Test for overall effect: Z = 7.37 (P < 0.00001)								
Test for subgroup differences: Chi² = 25.51, df = 2 (P < 0.00001), I² = 92.2%								

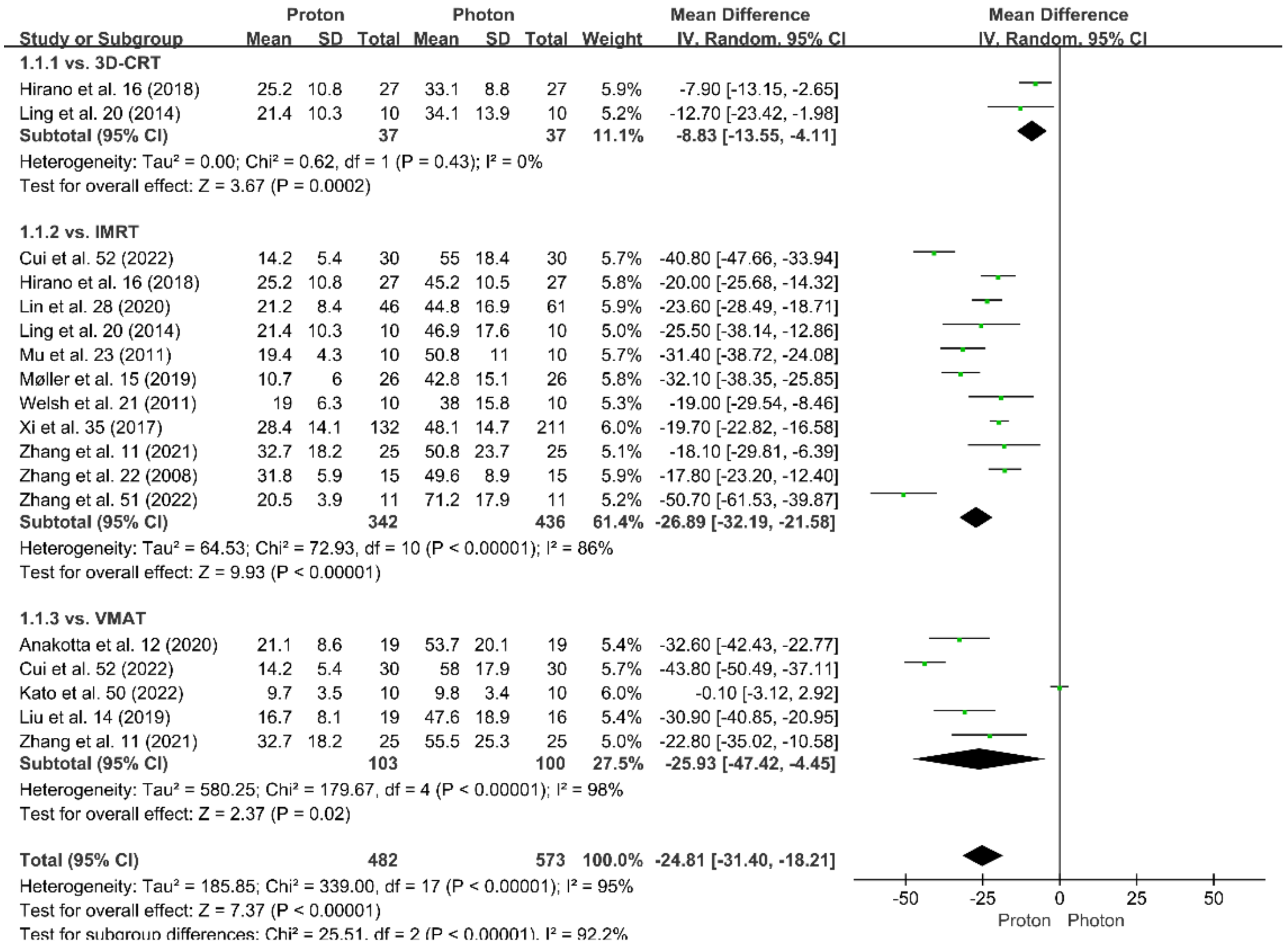

© 2023 Zhou P et al. *JAMA Network Open.*

c) Lung-V_{10}

Study or Subgroup	Proton Mean	Proton SD	Proton Total	Photon Mean	Photon SD	Photon Total	Weight	Mean Difference IV, Random, 95% CI
**1.3.1 vs. 3D-CRT**								
Hirano et al. 16 (2018)	21.3	9.1	27	25.7	7.5	27	9.8%	-4.40 [-8.85, 0.05]
Ling et al. 20 (2014)	19.4	8.6	10	29.1	12.7	10	6.8%	-9.70 [-19.21, -0.19]
**Subtotal (95% CI)**			**37**			**37**	**16.5%**	**-5.35 [-9.38, -1.32]**
Heterogeneity: Tau² = 0.00; Chi² = 0.98, df = 1 (P = 0.32); I² = 0%								
Test for overall effect: Z = 2.60 (P = 0.009)								
**1.3.2 vs. IMRT**								
Cui et al. 52 (2022)	11.7	4.8	30	38.3	14.9	30	9.1%	-26.60 [-32.20, -21.00]
Hirano et al. 16 (2018)	21.3	9.1	27	30	6.8	27	9.9%	-8.70 [-12.98, -4.42]
Ling et al. 20 (2014)	19.4	8.6	10	37.8	14.7	10	6.2%	-18.40 [-28.96, -7.84]
Welsh et al. 21 (2011)	15	6.3	10	23	9.5	10	8.2%	-8.00 [-15.07, -0.93]
Xi et al. 35 (2017)	23.2	11.4	132	32.3	11.3	211	10.7%	-9.10 [-11.57, -6.63]
Zhang et al. 11 (2021)	28.8	16	25	39.8	20.6	25	6.4%	-11.00 [-21.22, -0.78]
Zhang et al. 22 (2008)	23.8	7.1	15	33.9	8.1	15	9.2%	-10.10 [-15.55, -4.65]
**Subtotal (95% CI)**			**249**			**328**	**59.7%**	**-12.87 [-17.91, -7.83]**
Heterogeneity: Tau² = 35.07; Chi² = 35.84, df = 6 (P < 0.00001); I² = 83%								
Test for overall effect: Z = 5.01 (P < 0.00001)								
**1.3.3 vs. VMAT**								
Cui et al. 52 (2022)	11.7	4.8	30	35.6	17.1	30	8.7%	-23.90 [-30.26, -17.54]
Kato et al. 50 (2022)	7	2.6	10	18	8.1	10	9.3%	-11.00 [-16.27, -5.73]
Zhang et al. 11 (2021)	28.8	16	25	47	24.1	25	5.8%	-18.20 [-29.54, -6.86]
**Subtotal (95% CI)**			**65**			**65**	**23.8%**	**-17.51 [-26.57, -8.45]**
Heterogeneity: Tau² = 48.90; Chi² = 9.47, df = 2 (P = 0.009); I² = 79%								
Test for overall effect: Z = 3.79 (P = 0.0002)								
**Total (95% CI)**			**351**			**430**	**100.0%**	**-12.92 [-16.84, -9.00]**
Heterogeneity: Tau² = 35.66; Chi² = 61.73, df = 11 (P < 0.00001); I² = 82%								
Test for overall effect: Z = 6.46 (P < 0.00001)								
Test for subgroup differences: Chi² = 8.77, df = 2 (P = 0.01), I² = 77.2%								

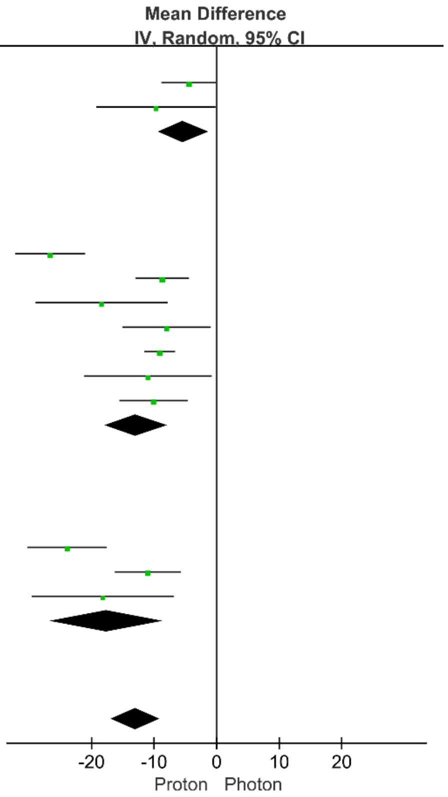

d) Lung-V_{20}

Study or Subgroup	Proton Mean	Proton SD	Proton Total	Photon Mean	Photon SD	Photon Total	Weight	Mean Difference IV, Random, 95% CI
**1.4.1 vs. 3D-CRT**								
Hirano et al. 16 (2018)	11.7	5.2	27	17	5.6	27	5.3%	-5.30 [-8.18, -2.42]
Ling et al. 20 (2014)	15.3	6.5	10	22.1	10.8	10	2.7%	-6.80 [-14.61, 1.01]
**Subtotal (95% CI)**			**37**			**37**	**8.0%**	**-5.48 [-8.18, -2.78]**
Heterogeneity: Tau² = 0.00; Chi² = 0.12, df = 1 (P = 0.72); I² = 0%								
Test for overall effect: Z = 3.97 (P < 0.0001)								
**1.4.2 vs. IMRT**								
Bhangoo et al. 31 (2020)	10	4.7	32	12.3	5.4	32	5.5%	-2.30 [-4.78, 0.18]
Cui et al. 52 (2022)	8.7	4.1	30	24.2	8.4	30	5.0%	-15.50 [-18.84, -12.16]
Hirano et al. 16 (2018)	11.7	5.2	27	17.8	5.2	27	5.3%	-6.10 [-8.87, -3.33]
Lin et al. 28 (2020)	8.7	3.1	46	14.1	7.9	61	5.6%	-5.40 [-7.58, -3.22]
Ling et al. 20 (2014)	15.3	6.5	10	16.2	5.8	10	3.9%	-0.90 [-6.30, 4.50]
Mu et al. 23 (2011)	14.8	3.5	10	20.9	3.1	10	5.3%	-6.10 [-9.00, -3.20]
Møller et al. 15 (2019)	7.5	4.2	26	14.3	7.3	26	5.1%	-6.80 [-10.04, -3.56]
Welsh et al. 21 (2011)	11	6.3	10	14	6.3	10	3.8%	-3.00 [-8.52, 2.52]
Xi et al. 35 (2017)	11.3	6.1	132	18.4	7.7	211	5.9%	-7.10 [-8.57, -5.63]
Zhang et al. 11 (2021)	21.4	12.8	25	27.4	16.5	25	2.6%	-6.00 [-14.19, 2.19]
Zhang et al. 22 (2008)	10.8	3.2	15	16.8	4.8	15	5.3%	-6.00 [-8.92, -3.08]
Zhang et al. 51 (2022)	5.7	1.8	11	24.4	5	11	5.1%	-18.70 [-21.84, -15.56]
**Subtotal (95% CI)**			**374**			**468**	**58.5%**	**-7.16 [-9.77, -4.55]**
Heterogeneity: Tau² = 17.68; Chi² = 102.16, df = 11 (P < 0.00001); I² = 89%								
Test for overall effect: Z = 5.38 (P < 0.00001)								
**1.4.3 vs. VMAT**								
Anakotta et al. 12 (2020)	6.5	3.6	19	12.1	7.5	19	4.8%	-5.60 [-9.34, -1.86]
Celik et al. 13 (2020)	5.9	2.6	20	10.5	5.6	20	5.4%	-4.60 [-7.31, -1.89]
Cui et al. 52 (2022)	8.7	4.1	30	17.6	8	30	5.1%	-8.90 [-12.12, -5.68]
Kato et al. 50 (2022)	3.2	1.2	10	8.5	4.2	10	5.4%	-5.30 [-8.01, -2.59]
Liu et al. 14 (2019)	8.5	5.2	19	9.1	5.3	16	4.9%	-0.60 [-4.09, 2.89]
Warren et al. 19 (2016)	7.5	4	21	16.3	6.4	21	5.1%	-8.80 [-12.03, -5.57]
Zhang et al. 11 (2021)	21.4	12.8	25	25.2	14.8	25	2.8%	-3.80 [-11.47, 3.87]
**Subtotal (95% CI)**			**144**			**141**	**33.5%**	**-5.55 [-7.71, -3.38]**
Heterogeneity: Tau² = 5.15; Chi² = 16.47, df = 6 (P = 0.01); I² = 64%								
Test for overall effect: Z = 5.02 (P < 0.00001)								
**Total (95% CI)**			**555**			**646**	**100.0%**	**-6.52 [-8.24, -4.79]**
Heterogeneity: Tau² = 12.49; Chi² = 123.36, df = 20 (P < 0.00001); I² = 84%								
Test for overall effect: Z = 7.40 (P < 0.00001)								
Test for subgroup differences: Chi² = 1.07, df = 2 (P = 0.58), I² = 0%								

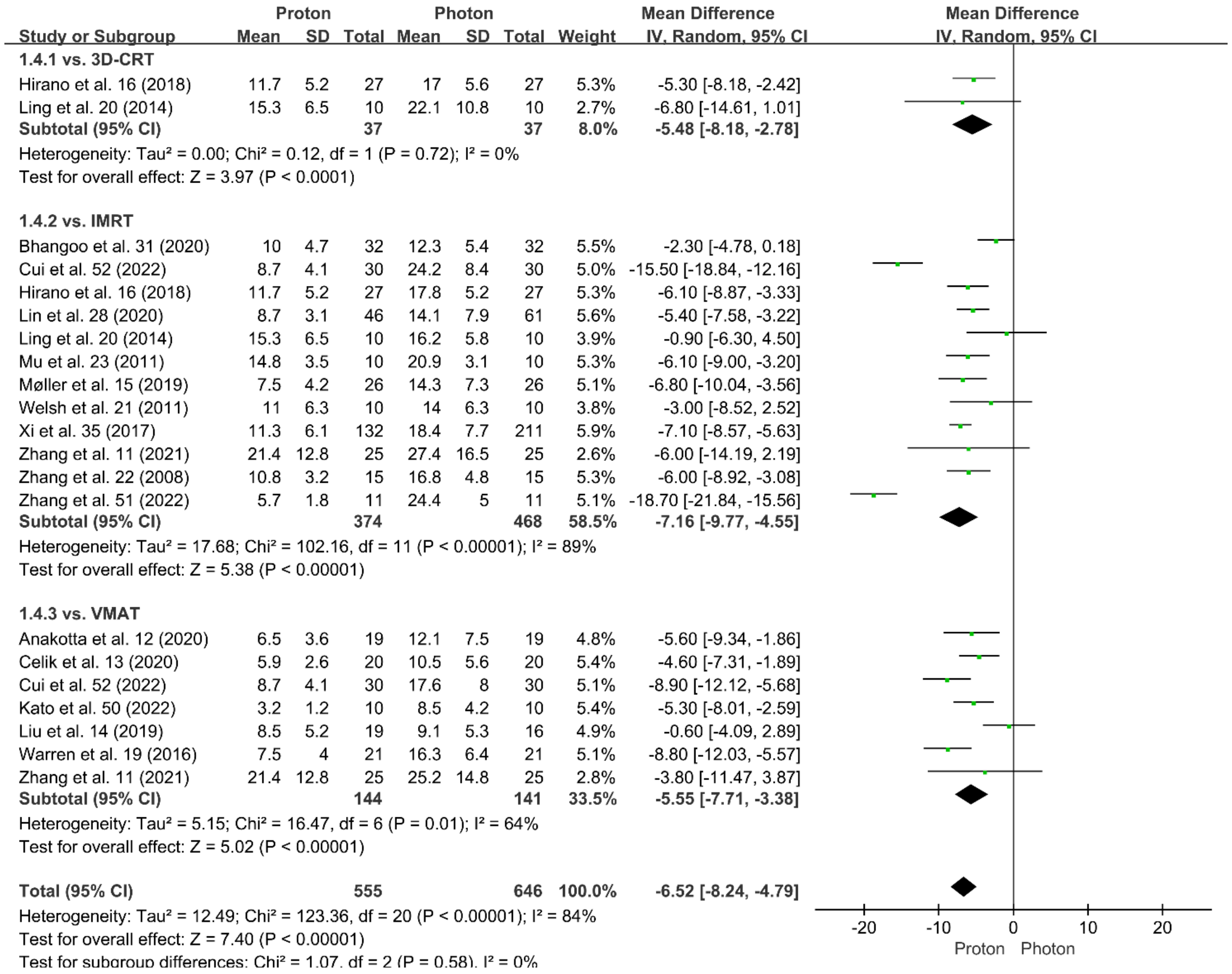

e) Lung-V_{30}

Study or Subgroup	Proton Mean	Proton SD	Proton Total	Photon Mean	Photon SD	Photon Total	Weight	Mean Difference IV, Random, 95% CI
**1.5.1 vs. 3D-CRT**								
Ling et al. 20 (2014)	6.1	2.9	10	9.8	5.1	10	16.9%	-3.70 [-7.34, -0.06]
**Subtotal (95% CI)**			**10**			**10**	**16.9%**	**-3.70 [-7.34, -0.06]**
Heterogeneity: Not applicable								
Test for overall effect: Z = 1.99 (P = 0.05)								
**1.5.2 vs. IMRT**								
Ling et al. 20 (2014)	6.1	2.9	10	6.6	3.2	10	19.4%	-0.50 [-3.18, 2.18]
Zhang et al. 22 (2008)	6.7	2.1	15	7.1	2.2	15	22.0%	-0.40 [-1.94, 1.14]
Zhang et al. 51 (2022)	2.6	1.2	11	12.6	4.6	11	19.0%	-10.00 [-12.81, -7.19]
**Subtotal (95% CI)**			**36**			**36**	**60.4%**	**-3.56 [-9.24, 2.12]**
Heterogeneity: Tau² = 23.70; Chi² = 36.42, df = 2 (P < 0.00001); I² = 95%								
Test for overall effect: Z = 1.23 (P = 0.22)								
**1.5.3 vs. VMAT**								
Kato et al. 50 (2022)	1.9	0.8	10	3.4	1.6	10	22.7%	-1.50 [-2.61, -0.39]
**Subtotal (95% CI)**			**10**			**10**	**22.7%**	**-1.50 [-2.61, -0.39]**
Heterogeneity: Not applicable								
Test for overall effect: Z = 2.65 (P = 0.008)								
**Total (95% CI)**			**56**			**56**	**100.0%**	**-3.05 [-5.86, -0.25]**
Heterogeneity: Tau² = 8.71; Chi² = 38.07, df = 4 (P < 0.00001); I² = 89%								
Test for overall effect: Z = 2.13 (P = 0.03)								
Test for subgroup differences: Chi² = 1.69, df = 2 (P = 0.43), I² = 0%								

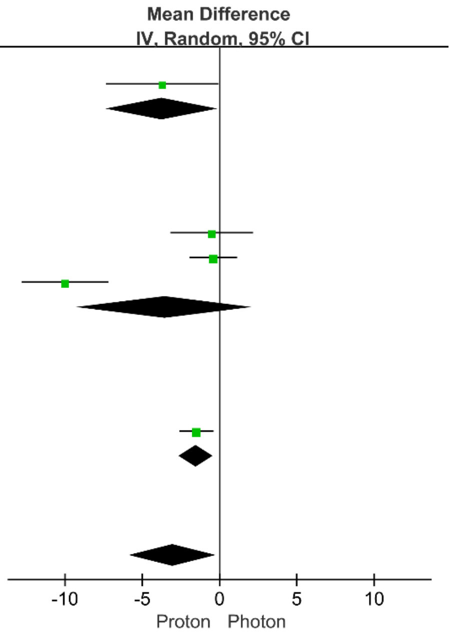

© 2023 Zhou P et al. *JAMA Network Open.*

f) Lung-V_{40}

Study or Subgroup	Proton Mean	Proton SD	Proton Total	Photon Mean	Photon SD	Photon Total	Weight	Mean Difference IV, Fixed, 95% CI
**1.6.1 vs. 3D-CRT**								
Ling et al. 20 (2014)	4.3	2.1	10	4.7	2.9	10	7.4%	-0.40 [-2.62, 1.82]
**Subtotal (95% CI)**			**10**			**10**	**7.4%**	**-0.40 [-2.62, 1.82]**
Heterogeneity: Not applicable								
Test for overall effect: Z = 0.35 (P = 0.72)								
**1.6.2 vs. IMRT**								
Ling et al. 20 (2014)	4.3	2.1	10	3.5	2	10	11.3%	0.80 [-1.00, 2.60]
**Subtotal (95% CI)**			**10**			**10**	**11.3%**	**0.80 [-1.00, 2.60]**
Heterogeneity: Not applicable								
Test for overall effect: Z = 0.87 (P = 0.38)								
**1.6.3 vs. VMAT**								
Kato et al. 50 (2022)	1.1	0.6	10	1.5	0.9	10	81.3%	-0.40 [-1.07, 0.27]
**Subtotal (95% CI)**			**10**			**10**	**81.3%**	**-0.40 [-1.07, 0.27]**
Heterogeneity: Not applicable								
Test for overall effect: Z = 1.17 (P = 0.24)								
**Total (95% CI)**			**30**			**30**	**100.0%**	**-0.26 [-0.87, 0.34]**
Heterogeneity: Chi² = 1.52, df = 2 (P = 0.47); I² = 0%								
Test for overall effect: Z = 0.86 (P = 0.39)								
Test for subgroup differences: Chi² = 1.52, df = 2 (P = 0.47), I² = 0%								

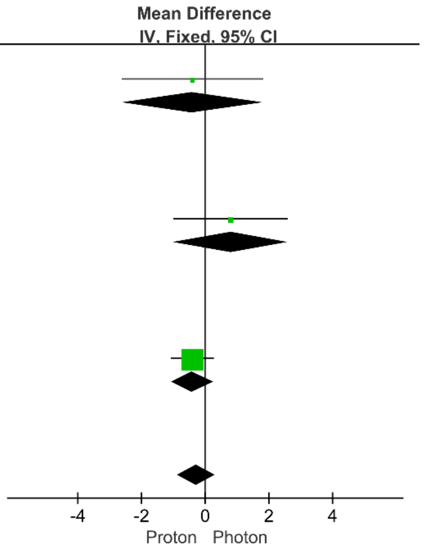

g) Heart-MLD

Study or Subgroup	Proton Mean	Proton SD	Proton Total	Photon Mean	Photon SD	Photon Total	Weight	Mean Difference IV, Random, 95% CI
**2.1.1 vs. 3D-CRT**								
Hirano et al. 16 (2018)	17.6	9.7	27	31	11.5	27	3.3%	-13.40 [-19.07, -7.73]
Lin et al. 37 (2017)	13.2	5.2	111	28.4	7.4	214	5.4%	-15.20 [-16.59, -13.81]
Ling et al. 20 (2014)	12.6	3.9	10	27.5	5.2	10	4.1%	-14.90 [-18.93, -10.87]
**Subtotal (95% CI)**			**148**			**251**	**12.8%**	**-15.08 [-16.36, -13.80]**
Heterogeneity: Tau² = 0.00; Chi² = 0.37, df = 2 (P = 0.83); I² = 0%								
Test for overall effect: Z = 23.15 (P < 0.00001)								
**2.1.2 vs. IMRT**								
Bhangoo et al. 31 (2020)	7.7	4	32	19.5	6.1	32	4.9%	-11.80 [-14.33, -9.27]
Cui et al. 52 (2022)	10.4	4.9	30	21.9	9.6	30	4.2%	-11.50 [-15.36, -7.64]
Hirano et al. 16 (2018)	17.6	9.7	27	9.5	2.2	27	4.3%	8.10 [4.35, 11.85]
Lin et al. 28 (2020)	11.1	4.8	46	19.8	5.4	61	5.2%	-8.70 [-10.64, -6.76]
Lin et al. 37 (2017)	13.2	5.2	111	22.4	5.2	255	5.4%	-9.20 [-10.36, -8.04]
Ling et al. 20 (2014)	12.6	3.9	10	28.5	5.5	10	4.0%	-15.90 [-20.08, -11.72]
Møller et al. 15 (2019)	8.4	3.9	26	14.8	5.8	26	4.8%	-6.40 [-9.09, -3.71]
Shiraishi et al. 17 2017	13.3	5.1	250	23.7	6.9	477	5.5%	-10.40 [-11.28, -9.52]
Wang et al. 29 (2020)	12.5	5	159	21.6	7	320	5.5%	-9.10 [-10.19, -8.01]
Welsh et al. 21 (2011)	11.9	3.1	10	21.2	5	10	4.3%	-9.30 [-12.95, -5.65]
Xi et al. 35 (2017)	11.6	6.3	132	19.9	8.3	211	5.3%	-8.30 [-9.85, -6.75]
Zhang et al. 11 (2021)	16.4	8.3	25	20.3	9.3	25	3.7%	-3.90 [-8.79, 0.99]
Zhang et al. 51 (2022)	3.7	2.5	11	22.1	9.1	11	3.3%	-18.40 [-23.98, -12.82]
**Subtotal (95% CI)**			**869**			**1495**	**60.4%**	**-8.74 [-10.53, -6.94]**
Heterogeneity: Tau² = 8.58; Chi² = 124.67, df = 12 (P < 0.00001); I² = 90%								
Test for overall effect: Z = 9.54 (P < 0.00001)								
**2.1.3 vs. VMAT**								
Anakotta et al. 12 (2020)	8.6	3	19	20.3	4.1	19	5.0%	-11.70 [-13.98, -9.42]
Celik et al. 13 (2020)	4	1.4	20	9.9	1.9	20	5.5%	-5.90 [-6.93, -4.87]
Cui et al. 52 (2022)	10.4	4.9	30	24.9	14.3	30	3.4%	-14.50 [-19.91, -9.09]
Liu et al. 14 (2019)	8.2	4.1	19	20.8	7.6	16	4.0%	-12.60 [-16.76, -8.44]
Warren et al. 19 (2016)	13	3.8	21	21.5	4.1	21	5.0%	-8.50 [-10.89, -6.11]
Zhang et al. 11 (2021)	16.4	8.3	25	17.3	7.8	25	3.9%	-0.90 [-5.36, 3.56]
**Subtotal (95% CI)**			**134**			**131**	**26.8%**	**-8.86 [-12.02, -5.69]**
Heterogeneity: Tau² = 12.64; Chi² = 43.20, df = 5 (P < 0.00001); I² = 88%								
Test for overall effect: Z = 5.48 (P < 0.00001)								
**Total (95% CI)**			**1151**			**1877**	**100.0%**	**-9.52 [-11.11, -7.94]**
Heterogeneity: Tau² = 11.69; Chi² = 269.34, df = 21 (P < 0.00001); I² = 92%								
Test for overall effect: Z = 11.76 (P < 0.00001)								
Test for subgroup differences: Chi² = 37.67, df = 2 (P < 0.00001), I² = 94.7%								

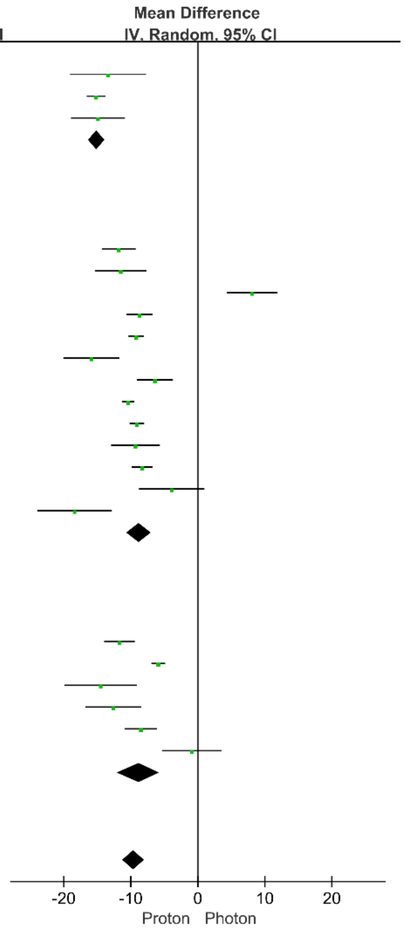

h) Heart-V_5

Study or Subgroup	Proton Mean	Proton SD	Proton Total	Photon Mean	Photon SD	Photon Total	Weight	Mean Difference IV, Random, 95% CI
**2.2.1 vs. IMRT**								
Cui et al. 52 (2022)	46.2	23.5	30	75.7	32.1	30	11.5%	-29.50 [-43.74, -15.26]
Shiraishi et al. 17 2017	41	14.8	250	90.6	13.5	477	29.2%	-49.60 [-51.80, -47.40]
Wang et al. 29 (2020)	41.7	18.7	159	87.1	20.2	320	27.4%	-45.40 [-49.05, -41.75]
**Subtotal (95% CI)**			**439**			**827**	**68.1%**	**-45.29 [-51.22, -39.37]**
Heterogeneity: Tau² = 18.65; Chi² = 10.44, df = 2 (P = 0.005); I² = 81%								
Test for overall effect: Z = 14.98 (P < 0.00001)								
**2.2.2 vs. VMAT**								
Anakotta et al. 12 (2020)	34.4	11.2	19	90.7	12.5	19	20.8%	-56.30 [-63.85, -48.75]
Cui et al. 52 (2022)	46.2	23.5	30	73.5	33.6	30	11.1%	-27.30 [-41.97, -12.63]
**Subtotal (95% CI)**			**49**			**49**	**31.9%**	**-42.51 [-70.90, -14.13]**
Heterogeneity: Tau² = 385.07; Chi² = 11.87, df = 1 (P = 0.0006); I² = 92%								
Test for overall effect: Z = 2.94 (P = 0.003)								
**Total (95% CI)**			**488**			**876**	**100.0%**	**-45.06 [-51.18, -38.94]**
Heterogeneity: Tau² = 32.13; Chi² = 22.65, df = 4 (P = 0.0001); I² = 82%								
Test for overall effect: Z = 14.42 (P < 0.00001)								
Test for subgroup differences: Chi² = 0.04, df = 1 (P = 0.85), I² = 0%								

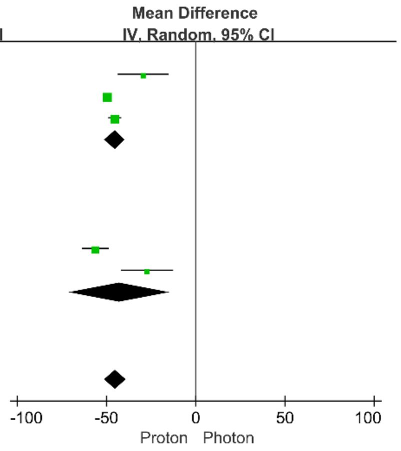

i) Heart-V_{10}

Study or Subgroup	Proton Mean	Proton SD	Proton Total	Photon Mean	Photon SD	Photon Total	Weight	Mean Difference IV, Random, 95% CI
**2.3.1 vs. IMRT**								
Cui et al. 52 (2022)	36.3	18.9	30	67.2	30.8	30	18.9%	-30.90 [-43.83, -17.97]
Shiraishi et al. 17 2017	35.9	14.3	250	78.3	17.5	477	36.7%	-42.40 [-44.77, -40.03]
Welsh et al. 21 (2011)	29	6.3	10	79	12.6	10	26.0%	-50.00 [-58.73, -41.27]
**Subtotal (95% CI)**			**290**			**517**	**81.5%**	**-42.36 [-49.92, -34.79]**
Heterogeneity: Tau² = 28.99; Chi² = 5.93, df = 2 (P = 0.05); I² = 66%								
Test for overall effect: Z = 10.97 (P < 0.00001)								
**2.3.2 vs. AMAT**								
Cui et al. 52 (2022)	36.3	18.9	30	64.2	31.7	30	18.5%	-27.90 [-41.11, -14.69]
**Subtotal (95% CI)**			**30**			**30**	**18.5%**	**-27.90 [-41.11, -14.69]**
Heterogeneity: Not applicable								
Test for overall effect: Z = 4.14 (P < 0.0001)								
**Total (95% CI)**			**320**			**547**	**100.0%**	**-39.52 [-47.45, -31.60]**
Heterogeneity: Tau² = 43.12; Chi² = 10.53, df = 3 (P = 0.01); I² = 72%								
Test for overall effect: Z = 9.77 (P < 0.00001)								
Test for subgroup differences: Chi² = 3.47, df = 1 (P = 0.06), I² = 71.1%								

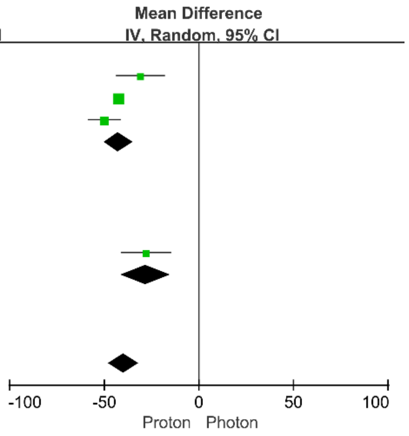

© 2023 Zhou P et al. *JAMA Network Open.*

j) Heart-V_{20}

Study or Subgroup	Proton Mean	Proton SD	Proton Total	Photon Mean	Photon SD	Photon Total	Weight	Mean Difference IV, Random, 95% CI
**2.4.1 vs. 3D-CRT**								
Hirano et al. 16 (2018)	38.5	23.9	27	64.1	23	27	9.9%	-25.60 [-38.11, -13.09]
**Subtotal (95% CI)**			**27**			**27**	**9.9%**	**-25.60 [-38.11, -13.09]**
Heterogeneity: Not applicable								
Test for overall effect: Z = 4.01 (P < 0.0001)								
**2.4.2 vs. IMRT**								
Cui et al. 52 (2022)	16.8	8.7	30	49.7	25.3	30	14.5%	-32.90 [-42.47, -23.33]
Hirano et al. 16 (2018)	38.5	23.9	27	69.3	27	27	8.7%	-30.80 [-44.40, -17.20]
Shiraishi et al. 17 2017	27.8	11.6	250	50.8	20.7	477	38.1%	-23.00 [-25.35, -20.65]
Welsh et al. 21 (2011)	22	6.3	10	44	12.6	10	16.3%	-22.00 [-30.73, -13.27]
**Subtotal (95% CI)**			**317**			**544**	**77.5%**	**-25.36 [-30.17, -20.55]**
Heterogeneity: Tau² = 10.17; Chi² = 5.08, df = 3 (P = 0.17); I² = 41%								
Test for overall effect: Z = 10.33 (P < 0.00001)								
**2.4.3 vs. VMAT**								
Cui et al. 52 (2022)	16.8	8.7	30	50.9	28.3	30	12.6%	-34.10 [-44.69, -23.51]
**Subtotal (95% CI)**			**30**			**30**	**12.6%**	**-34.10 [-44.69, -23.51]**
Heterogeneity: Not applicable								
Test for overall effect: Z = 6.31 (P < 0.00001)								
**Total (95% CI)**			**374**			**601**	**100.0%**	**-26.60 [-31.09, -22.12]**
Heterogeneity: Tau² = 12.31; Chi² = 8.72, df = 5 (P = 0.12); I² = 43%								
Test for overall effect: Z = 11.62 (P < 0.00001)								
Test for subgroup differences: Chi² = 2.20, df = 2 (P = 0.33), I² = 9.2%								

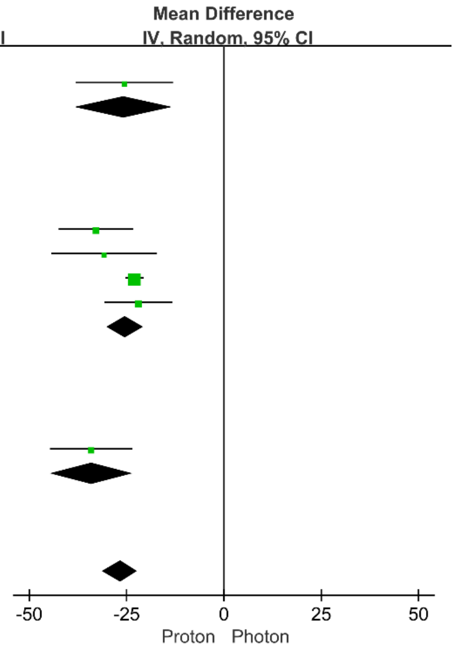

k) Heart-V_{30}

Study or Subgroup	Proton Mean	Proton SD	Proton Total	Photon Mean	Photon SD	Photon Total	Weight	Mean Difference IV, Random, 95% CI
**2.5.1 vs. 3D-CRT**								
Hirano et al. 16 (2018)	22	14.5	27	56.2	21.5	27	5.8%	-34.20 [-43.98, -24.42]
Ling et al. 20 (2014)	20.9	7.1	10	32.7	9.4	10	6.8%	-11.80 [-19.10, -4.50]
**Subtotal (95% CI)**			**37**			**37**	**12.6%**	**-22.75 [-44.70, -0.81]**
Heterogeneity: Tau² = 231.49; Chi² = 12.94, df = 1 (P = 0.0003); I² = 92%								
Test for overall effect: Z = 2.03 (P = 0.04)								
**2.5.2 vs. IMRT**								
Bhangoo et al. 31 (2020)	13	5.4	32	21	12.4	32	7.9%	-8.00 [-12.69, -3.31]
Cui et al. 52 (2022)	10	5.6	30	30.8	16.7	30	7.3%	-20.80 [-27.10, -14.50]
Hirano et al. 16 (2018)	22	14.5	27	50.7	23.4	27	5.5%	-28.70 [-39.08, -18.32]
Ling et al. 20 (2014)	20.9	7.1	10	42.3	15.3	10	5.5%	-21.40 [-31.85, -10.95]
Shiraishi et al. 17 2017	21.5	8.5	250	32.3	17.9	477	8.7%	-10.80 [-12.72, -8.88]
Wang et al. 29 (2020)	19.3	8.7	159	27.5	16.7	320	8.7%	-8.20 [-10.48, -5.92]
Welsh et al. 21 (2011)	17	6.3	10	25	9.5	10	6.9%	-8.00 [-15.07, -0.93]
Xi et al. 35 (2017)	18.9	11.6	132	24.4	14.7	211	8.5%	-5.50 [-8.30, -2.70]
Zhang et al. 51 (2022)	5	3.8	11	26.8	11.8	11	6.8%	-21.80 [-29.13, -14.47]
**Subtotal (95% CI)**			**661**			**1128**	**65.8%**	**-13.07 [-16.67, -9.48]**
Heterogeneity: Tau² = 21.41; Chi² = 52.24, df = 8 (P < 0.00001); I² = 85%								
Test for overall effect: Z = 7.13 (P < 0.00001)								
**2.5.3 vs. VMAT**								
Celik et al. 13 (2020)	4.9	2.3	20	5.9	2.9	20	8.8%	-1.00 [-2.62, 0.62]
Cui et al. 52 (2022)	10	5.6	30	38.3	28.8	30	5.5%	-28.30 [-38.80, -17.80]
Liu et al. 14 (2019)	12.3	7.1	19	18.2	10.2	16	7.4%	-5.90 [-11.83, 0.03]
**Subtotal (95% CI)**			**69**			**66**	**21.6%**	**-10.51 [-22.16, 1.14]**
Heterogeneity: Tau² = 94.45; Chi² = 27.22, df = 2 (P < 0.00001); I² = 93%								
Test for overall effect: Z = 1.77 (P = 0.08)								
**Total (95% CI)**			**767**			**1231**	**100.0%**	**-13.89 [-17.84, -9.95]**
Heterogeneity: Tau² = 45.41; Chi² = 175.07, df = 13 (P < 0.00001); I² = 93%								
Test for overall effect: Z = 6.91 (P < 0.00001)								
Test for subgroup differences: Chi² = 0.93, df = 2 (P = 0.63), I² = 0%								

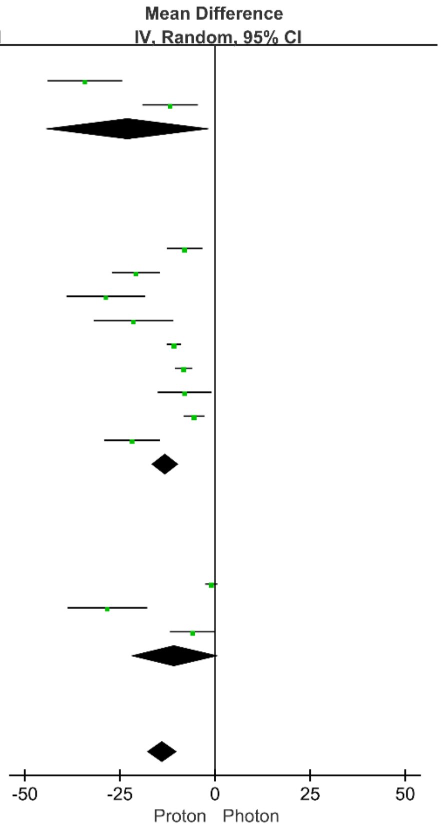

l) Heart-V_{40}

Study or Subgroup	Proton Mean	Proton SD	Proton Total	Photon Mean	Photon SD	Photon Total	Weight	Mean Difference IV, Random, 95% CI
**2.6.1 vs. 3D-CRT**								
Hirano et al. 16 (2018)	16.3	11.4	27	48.2	21	27	5.6%	-31.90 [-40.91, -22.89]
Ling et al. 20 (2014)	16.2	6.4	10	25.8	8.8	10	6.9%	-9.60 [-16.34, -2.86]
**Subtotal (95% CI)**			**37**			**37**	**12.5%**	**-20.54 [-42.39, 1.31]**
Heterogeneity: Tau² = 232.15; Chi² = 15.08, df = 1 (P = 0.0001); I² = 93%								
Test for overall effect: Z = 1.84 (P = 0.07)								
**2.6.2 vs. IMRT**								
Cui et al. 52 (2022)	6.9	4.2	30	16.8	9.6	30	8.8%	-9.90 [-13.65, -6.15]
Hirano et al. 16 (2018)	16.3	11.4	27	26.6	16.9	27	6.4%	-10.30 [-17.99, -2.61]
Ling et al. 20 (2014)	16.2	6.4	10	25.5	11	10	6.2%	-9.30 [-17.19, -1.41]
Møller et al. 15 (2019)	8.7	5	26	8.9	5.3	26	9.3%	-0.20 [-3.00, 2.60]
Shiraishi et al. 17 2017	16.1	7	250	19.6	11.8	477	9.8%	-3.50 [-4.87, -2.13]
Welsh et al. 21 (2011)	13	3.2	10	15	6.3	10	8.4%	-2.00 [-6.38, 2.38]
Xi et al. 35 (2017)	14	9.5	132	14.3	9.6	211	9.6%	-0.30 [-2.37, 1.77]
Zhang et al. 22 (2008)	30.3	10.9	15	35.9	11.1	15	6.2%	-5.60 [-13.47, 2.27]
Zhang et al. 51 (2022)	3.4	2.8	11	15.4	7.2	11	8.3%	-12.00 [-16.57, -7.43]
**Subtotal (95% CI)**			**511**			**817**	**73.0%**	**-5.17 [-7.92, -2.43]**
Heterogeneity: Tau² = 12.17; Chi² = 44.72, df = 8 (P < 0.00001); I² = 82%								
Test for overall effect: Z = 3.69 (P = 0.0002)								
**2.6.3 vs. VMAT**								
Cui et al. 52 (2022)	6.9	4.2	30	28.2	25.6	30	5.4%	-21.30 [-30.58, -12.02]
Liu et al. 14 (2019)	8.9	4.3	19	6.9	5.3	16	9.1%	2.00 [-1.24, 5.24]
**Subtotal (95% CI)**			**49**			**46**	**14.5%**	**-9.23 [-32.05, 13.59]**
Heterogeneity: Tau² = 258.86; Chi² = 21.58, df = 1 (P < 0.00001); I² = 95%								
Test for overall effect: Z = 0.79 (P = 0.43)								
**Total (95% CI)**			**597**			**900**	**100.0%**	**-7.44 [-10.66, -4.22]**
Heterogeneity: Tau² = 27.04; Chi² = 111.28, df = 12 (P < 0.00001); I² = 89%								
Test for overall effect: Z = 4.53 (P < 0.00001)								
Test for subgroup differences: Chi² = 1.98, df = 2 (P = 0.37), I² = 0%								

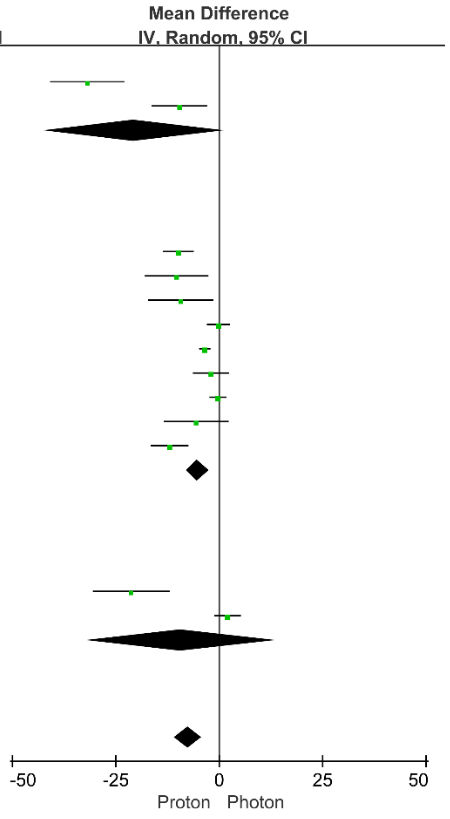

© 2023 Zhou P et al. *JAMA Network Open.*

m) Heart-V_{50}

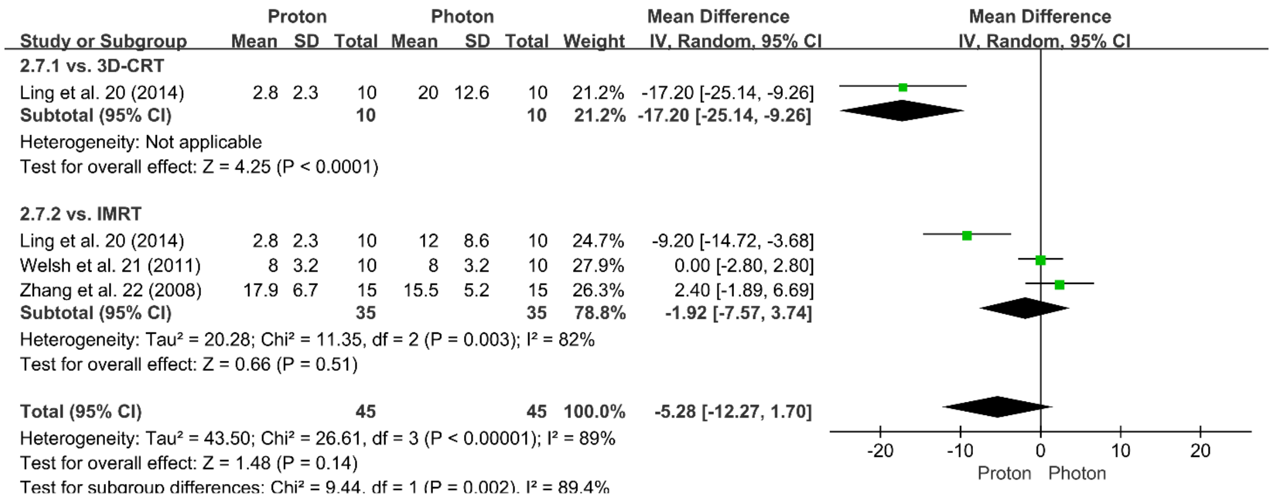

n) Left ventricle (LV)-D_{mean}

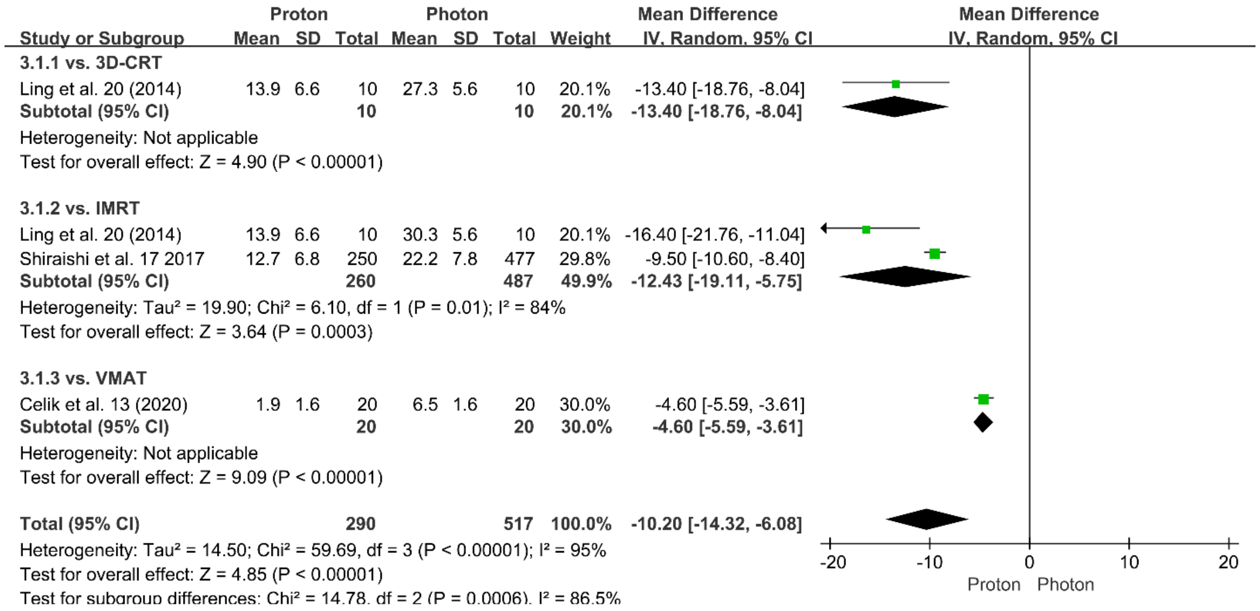

o) Left anterior descending artery (LAD)-D_{mean}

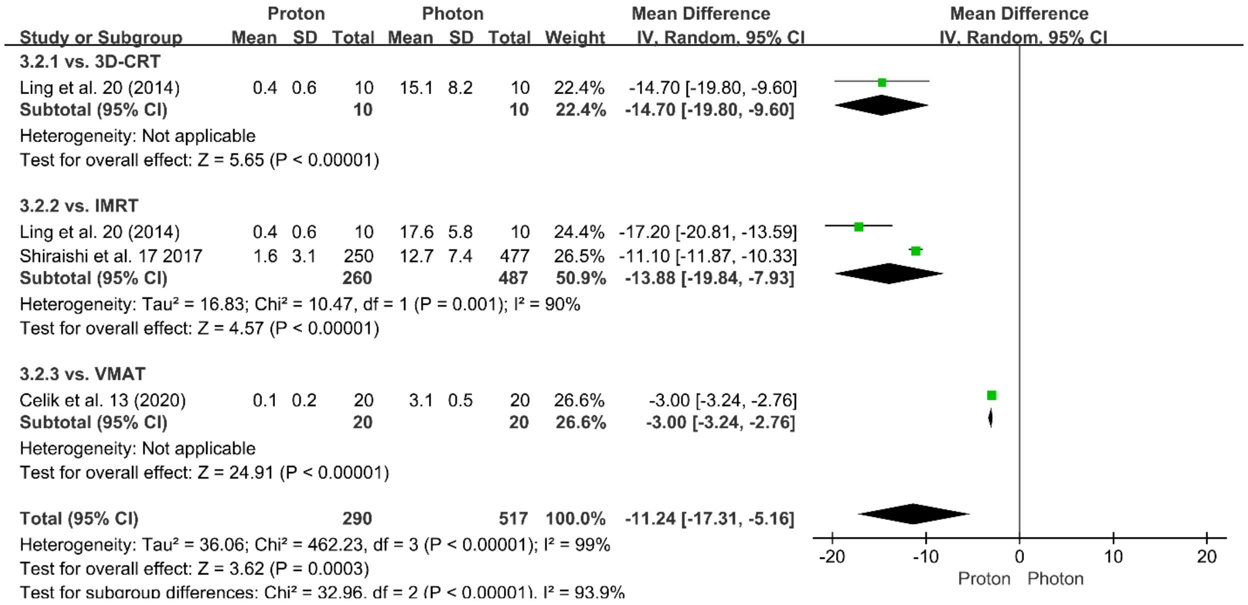

p) BM-D_{mean}

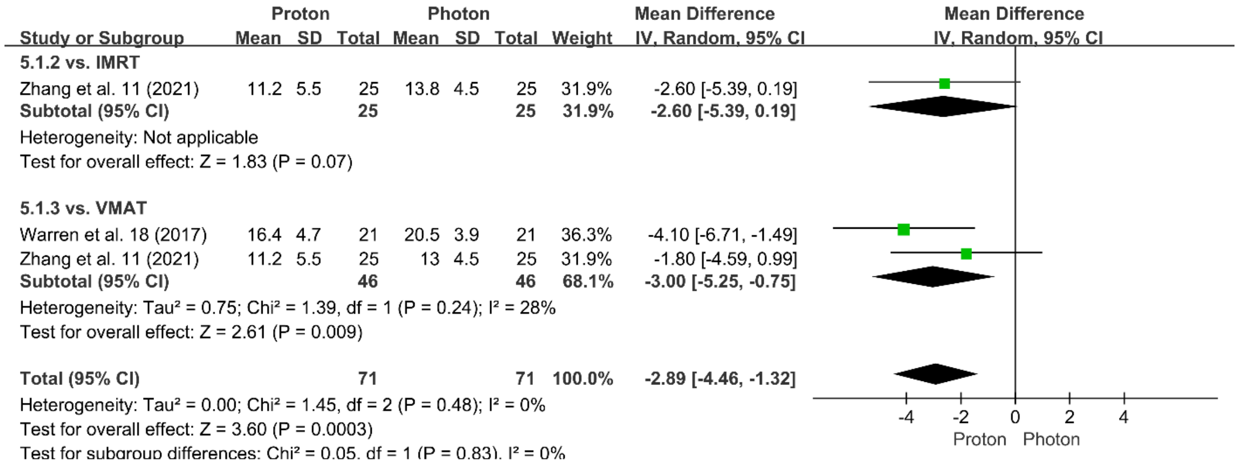

q) BM-V_{10}

© 2023 Zhou P et al. *JAMA Network Open.*

Study or Subgroup	Proton Mean	Proton SD	Proton Total	Photon Mean	Photon SD	Photon Total	Weight	Mean Difference IV, Random, 95% CI
**5.2.1 vs. 3D-CRT**								
Sumiya et al. 27 (2021)	49	10.4	54	57.6	12.6	15	29.7%	-8.60 [-15.55, -1.65]
**Subtotal (95% CI)**			**54**			**15**	**29.7%**	**-8.60 [-15.55, -1.65]**
Heterogeneity: Not applicable								
Test for overall effect: Z = 2.42 (P = 0.02)								
**5.2.2 vs. IMRT**								
Zhang et al. 11 (2021)	31.1	17.1	25	40.8	17.5	25	15.6%	-9.70 [-19.29, -0.11]
**Subtotal (95% CI)**			**25**			**25**	**15.6%**	**-9.70 [-19.29, -0.11]**
Heterogeneity: Not applicable								
Test for overall effect: Z = 1.98 (P = 0.05)								
**5.2.3 vs. VMAT**								
Warren et al. 18 (2017)	43	10.2	21	55.7	9.9	21	38.8%	-12.70 [-18.78, -6.62]
Zhang et al. 11 (2021)	31.1	17.1	25	38.7	17.2	25	15.9%	-7.60 [-17.11, 1.91]
**Subtotal (95% CI)**			**46**			**46**	**54.7%**	**-11.22 [-16.34, -6.10]**
Heterogeneity: Tau² = 0.00; Chi² = 0.78, df = 1 (P = 0.38); I² = 0%								
Test for overall effect: Z = 4.29 (P < 0.0001)								
**Total (95% CI)**			**125**			**86**	**100.0%**	**-10.21 [-13.99, -6.42]**
Heterogeneity: Tau² = 0.00; Chi² = 1.15, df = 3 (P = 0.76); I² = 0%								
Test for overall effect: Z = 5.28 (P < 0.00001)								
Test for subgroup differences: Chi² = 0.37, df = 2 (P = 0.83), I² = 0%								

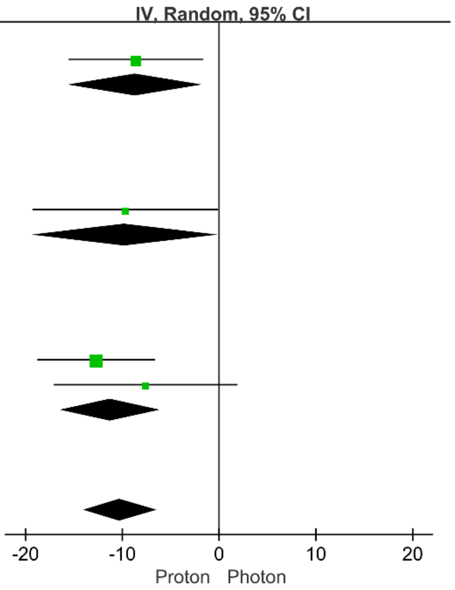

r) BM-V_{20}

Study or Subgroup	Proton Mean	Proton SD	Proton Total	Photon Mean	Photon SD	Photon Total	Weight	Mean Difference IV, Random, 95% CI
**5.3.1 vs. 3D-CRT**								
Sumiya et al. 27 (2021)	34.3	8.7	54	50.8	12.7	15	24.4%	-16.50 [-23.33, -9.67]
**Subtotal (95% CI)**			**54**			**15**	**24.4%**	**-16.50 [-23.33, -9.67]**
Heterogeneity: Not applicable								
Test for overall effect: Z = 4.73 (P < 0.00001)								
**5.3.2 vs. IMRT**								
Zhang et al. 11 (2021)	22.8	12.3	25	28.3	11.5	25	24.9%	-5.50 [-12.10, 1.10]
**Subtotal (95% CI)**			**25**			**25**	**24.9%**	**-5.50 [-12.10, 1.10]**
Heterogeneity: Not applicable								
Test for overall effect: Z = 1.63 (P = 0.10)								
**5.3.3 vs. VMAT**								
Warren et al. 18 (2017)	34.7	10.8	21	49.1	8.6	21	26.6%	-14.40 [-20.30, -8.50]
Zhang et al. 11 (2021)	22.8	12.3	25	27.4	12.8	25	24.1%	-4.60 [-11.56, 2.36]
**Subtotal (95% CI)**			**46**			**46**	**50.7%**	**-9.68 [-19.28, -0.08]**
Heterogeneity: Tau² = 37.18; Chi² = 4.43, df = 1 (P = 0.04); I² = 77%								
Test for overall effect: Z = 1.98 (P = 0.05)								
**Total (95% CI)**			**125**			**86**	**100.0%**	**-10.33 [-16.20, -4.47]**
Heterogeneity: Tau² = 24.61; Chi² = 9.60, df = 3 (P = 0.02); I² = 69%								
Test for overall effect: Z = 3.45 (P = 0.0006)								
Test for subgroup differences: Chi² = 5.19, df = 2 (P = 0.07), I² = 61.5%								

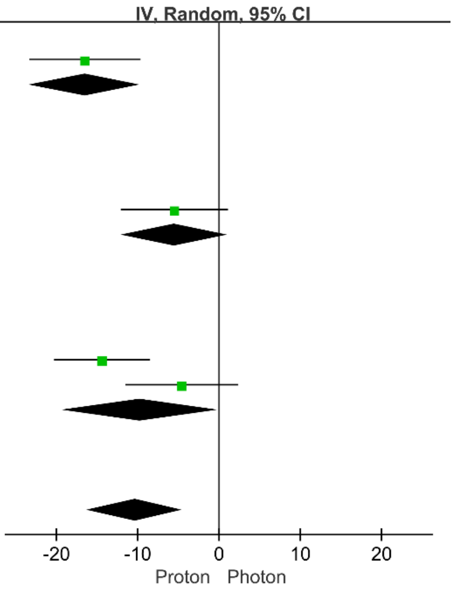

s) SC-D_{max}

Study or Subgroup	Proton Mean	Proton SD	Proton Total	Photon Mean	Photon SD	Photon Total	Weight	Mean Difference IV, Random, 95% CI
**4.1.1 vs. 3D-CRT**								
Hirano et al. 16 (2018)	38.1	5	27	47.3	2.2	27	7.7%	-9.20 [-11.26, -7.14]
Ling et al. 20 (2014)	11.6	10	10	31.2	9.7	10	4.6%	-19.60 [-28.23, -10.97]
**Subtotal (95% CI)**			**37**			**37**	**12.3%**	**-13.52 [-23.56, -3.48]**
Heterogeneity: Tau² = 43.82; Chi² = 5.27, df = 1 (P = 0.02); I² = 81%								
Test for overall effect: Z = 2.64 (P = 0.008)								
**4.1.2 vs. IMRT**								
Cui et al. 52 (2022)	40.2	3	30	45	3.3	30	7.9%	-4.80 [-6.40, -3.20]
Hirano et al. 16 (2018)	38.1	5	27	39.7	2.6	27	7.7%	-1.60 [-3.73, 0.53]
Lin et al. 28 (2020)	38.3	4.5	46	37.1	8	61	7.6%	1.20 [-1.19, 3.59]
Ling et al. 20 (2014)	11.6	10	10	36.9	3.5	10	5.6%	-25.30 [-31.87, -18.73]
Mu et al. 23 (2011)	34.9	5	10	41.4	1	10	7.3%	-6.50 [-9.66, -3.34]
Welsh et al. 21 (2011)	36.8	11.8	10	36.7	9.5	10	4.2%	0.10 [-9.29, 9.49]
Zhang et al. 11 (2021)	31.9	13	25	37.6	6.5	25	6.1%	-5.70 [-11.40, -0.00]
Zhang et al. 22 (2008)	25.4	8.4	15	40.6	1.9	15	6.8%	-15.20 [-19.56, -10.84]
Zhang et al. 51 (2022)	37.6	7.3	11	46.4	3.6	11	6.5%	-8.80 [-13.61, -3.99]
**Subtotal (95% CI)**			**184**			**199**	**59.7%**	**-7.14 [-10.95, -3.34]**
Heterogeneity: Tau² = 28.36; Chi² = 96.46, df = 8 (P < 0.00001); I² = 92%								
Test for overall effect: Z = 3.68 (P = 0.0002)								
**4.1.3 vs. VMAT**								
Anakotta et al. 12 (2020)	30.6	6	19	36.8	5.5	19	7.1%	-6.20 [-9.86, -2.54]
Cui et al. 52 (2022)	40.2	3	30	49.1	6.8	30	7.5%	-8.90 [-11.56, -6.24]
Liu et al. 14 (2019)	38.4	3.6	19	36.1	5.8	16	7.3%	2.30 [-0.97, 5.57]
Zhang et al. 11 (2021)	31.9	13	25	34.2	6.1	25	6.1%	-2.30 [-7.93, 3.33]
**Subtotal (95% CI)**			**93**			**90**	**28.0%**	**-3.86 [-9.39, 1.67]**
Heterogeneity: Tau² = 27.95; Chi² = 28.51, df = 3 (P < 0.00001); I² = 89%								
Test for overall effect: Z = 1.37 (P = 0.17)								
**Total (95% CI)**			**314**			**326**	**100.0%**	**-6.93 [-9.73, -4.13]**
Heterogeneity: Tau² = 25.28; Chi² = 153.11, df = 14 (P < 0.00001); I² = 91%								
Test for overall effect: Z = 4.85 (P < 0.00001)								
Test for subgroup differences: Chi² = 2.83, df = 2 (P = 0.24), I² = 29.4%								

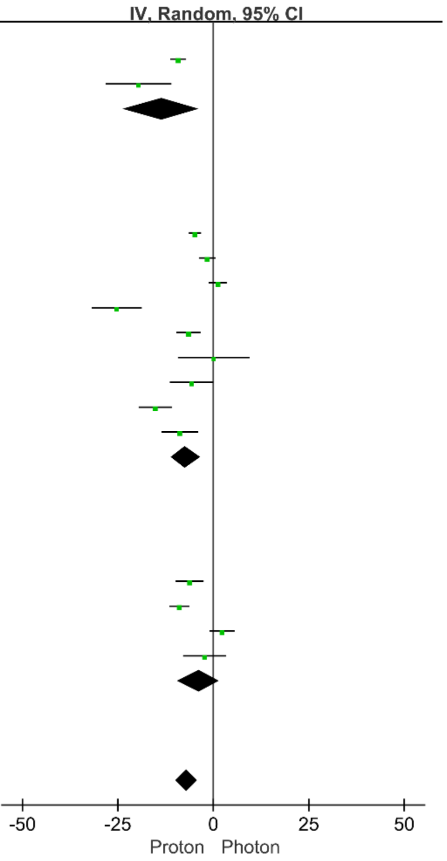

© 2023 Zhou P et al. *JAMA Network Open.*

eFigure 2. Funnel Plot of Dose-Volume Parameters for OARs with Proton vs Photon Therapy (≥10 Articles)

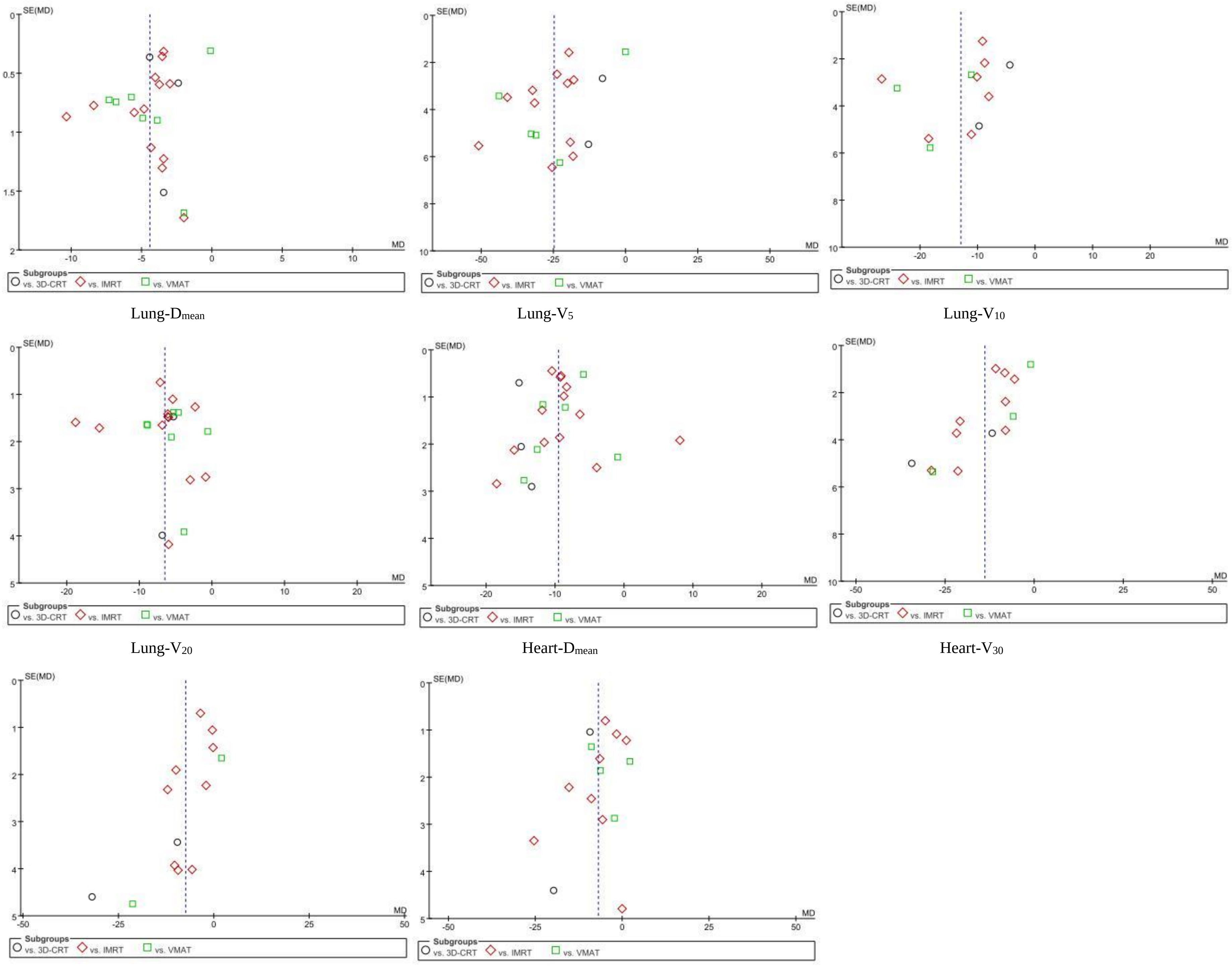

Lung-D_{mean} Lung-V_5 Lung-V_{10}

Lung-V_{20} Heart-D_{mean} Heart-V_{30}

Heart-V_{40} Spinal cord-D_{max}

eFigure 3. Pooled Analysis of pCR (Proton vs Photon Therapy)

Study or Subgroup	Proton therapy Events	Proton therapy Total	Photon therapy Events	Photon therapy Total	Weight	Odds Ratio M-H, Fixed, 95% CI
Bhangoo et al.[31] 2020	5	15	7	18	20.2%	0.79 [0.19, 3.29]
Choi et al.[49] 2022	4	14	5	14	17.0%	0.72 [0.15, 3.54]
DeCesaris et al.[30] 2020	3	18	8	36	21.2%	0.70 [0.16, 3.04]
Lin et al.[28] 2020	14	21	18	30	23.6%	1.33 [0.42, 4.27]
Macomber et al.[34] 2018	3	16	8	39	18.0%	0.89 [0.20, 3.91]
**Total (95% CI)**		**84**		**137**	**100.0%**	**0.90 [0.49, 1.69]**
Total events	29		46			

Heterogeneity: Chi² = 0.66, df = 4 (P = 0.96); I² = 0%
Test for overall effect: Z = 0.32 (P = 0.75)

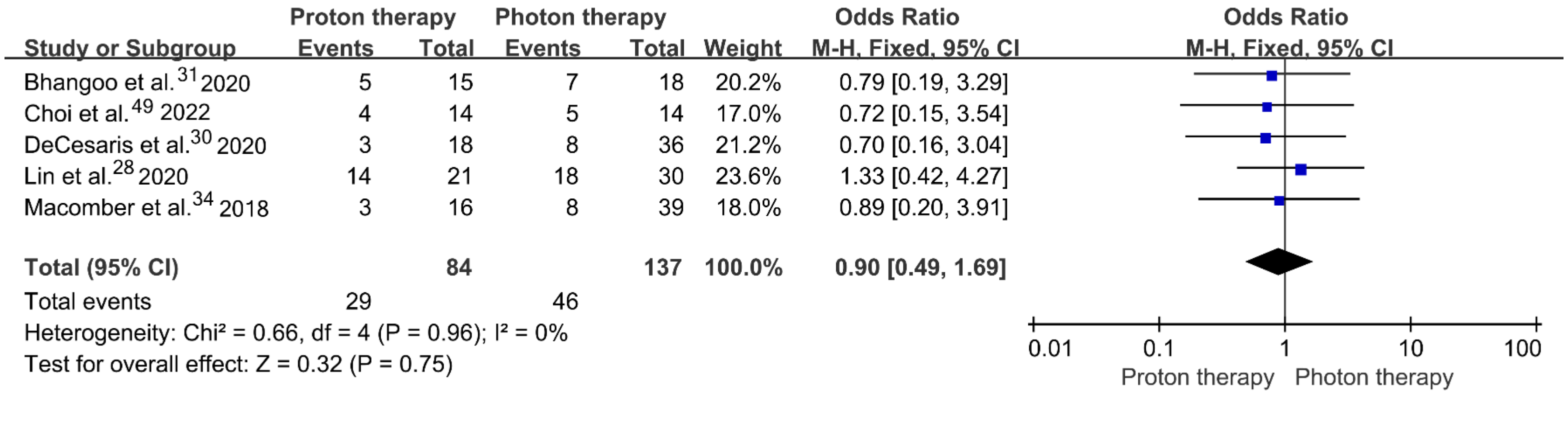

© 2023 Zhou P et al. *JAMA Network Open.*

eFigure 4. Forest Plots of the Incidence of Toxic Effects in the Proton and Photon Therapy Groups (Described as

a) G2+ RE

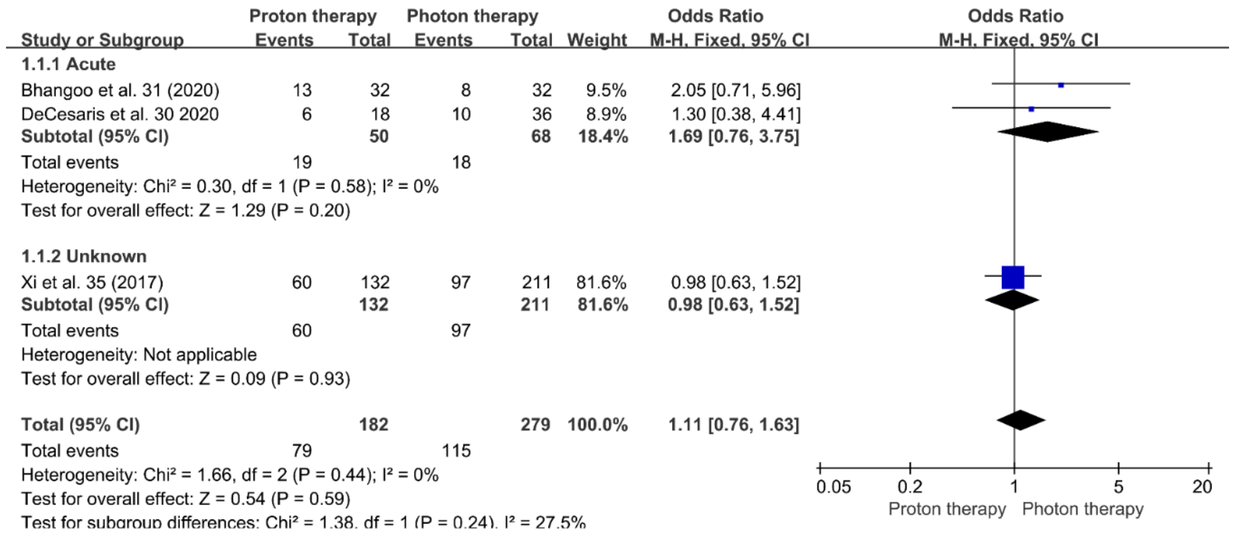

b) G2+ RP

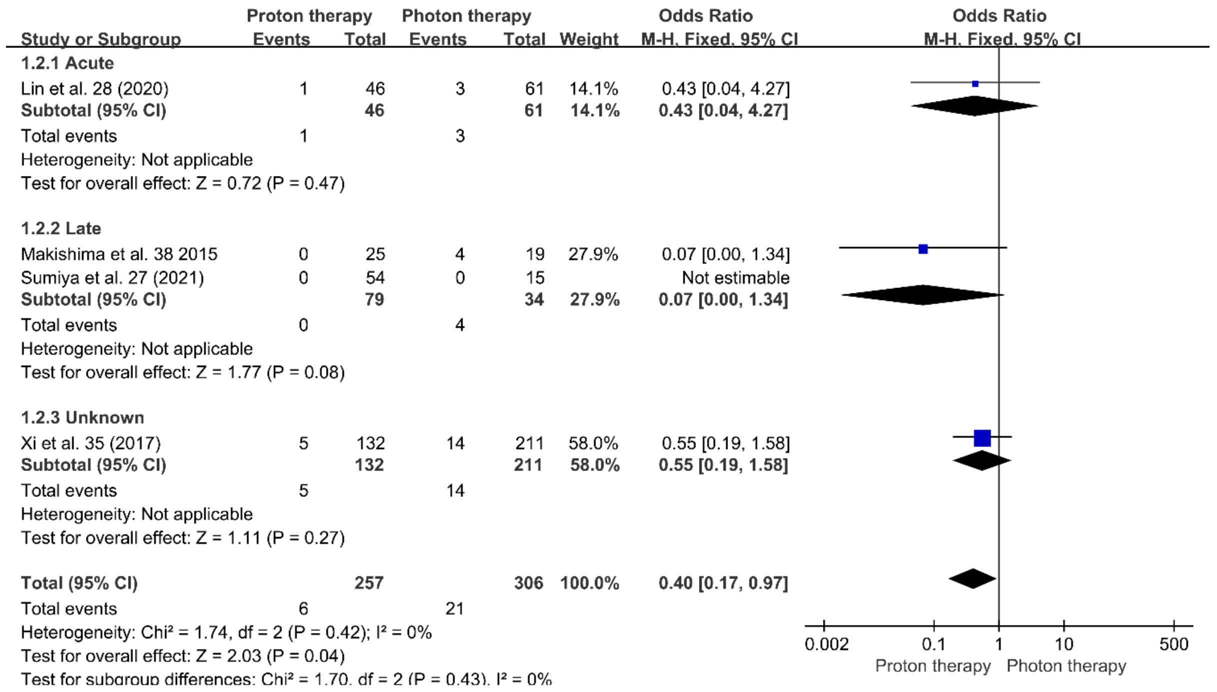

c) G2+ PE

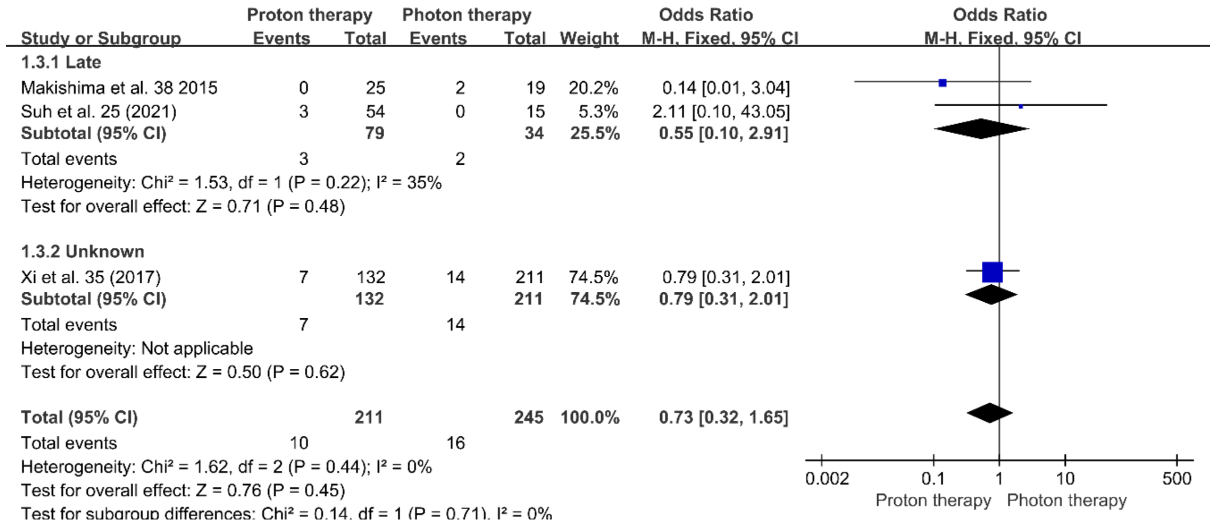

d) G2+ PCE

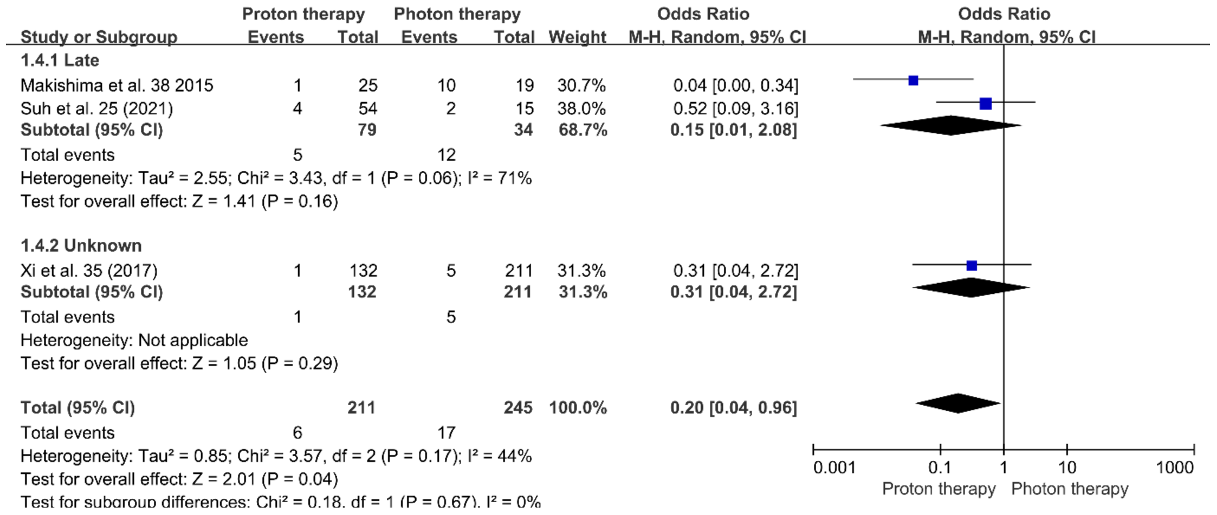

© 2023 Zhou P et al. *JAMA Network Open.*

e) G3+ RE

Study or Subgroup	Proton therapy Events	Proton therapy Total	Photon therapy Events	Photon therapy Total	Weight	Odds Ratio M-H, Fixed, 95% CI
**2.1.1 Acute**						
Bhangoo et al. 31 (2020)	1	32	0	32	1.7%	3.10 [0.12, 78.87]
Lin et al. 28 (2020)	6	46	8	61	21.7%	0.99 [0.32, 3.09]
**Subtotal (95% CI)**		**78**		**93**	**23.4%**	**1.15 [0.40, 3.29]**
Total events	7		8			
Heterogeneity: Chi² = 0.42, df = 1 (P = 0.52); I² = 0%						
Test for overall effect: Z = 0.26 (P = 0.80)						
**2.1.2 Unknown**						
Xi et al. 35 (2017)	15	132	31	211	76.6%	0.74 [0.39, 1.44]
**Subtotal (95% CI)**		**132**		**211**	**76.6%**	**0.74 [0.39, 1.44]**
Total events	15		31			
Heterogeneity: Not applicable						
Test for overall effect: Z = 0.88 (P = 0.38)						
**Total (95% CI)**		**210**		**304**	**100.0%**	**0.84 [0.48, 1.46]**
Total events	22		39			
Heterogeneity: Chi² = 0.84, df = 2 (P = 0.66); I² = 0%						
Test for overall effect: Z = 0.62 (P = 0.54)						
Test for subgroup differences: Chi² = 0.47, df = 1 (P = 0.49), I² = 0%						

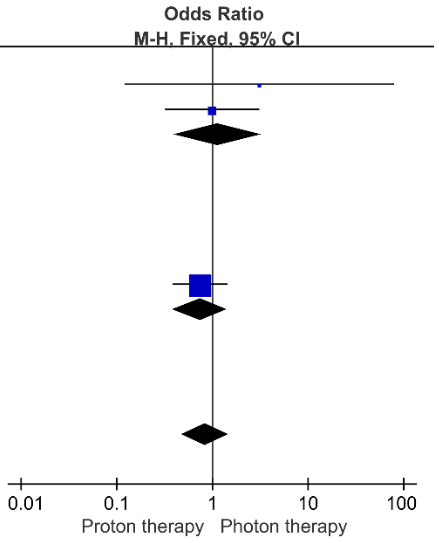

f) G3+ RP

Study or Subgroup	Proton therapy Events	Proton therapy Total	Photon therapy Events	Photon therapy Total	Weight	Odds Ratio M-H, Fixed, 95% CI
**2.2.1 Acute**						
Lin et al. 28 (2020)	0	46	1	61	17.1%	0.43 [0.02, 10.89]
Suh et al. 25 (2021)	0	48	0	29		Not estimable
**Subtotal (95% CI)**		**94**		**90**	**17.1%**	**0.43 [0.02, 10.89]**
Total events	0		1			
Heterogeneity: Not applicable						
Test for overall effect: Z = 0.51 (P = 0.61)						
**2.2.2 Late**						
Makishima et al. 38 2015	0	25	1	19	22.2%	0.24 [0.01, 6.28]
Sumiya et al. 27 (2021)	0	54	0	15		Not estimable
**Subtotal (95% CI)**		**79**		**34**	**22.2%**	**0.24 [0.01, 6.28]**
Total events	0		1			
Heterogeneity: Not applicable						
Test for overall effect: Z = 0.85 (P = 0.39)						
**2.2.3 Unknown**						
Xi et al. 35 (2017)	2	132	6	211	60.7%	0.53 [0.10, 2.64]
**Subtotal (95% CI)**		**132**		**211**	**60.7%**	**0.53 [0.10, 2.64]**
Total events	2		6			
Heterogeneity: Not applicable						
Test for overall effect: Z = 0.78 (P = 0.44)						
**Total (95% CI)**		**305**		**335**	**100.0%**	**0.45 [0.12, 1.67]**
Total events	2		8			
Heterogeneity: Chi² = 0.18, df = 2 (P = 0.92); I² = 0%						
Test for overall effect: Z = 1.20 (P = 0.23)						
Test for subgroup differences: Chi² = 0.18, df = 2 (P = 0.92), I² = 0%						

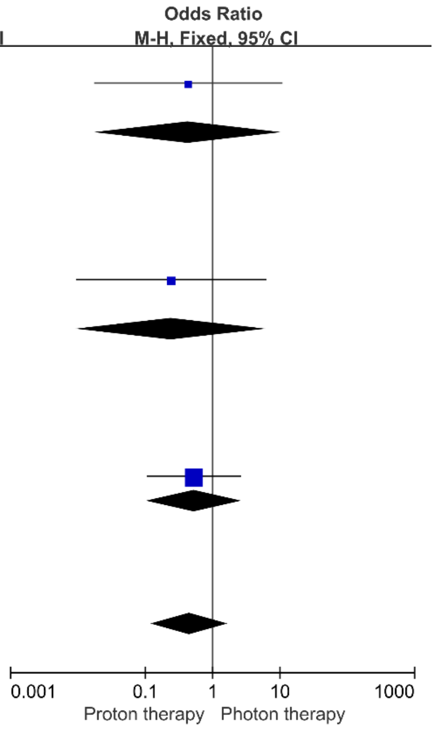

g) G3+ PE

Study or Subgroup	Proton therapy Events	Proton therapy Total	Photon therapy Events	Photon therapy Total	Weight	Odds Ratio M-H, Fixed, 95% CI
**2.3.1 Acute**						
Lin et al. 28 (2020)	0	46	3	61	38.8%	0.18 [0.01, 3.57]
Suh et al. 25 (2021)	0	48	0	29		Not estimable
**Subtotal (95% CI)**		**94**		**90**	**38.8%**	**0.18 [0.01, 3.57]**
Total events	0		3			
Heterogeneity: Not applicable						
Test for overall effect: Z = 1.13 (P = 0.26)						
**2.3.2 Late**						
Makishima et al. 38 2015	0	25	1	19	21.6%	0.24 [0.01, 6.28]
Sumiya et al. 27 (2021)	0	54	0	15		Not estimable
**Subtotal (95% CI)**		**79**		**34**	**21.6%**	**0.24 [0.01, 6.28]**
Total events	0		1			
Heterogeneity: Not applicable						
Test for overall effect: Z = 0.85 (P = 0.39)						
**2.3.3 Unknown**						
Xi et al. 35 (2017)	1	132	4	211	39.7%	0.40 [0.04, 3.57]
**Subtotal (95% CI)**		**132**		**211**	**39.7%**	**0.40 [0.04, 3.57]**
Total events	1		4			
Heterogeneity: Not applicable						
Test for overall effect: Z = 0.83 (P = 0.41)						
**Total (95% CI)**		**305**		**335**	**100.0%**	**0.28 [0.06, 1.32]**
Total events	1		8			
Heterogeneity: Chi² = 0.19, df = 2 (P = 0.91); I² = 0%						
Test for overall effect: Z = 1.61 (P = 0.11)						
Test for subgroup differences: Chi² = 0.19, df = 2 (P = 0.91), I² = 0%						

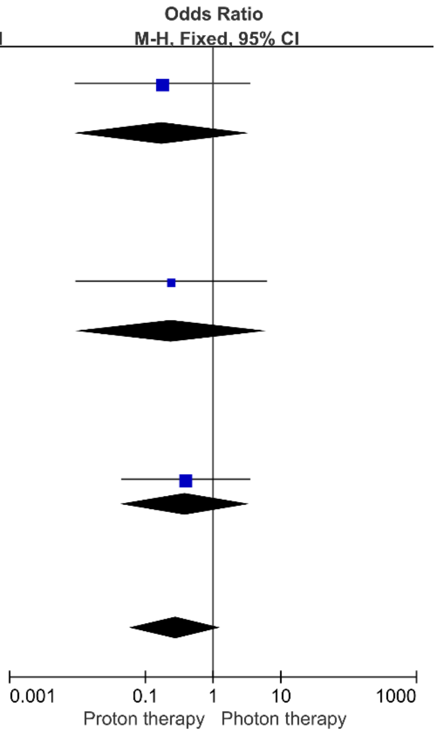

© 2023 Zhou P et al. *JAMA Network Open.*

h) G3+ PCE

Study or Subgroup	Proton therapy Events	Proton therapy Total	Photon therapy Events	Photon therapy Total	Weight	Odds Ratio M-H, Fixed, 95% CI
**2.4.1 Acute**						
Lin et al. 28 (2020)	0	46	1	61	25.1%	0.43 [0.02, 10.89]
Suh et al. 25 (2021)	0	48	0	29		Not estimable
**Subtotal (95% CI)**		**94**		**90**	**25.1%**	**0.43 [0.02, 10.89]**
Total events	0		1			
Heterogeneity: Not applicable						
Test for overall effect: Z = 0.51 (P = 0.61)						
**2.4.2 Late**						
Makishima et al. 38 2015	0	25	0	19		Not estimable
Sumiya et al. 27 (2021)	0	54	0	15		Not estimable
**Subtotal (95% CI)**		**79**		**34**		**Not estimable**
Total events	0		0			
Heterogeneity: Not applicable						
Test for overall effect: Not applicable						
**2.4.3 Unknown**						
Xi et al. 35 (2017)	1	132	5	211	74.9%	0.31 [0.04, 2.72]
**Subtotal (95% CI)**		**132**		**211**	**74.9%**	**0.31 [0.04, 2.72]**
Total events	1		5			
Heterogeneity: Not applicable						
Test for overall effect: Z = 1.05 (P = 0.29)						
**Total (95% CI)**		**305**		**335**	**100.0%**	**0.34 [0.06, 2.06]**
Total events	1		6			
Heterogeneity: Chi² = 0.03, df = 1 (P = 0.87); I² = 0%						
Test for overall effect: Z = 1.17 (P = 0.24)						
Test for subgroup differences: Chi² = 0.03, df = 1 (P = 0.87), I² = 0%						

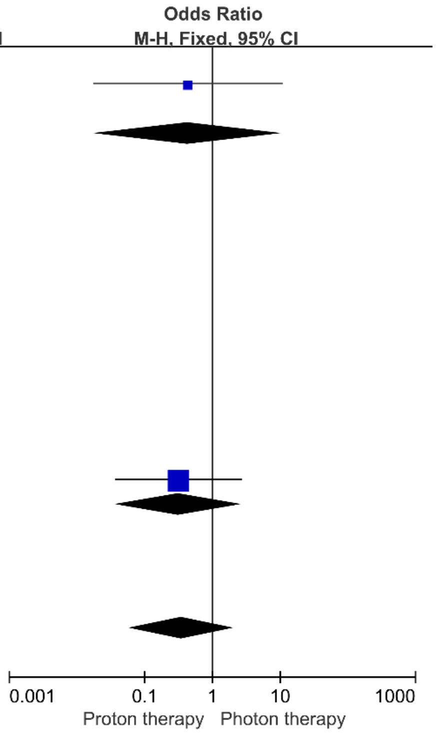

i) G4+ lymphocytopenia

Study or Subgroup	Proton therapy Events	Proton therapy Total	Photon therapy Events	Photon therapy Total	Weight	Odds Ratio M-H, Fixed, 95% CI
**3.1.1 Acute**						
Bhangoo et al. 31 (2020)	6	32	9	32	2.9%	0.59 [0.18, 1.91]
Choi et al. 49 (2022)	2	15	3	16	1.0%	0.67 [0.10, 4.67]
Fang et al. 36 (2018)	34	110	52	110	14.2%	0.50 [0.29, 0.87]
Lin et al. 28 (2020)	12	46	32	61	8.0%	0.32 [0.14, 0.73]
Routman et al. 32 (2019)	12	50	30	50	9.0%	0.21 [0.09, 0.50]
Shiraishi et al. 33 2018	24	136	55	136	17.9%	0.32 [0.18, 0.55]
Zhu et al. 46 (2021)	54	246	231	500	47.0%	0.33 [0.23, 0.46]
**Subtotal (95% CI)**		**635**		**905**	**100.0%**	**0.35 [0.28, 0.44]**
Total events	144		412			
Heterogeneity: Chi² = 4.42, df = 6 (P = 0.62); I² = 0%						
Test for overall effect: Z = 8.90 (P < 0.00001)						
**Total (95% CI)**		**635**		**905**	**100.0%**	**0.35 [0.28, 0.44]**
Total events	144		412			
Heterogeneity: Chi² = 4.42, df = 6 (P = 0.62); I² = 0%						
Test for overall effect: Z = 8.90 (P < 0.00001)						
Test for subgroup differences: Not applicable						

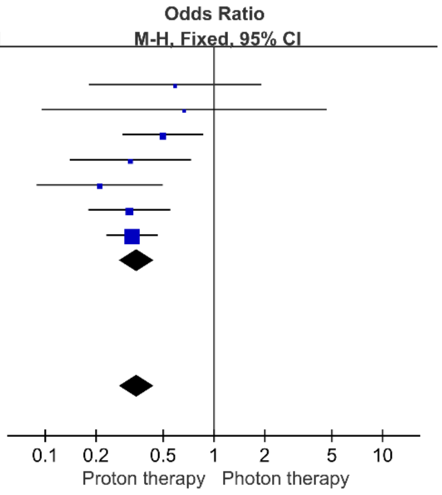

© 2023 Zhou P et al. *JAMA Network Open.*

eFigure 5. Example of Single-Rate Pooled Analysis of OS and PFS for Proton Therapy

A/B: 2y-OS; C/D: 2y-PFS; A/C: sub-group by treatment modalities (neoadjuvant, racial, or mixed); B/D: sub-group by stage (III-IV < 50% or ≥ 50%).

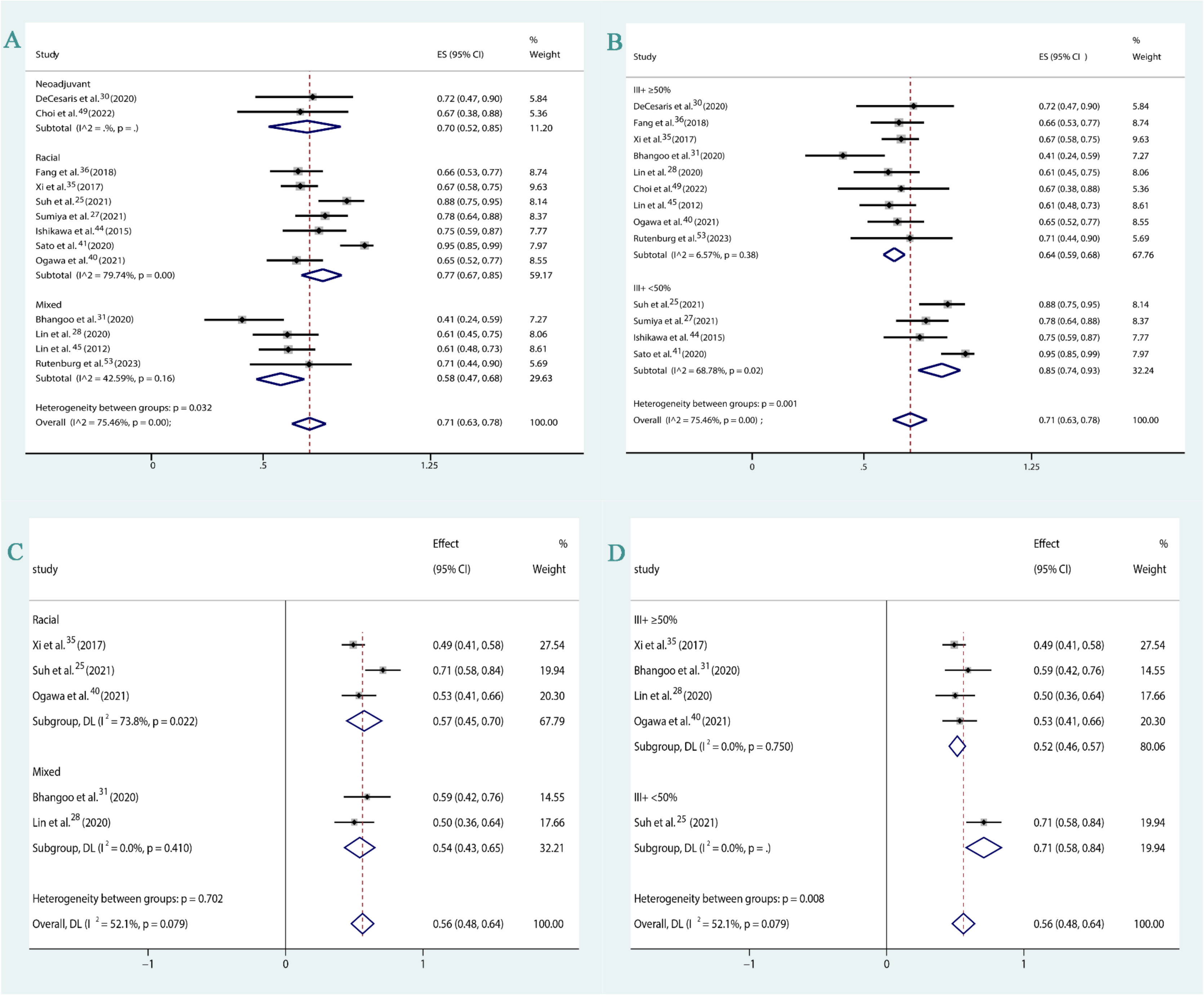

eFigure 6. Single-Rate Pooled Analysis of CR (Proton Therapy)

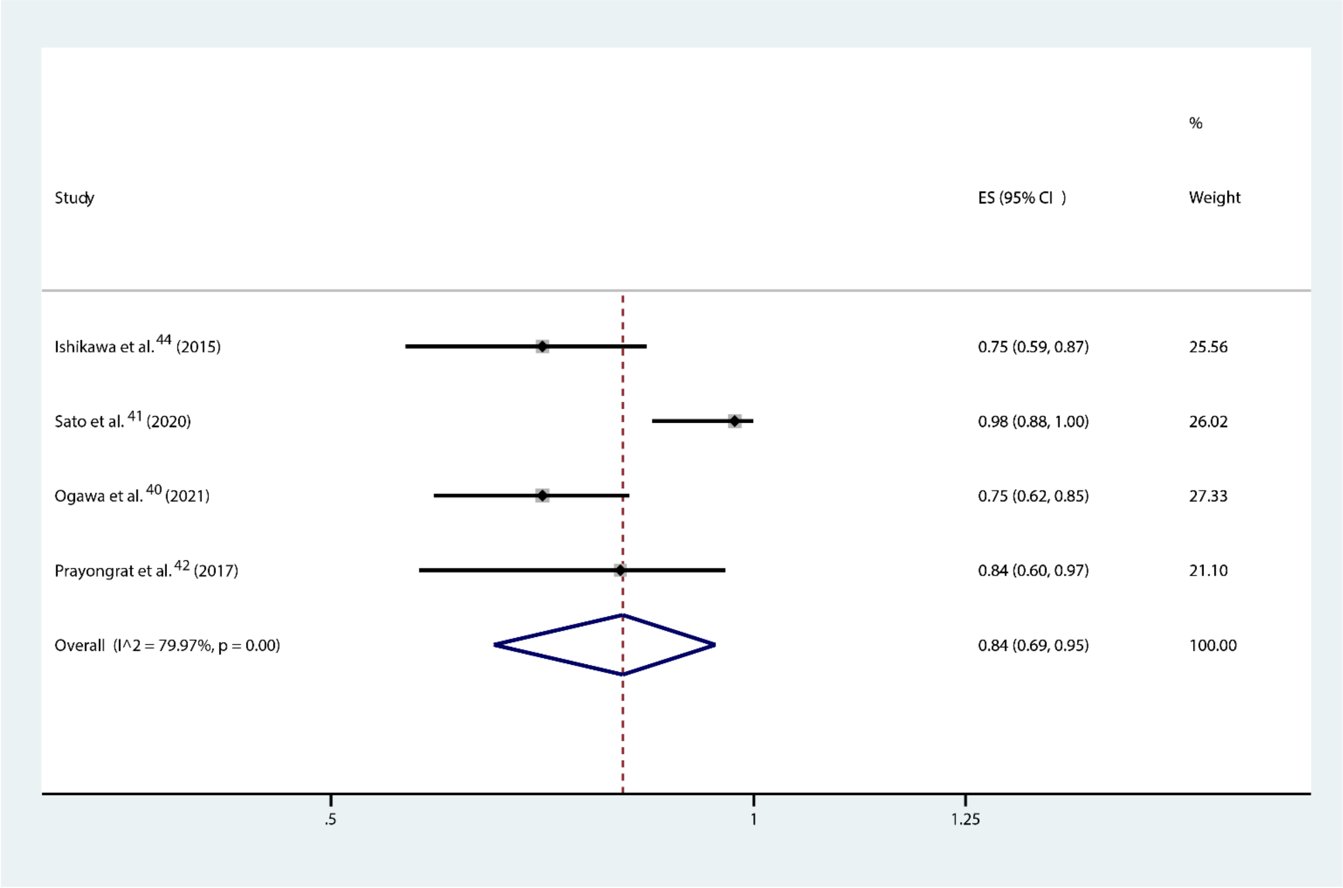

© 2023 Zhou P et al. *JAMA Network Open.*

eFigure 7. Single-Rate Pooled Analysis of pCR (Proton Therapy)

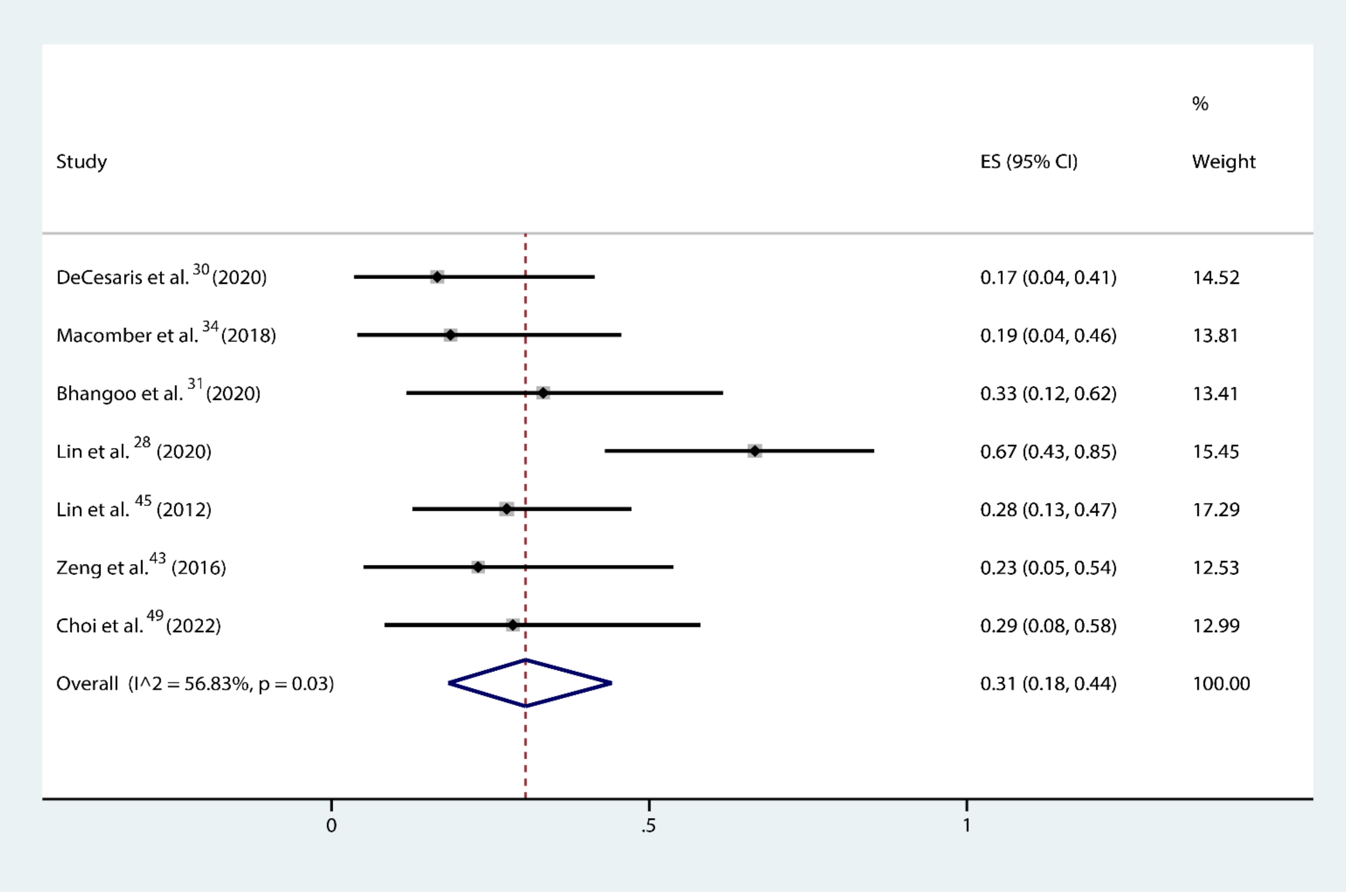

© 2023 Zhou P et al. *JAMA Network Open.*

eTable 1. Detailed Comparison Information on OARs Dosimetric and Planning Quality From Proton and Photon Radiotherapy Planning

Reference	Patients	Characteristics	Dose limitations	OARs dose (% diff. between means)	Plans quality
Ribeiro et al.[10]<br>2021	10 (20)	PBT (IMPT) vs. VMAT (4DCT)<br>PTV: 41.4 Gy/23F<br>Site: L (80%)	-	Lung: *D_{mean}↓: 3.8Gy.<br>Heart: *D_{mean}↓: 9.5Gy.	No SS change
Zhang et al.[11]<br>2021	25	PBT (PSPT) vs. IMRT/ VMAT (4DCT)<br>PTV: 50.4 Gy/28F<br>Site: -	Lung: D_{mean}<20Gy, V_5<50%, V_{10}<40%, V_{20}<20%;<br>Heart: D_{mean}<20Gy;<br>VB: D_{mean}<18.8Gy, V_{10}<54.2% (<260 cc), V_{20}<44.3% (<225 cc).	# (vs. IMRT) Lung: *D_{mean}↓: 3.0Gy, *$V_{5,\ 10}$↓: 25.3%, 12.9%, V_{20}↓: 4.8%. Heart: *D_{mean}↓: 5.6Gy. SC: *D_{max}↓: 2.4Gy.<br>VB: *D_{mean}↓: 4.9Gy, *$V_{10,\ 20}$↓: 18.5%, 12.6%.<br># (vs. VMAT) Lung: *D_{mean}↓: 3.6Gy, *$V_{5,\ 10,\ 20}$↓: 31.7%, 20.4%, 3.7%.<br>Heart: D_{mean}↓: 2.6Gy. SC: D_{max}↑: 4.1Gy.<br>VB: *D_{mean}↓: 2.8Gy, *$V_{10,\ 20}$↓: 14.9%, 8.2%.	No SS change
Anakotta et al.[12]<br>2020	19 (20)	PBT (IMPT) vs. VMAT (4DCT)<br>PTV: 41.4-51Gy/17-28F<br>Site: L (18)	Lung: D_{mean}⩽20Gy, V_5⩽70%, V_{20}⩽ 30%;<br>Heart: D_{mean}⩽26Gy.	Lung: *D_{mean}↓: 5.0Gy, *$V_{5,\ 20}$↓: 32.6%, 5.6%.<br>Heart: *D_{mean}↓: 11.8Gy, *V_5↓: 56.3%.<br>SC: *D_{max}↓: 6.2Gy.	No SS change
Celik et al.[13]<br>2020	20	PBT (IMPT) vs. VMAT<br>PTV: 41.4Gy/23F<br>Site: L	Lung: D_{mean}⩽12Gy, V_{20}⩽20%;<br>Heart: D_{mean}⩽10Gy, V_{30}⩽10%.	Lung: *D_{mean}↓: 5.7Gy, *V_{20}↓: 4.6%.<br>Heart: *D_{mean}↓: 6.2Gy, *V_{30}↓: 1.0%.<br>LV/ LCA/ LAD: *D_{mean}↓: 4.6/ 3.3/ 3.0Gy.	SS HI ↓; no SS CI
Liu et al.[14]<br>2019	19 vs. 16	PBT (IMPT) vs. VMAT (4DCT)<br>PTV: 41.4-50.4 Gy<br>Site: -	Lung: D_{mean}<15Gy, V_5<60%, V_{20}<15%;<br>Heart: D_{mean}<26Gy, V_{25}<50%, V_{40}<30%.	^Lung: *D_{mean}↓: 4.8Gy, *V_5↓: 32.5%, V_{20}↓: 1.8%. (med)<br>^Heart: *D_{mean}↓: 14.3Gy, *V_{20}↓: 35.5%, $V_{30,\ 40}$↓: 7.0%, 2.1%.<br>^SC: D_{max}↑: 2.9Gy.	No SS change
Møller et al.[15]<br>2019	26	PBT (SFUD) vs. IMRT<br>PTV: 41.4-50Gy/23-25F<br>Site: M/L (5/21)	Lung: D_{mean}<19Gy, V_5<60%, V_{20}<35%;<br>Heart: V_{25}<40%, V_{40}<30%.	#Lung: *D_{mean}↓: 6.6Gy, *$V_{5,\ 20}$↓: 41.1%, 8.2%.<br>#Heart: *D_{mean}↓: 7.9Gy, V_{25}↓: 5.7%, V_{40}↑: 0.2%.	No SS change
Hirano et al.[16]<br>2018	27 (37)	PBT vs. IMRT/ 3D-CRT<br>PTV: 60Gy/30F<br>Site: U/M/ L (5/9/13)	PBT: Lung: V_{20}<20%; Heart: D_{mean}<26Gy, V_{30}<46%.<br>IMRT: Lung: V_{20}<30%; Heart: D_{mean}<50Gy.<br>3D-CRT: -	(vs. IMRT) Lung: *D_{mean}↓: 3.6Gy, *$V_{5,\ 10,\ 15,\ 20}$↓: 19.9%, 8.7%, 5.1%, 6.1%. SC: D_{max}↓: 1.6Gy.<br>Heart: *D_{mean}↑: 8.1Gy, *$V_{20,\ 30,\ 40}$↓: 30.8%, 28.7%, 10.3%.<br>(vs. 3D-CRT) Lung: *D_{mean}↓: 2.4Gy, *$V_{5,\ 20}$↓: 7.8%, 5.3%; $V_{10,\ 15}$↓: 4.4%, 3.8%. SC: *D_{max}↓: 9.1Gy.<br>Heart: *D_{mean}↓: 13.4Gy, *$V_{20,\ 30,\ 40}$↓: 25.6%, 34.1%, 31.9%.	(vs. IMRT) SS CI ↑;<br>(vs. 3D-CRT) SS CI↓
Shiraishi et al.[17]<br>2017	250 vs. 477	PBT (95% PSPT) vs. IMRT (4DCT)<br>PTV: 50.4Gy/28F<br>Site: L (223 vs. 428)	Lung: D_{mean}⩽20Gy, V_5<55%, V_{20}⩽ 35%;<br>Heart: D_{mean}<26Gy, V_{30}⩽45%.	Heart: *D_{mean}↓: 10.4Gy, *$V_{5,\ 10,\ 20,\ 30,\ 40}$↓: 49.6%, 42.4%, 23.0%, 10.8%, 3.5%.<br>LA/ LV: *D_{mean}↓: 4.0/ 9.5Gy; RA/ RV: *D_{mean}↓: 10.0/ 14.8 Gy.<br>LMC/ LAD/ LCX/ RCA: *D_{mean}↓: 16.8/ 11.1/ 3.5/ 15.1 Gy.	No SS change
Warren et al.[18]<br>2017	21	PBT vs. VMAT<br>PTV: 62.5 Gy/25F<br>Site: M	TV: -	#TV: *D_{mean}↓: 3.3Gy, *$V_{10,\ 20}$↓: 4%, 10.8%.	No SS change
Warren et al.[19]<br>2016	21	PBT vs. VMAT<br>PTV: 62.5 Gy/25F<br>Site: M	Lung: D_{mean}<20Gy, V_{20}<25%;<br>Heart: D_{mean}<25Gy, V_{30}<45%.	Lung: *D_{mean}↓: 6.8Gy, *V_{20}↓: 8.8%.<br>Heart: *D_{mean}↓: 8.5Gy.	No SS change
Ling et al.[20]<br>2014	10	PBT vs. 3DCRT/IMRT<br>PTV: 50.4Gy/28F<br>Site: L	Lung: V_{20}<30%;<br>Heart: V_{40}<30%.	(vs. IMRT) Lung: *D_{mean}↓: 3.5Gy, *$V_{5,\ 10,\ 15}$↓: 25.5%, 18.4%, 10.6%, $V_{20,\ 30,\ 50}$↓: 0.9%, 0.5%, 0.5%, V_{40}↑: 0.8%.<br>Heart: *D_{mean}↓: 15.9Gy, *$V_{25,\ 30,\ 40,\ 50}$↓: 30.4%, 21.4%, 9.3%, 9.2%.<br>LAD/ LV/ PC: *D_{mean}↓: 17.2/ 16.4/ 12.5Gy.<br>SC: *D_{max}↓: 25.3Gy.<br>(vs. 3D-CRT) Lung: *D_{mean}↓: 3.4Gy, *$V_{5,\ 50}$↓: 12.7%, 2.2%; $V_{10,\ 15,\ 20,\ 30,\ 40}$↓: 9.7%, 6.6%, 6.8%, 3.7%, 0.4%.<br>SC: *D_{max}↓: 19.6Gy.<br>Heart: *D_{mean}↓: 14.9Gy, *$V_{25,\ 30,\ 40,\ 50}$↓: 32.6%, 11.8%, 9.6%, 17.2%.<br>LAD/ LV/ PC: *D_{mean}↓: 14.7/ 13.9/ 11.3Gy.	(vs. IMRT) no SS CI, HI<br>(vs. 3D-CRT) no SS CI, SS HI ↑
Welsh et al.[21]<br>2011	10	PBT (IMPT) vs. IMRT (4DCT)<br>PTV: 50.4Gy/28F<br>Site: -	-	Lung: *D_{mean}↓: 3.4Gy, *$V_{5,\ 10}$↓: 19%, 8%, V_{20}↓: 3%.<br>Heart: *D_{mean}↓: 9.3Gy, *$V_{10,\ 20,\ 30}$↓: 50%, 22%, 8%, $V_{40,\ 50}$↓: 2%, 0%.<br>SC: D_{max}↑: 0.1Gy.	-
Zhang et al.[22]<br>2008	15	PBT (PSPT) vs. IMRT (4DCT)<br>PTV: 50.4 Gy/25F<br>Site: L	-	#Lung: *D_{mean}↓: 3.0Gy, *$V_{5,\ 10,\ 20,\ 30}$↓: 17.4%, 8.4%, 5.0%, 0.5%.<br>#SC: D_{max}↓: 15.4Gy.<br>#Heart: *V_{40}↓: 8.0%; *V_{50}↑: 0.9%.	SS CI ↑
Mu et al.[23]<br>2011	10	PBT vs. IMRT<br>PTV: 60Gy/30F<br>Site: U	Lung: V_5<42%, V_{10}<35%; V_{20}<20%;	Lung: *D_{mean}↓: 4.8Gy, *$V_{5,\ 20}$↓: 31.4%, 6.1%.<br>SC: D_{max}↓: 6.5Gy.	SS CI ↑
Wang et al.[24]<br>2015	55	PBT (PSPT) vs. IMRT<br>PTV: 50.4Gy/28F<br>Site: -	Lung: D_{mean}⩽20Gy, V_{20}⩽35%/ 30%;<br>Heart: D_{mean}<26Gy, V_{30}⩽45%.	Lung: *D_{mean}↓: 2.9Gy, *$V_{5,\ 10,\ 20}$↓: 20.3%, 9.7%, 4.2%.<br>Heart: *$V_{10,\ 20,\ 30}$↓: 32.7%, 13.3%, 2.5%.<br>SC: D_{max}↓: 8.5Gy.	No SS change
Suh et al.[25]<br>2021	48 vs. 29	PBT vs. 3D-CRT/IMRT (24/5)<br>PTV: 66Gy/33F (med)<br>*Site: L (58% vs. 28%)	-	# ^(vs. photon) Lung: *D_{mean}↓: 4.8Gy, *$V_{5,\ 10,\ 20,\ 30}$↓: 26.1%, 14.6%, 3.5%.<br>Heart: *D_{mean}↓: 17.7Gy, *$V_{5,\ 10,\ 20,\ 30}$↓: 12.7%, 12.7%, 46%, 43.3%.	-
Sumiya et al.[27]<br>2021	54 vs. 15	PBT vs. 3D-CRT<br>PTV: 56-70Gy/28-35F<br>Site: M+L (80% vs. 67%)	-	WTB: *$V_{5,\ 10,\ 20,\ 30,\ 40,\ 50}$↓: 10.1%, 8.6%, 16.5%, 26.6%, 19%, 5.3%, V_{60}↓: 0.4%.	-
Lin et al.[28]<br>2020	46 vs. 61	PBT (80% PSPT) vs. IMRT<br>PTV: 50.4Gy/28F	Lung: D_{mean}<20Gy, V_5<60%, V_{10}<40%, V_{20}<35%;	#Lung: *D_{mean}↓: 3.6Gy, *$V_{5,\ 20}$↓: 21.7%, 5.2%.<br>#Heart: *D_{mean}↓: 8.5Gy.	PTV coverage SS ↑

© 2023 Zhou P et al. *JAMA Network Open.*

		Site: L (83% vs. 84%)	Heart: D_{mean}<26Gy, V_{30}<45%.	#SC: D_{max}↓: 0.1Gy.	
Wang et al.[29]<br>2020	159 vs. 320	PBT (91% PSPT) vs. IMRT<br>PTV: 50.4Gy/28F (med)<br>Site: L (138 vs. 288)	-	Heart: *D_{mean}↓: 9.1Gy, *$V_{5, 30}$↓: 45.4%, 8.1%.	-
Bhangoo et al.[31]<br>2020	32 vs. 32	PBT (IMPT) vs. IMRT (4DCT)<br>PTV: 50Gy/25F (med)<br>Site: L (78% vs. 94%)	Lung: D_{mean}<15Gy, V_{20}<20%;<br>Heart: D_{mean}<26Gy, V_{40}<30%	#Lung: *D_{mean}↓: 5.3Gy, V_{20}↓: 2%.<br>#Heart: *D_{mean}↓: 11.2Gy, *V_{30}↓: 7%.	-
Macomber et al.[34]<br>2018	16 vs. 39	PBT vs. IMRT/3D-CRT (21/18)<br>PTV: 50.4Gy/28F<br>Site: L (82%)	-	(vs. IMRT) Heart: *D_{mean}↓: 16.1Gy, *$V_{5, 40}$↓: 66%, 6%, V_{50}↓: 1%.<br>(vs. 3D-CRT) Heart: *D_{mean}↓: 25.1Gy, *$V_{5, 40, 50}$↓: 64%, 27%, 6%.	-
Xi et al.[35]<br>2017	132 vs. 211	PBT vs. IMRT<br>PTV: 50.4Gy/28F (med)<br>Site: L (71% vs. 73%)	Lung: D_{mean}<20Gy, V_{20}<35%;<br>Heart: V_{40}<40%.	Lung: *D_{mean}↓: 3.5Gy, *$V_{5, 10, 20}$↓: 19.7%, 9.1%, 7.1%.<br>Heart: *D_{mean}↓: 8.3Gy, *V_{30}↓: 5.5%, V_{40}↓: 0.3%.	PTV coverage SS ↑
Lin et al.[37]<br>2017	111 vs. 469	PBT vs. 3D-CRT/IMRT (214/ 255)<br>PTV: 50.4Gy/28F<br>*Site: L (98% vs. 88%/95%)	Lung: D_{mean}<20Gy, V_{20}<35%;<br>Heart: V_{40}<40%.	(vs. 3D-CRT) Lung: *D_{mean}↓: 4.4Gy;<br>Heart: *D_{mean}↓: 15.2Gy.<br>(vs. IMRT) Lung: *D_{mean}↓: 3.4Gy;<br>Heart: *D_{mean}↓: 9.2Gy.	-
Makishima et al.[38]<br>2015	44<br>(25 vs. 19)	PBT (IMPT) vs. 3D-CRT<br>PTV: 60Gy/30F (med)<br>Site: M (88%)	-	# (25 vs. 25) Lung: *D_{mean}↓: 3.6Gy, *$V_{5, 10, 20}$↓: 14.7%, 10%, 7.0%.<br>Heart: *$V_{30, 40, 50}$↓: 41.8%, 36.5%, 23.5%.<br># (19 vs. 19) Lung: *D_{mean}↓: 8.0Gy *$V_{5, 10, 20}$↓: 26.7%, 17.9%, 13.4%.<br>Heart: *$V_{30, 40, 50}$↓: 46.2%, 46.1%, 13.5%.	-
Zhu et al.[46]<br>2021	246 vs. 500	PBT vs. PRT<br>PTV: 50.4Gy<br>Site: L (88% vs. 86%)	-	(vs. photon) Lung: *D_{mean}↓: 4.1Gy, *$V_{5\text{-}40}$↓: 21.9%, 10.1%, 7.7%, 7.6%, 2.8%, 1.43%, 0.72%. $V_{45, 50}$: 0.2%, 0%.<br>Heart: *D_{mean}↓: 10.4Gy, *$V_{5\text{-}45}$↓: 48.9%, 42.5%, 32%, 23.4%, 16.2%, 10.6%, 6.3%, 3.4%, 1.5%; V_{50} ↓: 0.6%.	-
Oonsiri et al.[47]<br>2022	25	PBT (IMPT) vs. IMRT<br>PTV: 60-66Gy/ F<br>Site: U+M (76%)	-	Lung: *Dmean↓: 6.8Gy, *$V_{5, 10, 15, 20, 30}$↓: 30%, 27.4%, 18.6%, 11.3%, 3.8%, V_{40}↓: 0.5%;<br>Heart: *Dmean↓: 6.5Gy. *$V_{10, 20, 30, 40, 50}$↓: 43.6%, 40.4%, 28.4%, 15.8%, 7.3%.	No SS change
Choi et al.[49]<br>2022	15 vs. 16	PBT vs. PRT<br>PTV: 41.4Gy/28F<br>Site: M+L (73% vs. 69%)	-	Lung: *Dmean↓: 3.9Gy, *$V_{10, 20}$↓: 9.4%, 4.2%;<br>Heart: *Dmean↓: 13.6Gy. *$V_{10, 30, 40}$↓: 25.4%, 33.5%, 22.9%.	-
Kato et al.[50]<br>2022	10	PBT (IMPT) vs. VMAT<br>PTV: 60Gy/30F<br>Site: U	Lung: V_{20}<30%, V_{30}<20%;	Lung: *Dmean↓: 3.0Gy, *$V_{5, 10, 20}$↓: 12.8%, 11.0%, 5.3%, $V_{30, 40}$↓: 1.5%, 0.4%.	no SS CI, SS HI ↓
Zhang et al.[51]<br>2022	11	PBT (IMPT) vs. IMRT<br>PTV: 50.4Gy/28F<br>-	Lung: D_{mean}≤15Gy, V_{20}≤30%, V_{30}≤30%;<br>Heart: D_{mean}≤30Gy, V_{30}≤40%, V_{40}≤30%.	Lung: Dmean↓: 8.4Gy, $V_{5, 10, 15, 20}$↓: 40.8%, 26.6%, 19.9%, 15.4%.<br>Heart: Dmean↓: 11.6Gy. *$V_{10, 20}$↓: 30.9%, 32.9%.<br>(No statistical tests).	-
Cui et al.[52]<br>2022	30	PBT (IMPT) vs. IMRT/VMAT<br>PTV: 60Gy/30F<br>-	Lung: V_5<65%, V_{20}<25%;<br>Heart: D_{mean}<26Gy, V_{30}<46%.	(vs. IMRT) Lung: *Dmean↓: 8.4Gy, *$V_{5, 10, 15, 20}$↓: 40.8%, 26.6%, 19.9%, 15.4%. Heart: *Dmean↓: 11.6Gy. *$V_{10, 20}$↓: 30.9%, 32.9%, $V_{5, 30, 40}$↓: 29.5%, 20.9%, 9.9%. SC: Dmax↓: 4.7Gy.<br>(vs. VMAT) Lung: *Dmean↓: 7.3Gy, *$V_{5, 10, 15}$↓: 43.8%, 24.0%, 14.4%, V_{20}↓: 8.8%. Heart: *Dmean↓: 14.6Gy, *$V_{20, 30, 40}$↓: 34.1%, 28.3%, 21.33%, $V_{5, 10}$↓: 27.2%, 27.9%. SC: *Dmax↓: 8.9Gy.	(vs. IMRT) no SS CI, HI<br>(vs. VMAT) SS CI, HI ↑

Key: All represent as mean ± SD (except special explain). #: represent as median; ^: represent obtained from picture; *: represent statistically significant; SS: statistically significantly; OARs: organs at risk; PTV: planning target volume; F: fraction; U/M/L: upper/ middle/ lower thoracic; D_{mean}: mean dose; V_x: volume receiving ≥x Gy; PBT: proton beam therapy; PRT: photon radiotherapy; SFUD: single-field uniform dose; IMPT: intensity modulated proton therapy ; PSPT: passive scattering proton therapy; IMRT: intensity modulated proton therapy; CTV: clinical target volume; SC: spinal cord; LA(RA): left (right) atrium; LV(RV): left (right) ventricle; LCA: Left coronary artery; LMC: left main coronary artery; LAD: left anterior descending artery; LCX: left circumflex; RCA: right coronary artery; PC: Pericardium; HI: homogeneity index; CI: conformity index; VB: Vertebral Body (outline of the vertebrae, C2-L1); TV: thoracic vertebrae body (T1-T12); WTB: including the vertebrae of C5 to T12, all ribs, and the whole sternum.

© 2023 Zhou P et al. *JAMA Network Open.*

eTable 2. Results of Quality Assessment Using the Newcastle-Ottawa Scale for Non-RCT Studies (Dosimetric Parameters)

Reference	Representativeness of the expose cohort	Selection of the non-exposed cohort	Ascertainment of exposure	Demonstration that outcome of the interest was not present at start of study	Comparability of Cohorts on the basis of the design or analysis	Assessment of outcome	Was follow-up long enough for outcome to occur	Adequacy of follow up of cohorts	Total score
Zhang et al.[11] 2021	☆	☆	☆	☆	☆☆	☆	☆	☆	9
Anakotta et al.[12] 2020	☆	☆	☆	☆	☆☆	☆	☆	☆	9
Celik et al.[13] 2020	☆	☆	☆	☆	☆☆	☆	☆	☆	9
Liu et al.[14] 2019	☆	☆	☆	☆	☆	☆	☆	☆	8
Møller et al.[15] 2019	☆	☆	☆	☆	☆☆	☆	☆	☆	9
Hirano et al.[16] 2018	☆	☆	☆	☆	☆	☆	☆	☆	8
Shiraishi et al.[17] 2017	☆	☆	☆	☆	☆	☆	☆	☆	8
Warren et al.[18] 2017	☆	☆	☆	☆	☆	☆	☆	☆	8
Warren et al.[19] 2016	☆	☆	☆	☆	☆☆	☆	☆	☆	9
Ling et al.[20] 2014	☆	☆	☆	☆	☆☆	☆	☆	☆	9
Welsh et al.[21] 2011	☆	☆	☆	☆	☆	☆	☆	☆	8
Zhang et al.[22] 2008	☆	☆	☆	☆	☆	☆	☆	☆	8
Mu et al.[23] 2011	☆	☆	☆	☆	☆☆	☆	☆	☆	9
Sumiya et al.[27] 2021	☆	☆	☆	☆	-	☆	☆	☆	7
Lin et al.[28] 2020	☆	☆	☆	☆	☆	☆	☆	☆	8
Wang et al.[29] 2020	☆	☆	☆	☆	-	☆	☆	☆	7
Bhangoo et al.[31] 2020	☆	☆	☆	☆	☆	☆	☆	☆	8
Xi et al.[35] 2017	☆	☆	☆	☆	☆	☆	☆	☆	8
Lin et al.[37] 2017	☆	☆	☆	☆	☆	☆	☆	☆	8
Kato et al.[50] 2022	☆	☆	☆	☆	☆☆	☆	☆	☆	9
Zhang et al.[51] 2022	☆	☆	☆	☆	☆☆	☆	☆	☆	9
Cui et al.[52] 2022	☆	☆	☆	☆	☆☆	☆	☆	☆	9

© 2023 Zhou P et al. *JAMA Network Open.*

eTable 3. Results of Quality Assessment Using the Newcastle-Ottawa Scale for Non-RCT Studies (Efficacy and Safety of Proton vs Photon Therapy)

Reference	Represe-ntativeness of the expose cohort	Selection of the non-exposed cohort	Ascertain-ment of exposure	Demonstration that outcome of the interest was not present at start of study	Comparability of Cohorts on the basis of the design or analysis	Assessment of outcome	Was follow-up long enough for outcome to occur	Adequacy of follow up of cohorts	Total score
Suh et al.[25] 2021	☆	☆	☆	☆	☆	☆	☆	☆	8
Sumiya et al.[27] 2021	☆	☆	☆	☆	-	☆	-	☆	6
DeCesaris et al.[30] 2020	☆	☆	☆	☆	-	☆	-	☆	6
Bhangoo et al.[31] 2020	☆	☆	☆	☆	☆	☆	-	☆	7
Routman et al.[32] 2019	☆	☆	☆	☆	-	☆	☆	☆	7
Shiraishi et al.[33] 2018	☆	☆	☆	☆	☆	☆	☆	☆	8
Macomber et al.[34] 2018	☆	☆	☆	☆	☆	☆	-	☆	7
Xi et al.[35] 2017	☆	☆	☆	☆	☆	☆	☆	☆	8
Fang et al.[36] 2018	☆	☆	☆	☆	☆	☆	☆	☆	8
Makishima et al.[38] 2015	☆	☆	☆	☆	-	☆	☆	☆	7
Zhu et al.[46] 2021	☆	☆	☆	☆	☆	☆	☆	☆	8
Choi et al.[49] 2022	☆	☆	☆	☆	☆	☆	-	☆	7

eTable 4. Results of Quality Assessment Using the Cochrane Collaboration's Tool (RCT)

Reference	Random sequence generation	Allocation concealment	Blinding	Incomplete outcome data	Selective reporting	Other bias
Lin et al.[28] 2020	Low	Unclear	High	Low	Low	Unclear

© 2023 Zhou P et al. *JAMA Network Open.*

eTable 5. Results of Quality Assessment Using the MINORS (Single-Group Tests)

Reference	A clearly stated aim	Inclusion of consecutive patients	Prospective collection of data	Endpoints appropriate to the aim of the study	Unbiased assessment of the study endpoint	Follow-up period appropriate to the aim of the study	Loss to follow up less than 5%	Prospective calculation of the study size	Total score
Echeverria et al.[4] 2013	2	2	2	2	0	2	2	0	12
Hirano et al.[16] 2018	2	2	2	2	0	1	2	0	11
Parzen et al.[39] 2021	2	1	2	2	0	2	2	0	11
Ogawa et al.[40] 2021	2	2	2	2	0	2	2	0	12
Sato et al.[41] 2020	2	2	2	2	0	1	2	0	11
Prayongrat et al.[42] 2017	2	2	2	2	0	1	2	0	11
Zeng et al.[43] 2016	2	2	1	1	0	1	2	0	9
Ishikawa et al.[44] 2015	2	2	2	2	0	2	2	0	12
Lin et al.[45] 2012	2	2	2	2	0	1	2	0	11
Rutenburg et al.[53] 2023	2	2	2	2	0	1	2	0	11

© 2023 Zhou P et al. *JAMA Network Open.*

eTable 6. Pooled Analysis of Dose-Volume Parameters for OARs with Proton vs Photon Therapy

	vs. 3D-CRT	vs. IMRT	vs. VMAT	Overall (Proton vs. Photon)	Egger's test (P value)
Lung					
Dmean (Gy)	-3.46 (-5.03, -1.88)	-4.66 (-5.70, -3.61)	-4.41 (-7.14, -1.68)	-4.42 (-5.38, -3.47)	0.959
	I^2 = 77%	I^2 = 88%	I^2 = 96%	I^2 = 93%	
V_5 (%)	-8.83 (-13.55, -4.11)	-26.89 (-32.19, -21.58)	-25.93 (-47.42, -4.45)	-24.81 (-31.40, -18.21)	0.199
	I^2 = 0%	I^2 = 86%	I^2 = 98%	I^2 = 95%	
V_{10} (%)	-5.35 (-9.38, -1.32)	-12.87 (-17.91, -7.83)	-17.51 (-26.57, -8.45)	-12.92 (-16.84, -9.00)	0.359
	I^2 = 0%	I^2 = 83%	I^2 = 79%	I^2 = 82%	
V_{20} (%)	-5.48 (-8.18, -2.78)	-7.16 (-9.77, -4.55)	-5.55 (-7.71, -3.38)	-6.52 (-8.24, -4.79)	0.511
	I^2 = 0%	I^2 = 89%	I^2 = 64%	I^2 = 84%	
V_{30} (%)	-	-3.56 (-9.24, 2.12)	-	-3.05 (-5.86, -0.25)	*
		I^2 = 95%		I^2 = 89%	
V_{40} (%)	-	-	-	-0.26 (-0.87, 0.34)	*
				I^2 = 0%	
Heart					
D_{mean} (Gy)	-15.08 (-16.36, -13.80)	-8.74 (-10.53, -6.94)	-8.86 (-12.02, -5.69)	-9.52 (-11.11, -7.94)	0.588
	I^2 = 0%	I^2 = 90%	I^2 = 89%	I^2 = 92%	
V_5 (%)	/	-45.29 (-51.22, -39.37)	-42.51 (-70.90, -14.13)	-45.06 (-51.18, -38.94)	*
		I^2 = 81%	I^2 = 92%	I^2 = 82%	
V_{10} (%)	/	-42.36 (-49.92, -34.79)	-	-39.52 (-47.45, -31.60)	*
		I^2 = 66%		I^2 = 72%	
V_{20} (%)	-	-25.36 (-30.17, -20.55)	-	-26.60 (-31.90, -22.12)	*
		I^2 = 41%		I^2 = 43%	
V_{30} (%)	-22.75 (-44.70, -0.81)	-13.07 (-16.67, -9.48)	-10.51 (-22.16, 114)	-13.89 (-17.84, -9.95)	<0.001
	I^2 = 92%	I^2 = 85%	I^2 = 93%	I^2 = 93%	
V_{40} (%)	-20.54 (-42.39, 1.31)	-5.17 (-7.92, -2.43)	-9.23 (-32.05, 13.59)	-7.44 (-10.66, -4.22)	<0.001
	I^2 = 93%	I^2 = 82%	I^2 = 95%	I^2 = 89%	
V_{50} (%)	-	-1.92 (-7.57, 3.74)	/	-5.28 (-12.27, 1.70)	*
		I^2 = 82%		I^2 = 89%	
LV-D_{mean} (Gy)	-	-12.43 (-19.11, -5.75)	-	-10.20 (-14.32, -6.08)	*
		I^2 = 84%		I^2 = 95%	
LAD-D_{mean} (Gy)	-	-13.88 (-19.84, -7.93)	-	-11.24 (-17.31, -5.61)	*
		I^2 = 90%		I^2 = 99%	
BM					
D_{mean} (Gy)	/	-	-3.00 (-5.25, -0.75)	-2.89 (-4.46, -1.32)	*
			I^2 = 28%	I^2 = 0%	
V_{10} (%)	-	-	-11.22 (-16.34, -6.10)	-10.21 (-13.99, -6.42)	*
			I^2 = 0%	I^2 = 0%	
V_{20} (%)	-	-	-9.68 (-19.28, -0.08)	-10.33 (-16.20, -4.47)	*
			I^2 = 77%	I^2 = 69%	
SC					
D_{max} (Gy)	-13.52 (-23.56, -3.48)	-7.14 (-10.95, -3.34)	-3.86 (-9.39, 1.67)	-6.93 (-9.73, -4.13)	0.097
	I^2 = 81%	I^2 = 92%	I^2 = 89%	I^2 = 91%	

Key: All represent mean and 95%CI (confidence interval); /: represent no one study; -: represent only one study; *: represent included studies less than ten (P <0.05, represent significant publication bias); D_{mean}: mean dose; V_x: percentage of volume receiving ≥x Gy; LV: left ventricle; LAD: left anterior descending artery; BM: bone marrow; SC: spinal cord; Heart-V_{30}, V_{40} observed to have publication bias by Egger's test (P <0.05), there were two (results dose not significantly changed) and zero potential studies added by the trim-and-fill method.

© 2023 Zhou P et al. *JAMA Network Open.*

eTable 7. Pooled Analysis of the Incidence of Toxic Effects in the Proton and Photon Therapy Group (Described as OR)

	Acute	Late	Unknown	Total
RE				
G2+	1.69 (0.76, 3.75)<br>I^2 = 0%	/	-	1.11 (0.76, 1.63)<br>I^2 = 0%
G3+	1.15 (0.40, 3.29)<br>I^2 = 0%	/	-	0.84 (0.48, 1.46)<br>I^2 = 0%
G4+	-	/	-	0.53 (0.02, 13.1)<br>I^2 = Not applicable
RP				
G2+	-	-	-	0.40 (0.17, 0.97)<br>I^2 = 0%
G3+	-	-	-	0.45 (0.12, 1.67)<br>I^2 = 0%
G4+	-	-	-	0.80 (0.07, 8.88)<br>I^2 = Not applicable
PE				
G2+	/	0.55 (0.10, 2.91)<br>I^2 = 35%	-	0.73 (0.32, 1.65)<br>I^2 = 35%
G3+	-	-	-	0.28 (0.06, 1.32)<br>I^2 = 0%
G4+	-	-	-	Not estimable
PCE				
G2+	/	0.15 (0.01, 2.08)<br>I^2 = 71%	-	0.20 (0.04, 0.96)<br>I^2 = 44%
G3+	-	-	-	0.34 (0.06, 2.06)<br>I^2 = 0%
G4+	-	-	-	Not estimable
lymphocytopenia				
G2+	-	/	/	-
G3+	-	/	/	-
G4+	0.35 (0.28, 0.45)<br>I^2 = 0%	/	/	0.35 (0.28, 0.45)<br>I^2 = 0%

Key: All presented as OR (odds ratio) and 95%CI (confidence interval); /: represent no one study; -: represent no sufficient data to analysis; G2+/3+/4+: toxicity grade ≥ 2/3/4; RE: radiation related esophagitis; RP: radiation pneumonitis; PE: Pleural effusion; PCE: pericardial effusion.

© 2023 Zhou P et al. *JAMA Network Open.*

eTable 8. Single-Rate Pooled Analysis for the Incidence of Toxic Effects in Proton Therapy

	Acute	Late	Unknown	Total
G2+				
RE	0.51 (95%CI: 0.35, 0.66)	/	0.46 (95%CI: 0.39, 0.53)	0.50 (95%CI: 0.40, 0.59)
	I^2 = 68.7%		^	I^2 = 60.8%
RP	0.03 (95%CI: 0.00, 0.17)	0.00 (95%CI: 0.00, 0.01)	0.04 (95%CI: 0.02, 0.07)	0.02 (95%CI: 0.00, 0.06)
	I^2 = 91.5%	I^2 = 0%	^	I^2 = 83.4%
PE	/	0.03 (95%CI: 0.00, 0.08)	-	0.04 (95%CI: 0.02, 0.07)
		^		I^2 = 0%
PCE	0.00 (95%CI: 0.00, 0.04)	0.05 (95%CI: 0.01, 0.11)	-	0.03 (95%CI: 0.00, 0.07)
	^	I^2 = 57.8%		I^2 = 62.7%
G3+				
RE	0.08 (95%CI: 0.04, 0.15)	-	0.11 (95%CI: 0.07, 0.16)	0.08 (95%CI: 0.05, 0.12)
	I^2 = 63.6%		^	I^2 = 57.1%
G4+				
lymphocytopenia	0.17 (95%CI: 0.07, 0.30)	/	/	0.17 (95%CI: 0.07, 0.30)
	I^2 = 93.8%			I^2 = 93.8%

Key: CI: confidence interval; /: represent no one study; -: represent no sufficient data to analysis; ^: because included studies ≤ 3 (after double-arcsine transformations); G2/3/4+: toxicity grade ≥ 2/3/4; RE: radiation related esophagitis; RP: radiation pneumonitis; PE: Pleural effusion; PCE: pericardial effusion.

© 2023 Zhou P et al. *JAMA Network Open.*
