# Supplementary material for: Efficacy and Safety in Proton Therapy and Photon Therapy for Patients With Esophageal Cancer: A Meta-Analysis
Source: JAMA Netw Open. 2023 Aug 15;6(8):e2328136. doi: 10.1001/jamanetworkopen.2023.28136 (PMC10427943; doi:10.1001/jamanetworkopen.2023.28136)
Supplement: Supplement 2. — Data Sharing Statement [file jamanetwopen-e2328136-s002.pdf]

Data Sharing Statement

Zhou. Efficacy and Safety in Proton Therapy and Photon Therapy for Patients With Esophageal Cancer. *JAMA Netw Open*. Published August 15, 2023. doi:10.1001/jamanetworkopen.2023.28136

Data
Data available: Yes
Data types: Deidentified participant data
How to access data: zpx2019969@163.com
When available: With publication

Supporting Documents
Document types: None

Additional Information
Who can access the data: Researchers whose proposed use of the data has been approved
Types of analyses: For a specified purpose
Mechanisms of data availability: After approval of a proposal, or with a signed data access agreement
Any additional restrictions: None
